# Analysis of Nearly One Thousand Mammalian Mirtrons Reveals Novel Features of Dicer Substrates

**DOI:** 10.1371/journal.pcbi.1004441

**Published:** 2015-09-01

**Authors:** Jiayu Wen, Erik Ladewig, Sol Shenker, Jaaved Mohammed, Eric C. Lai

**Affiliations:** 1 Department of Developmental Biology, Sloan-Kettering Institute, New York, New York, United States of America; 2 Tri-Institutional Program in Computational Biology and Medicine, Weill Cornell Medical College, New York, New York, United States of America; Thomas Jefferson University, UNITED STATES

## Abstract

Mirtrons are microRNA (miRNA) substrates that utilize the splicing machinery to bypass the necessity of Drosha cleavage for their biogenesis. Expanding our recent efforts for mammalian mirtron annotation, we use meta-analysis of aggregate datasets to identify ~500 novel mouse and human introns that confidently generate diced small RNA duplexes. These comprise nearly 1000 total loci distributed in four splicing-mediated biogenesis subclasses, with 5'-tailed mirtrons as, by far, the dominant subtype. Thus, mirtrons surprisingly comprise a substantial fraction of endogenous Dicer substrates in mammalian genomes. Although mirtron-derived small RNAs exhibit overall expression correlation with their host mRNAs, we observe a subset with substantial differences that suggest regulated processing or accumulation. We identify characteristic sequence, length, and structural features of mirtron loci that distinguish them from bulk introns, and find that mirtrons preferentially emerge from genes with larger numbers of introns. While mirtrons generate miRNA-class regulatory RNAs, we also find that mirtrons exhibit many features that distinguish them from canonical miRNAs. We observe that conventional mirtron hairpins are substantially longer than Drosha-generated pre-miRNAs, indicating that the characteristic length of canonical pre-miRNAs is not a general feature of Dicer substrate hairpins. In addition, mammalian mirtrons exhibit unique patterns of ordered 5' and 3' heterogeneity, which reveal hidden complexity in miRNA processing pathways. These include broad 3'-uridylation of mirtron hairpins, atypically heterogeneous 5' termini that may result from exonucleolytic processing, and occasionally robust decapitation of the 5' guanine (G) of mirtron-5p species defined by splicing. Altogether, this study reveals that this extensive class of non-canonical miRNA bears a multitude of characteristic properties, many of which raise general mechanistic questions regarding the processing of endogenous hairpin transcripts.

## Author Summary

Bulk miRNAs in most animal cells derive from cleavage of hairpin precursor transcripts by the Drosha and Dicer RNase III enzymes. A variety of non-canonical miRNA biogenesis strategies are known, including "mirtrons" for which splicing substitutes for Drosha. However, as non-canonical miRNAs usually account for a minor fraction of total miRNA reads, the alternate pathways are often considered of minor impact. In this study, we describe meta-analysis of >1000 mouse and human small RNA datasets, and surprisingly, find evidence of nearly 1000 mirtron using stringent criteria, half of which were novel annotations. Therefore, mirtrons comprise a substantial fraction of total Dicer substrates. We use this catalog to perform diverse sequence and structural analyses of mirtron loci that differentiate them from bulk introns and also to perform diverse comparisons of mirtrons and canonical miRNAs. Surprisingly, we find that many seemingly fundamental features of miRNA genes, which have been well-studied over the years, are not actually general features of miRNAs, but specifically those of hairpins that transit the Drosha-Dicer pathway. Instead, the nearly 1000 splicing-derived miRNAs reveal expanded and/or distinct properties encompassed by endogenous Dicer substrates in mammalian cells. These features reveal hidden impacts that shape miRNA processing, function, and evolution.

## Introduction

MicroRNAs (miRNAs) are an extensive class of ~22 nucleotide (nt) regulatory RNAs, generated from hairpin precursors, that figure prominently into post-transcriptional regulatory networks in diverse eukaryotes [1,2]. Biochemical studies of the initially recognized set of miRNAs revealed their biogenesis by a common mechanism [3]. For example, miRNAs in animals are typically generated via stepwise cleavage by two RNase III enzymes: nuclear Drosha cleaves primary miRNA transcripts into pre-miRNA hairpins, while cytoplasmic Dicer cleaves pre-miRNAs into small RNA duplexes [4]. Following duplex loading into an Argonaute (Ago) protein, one strand of the duplex (the mature miRNA) is preferentially retained relative to its partner strand (the miRNA* or "star" species), and guides the Ago complex to target transcripts [5].

**Fig 1:**
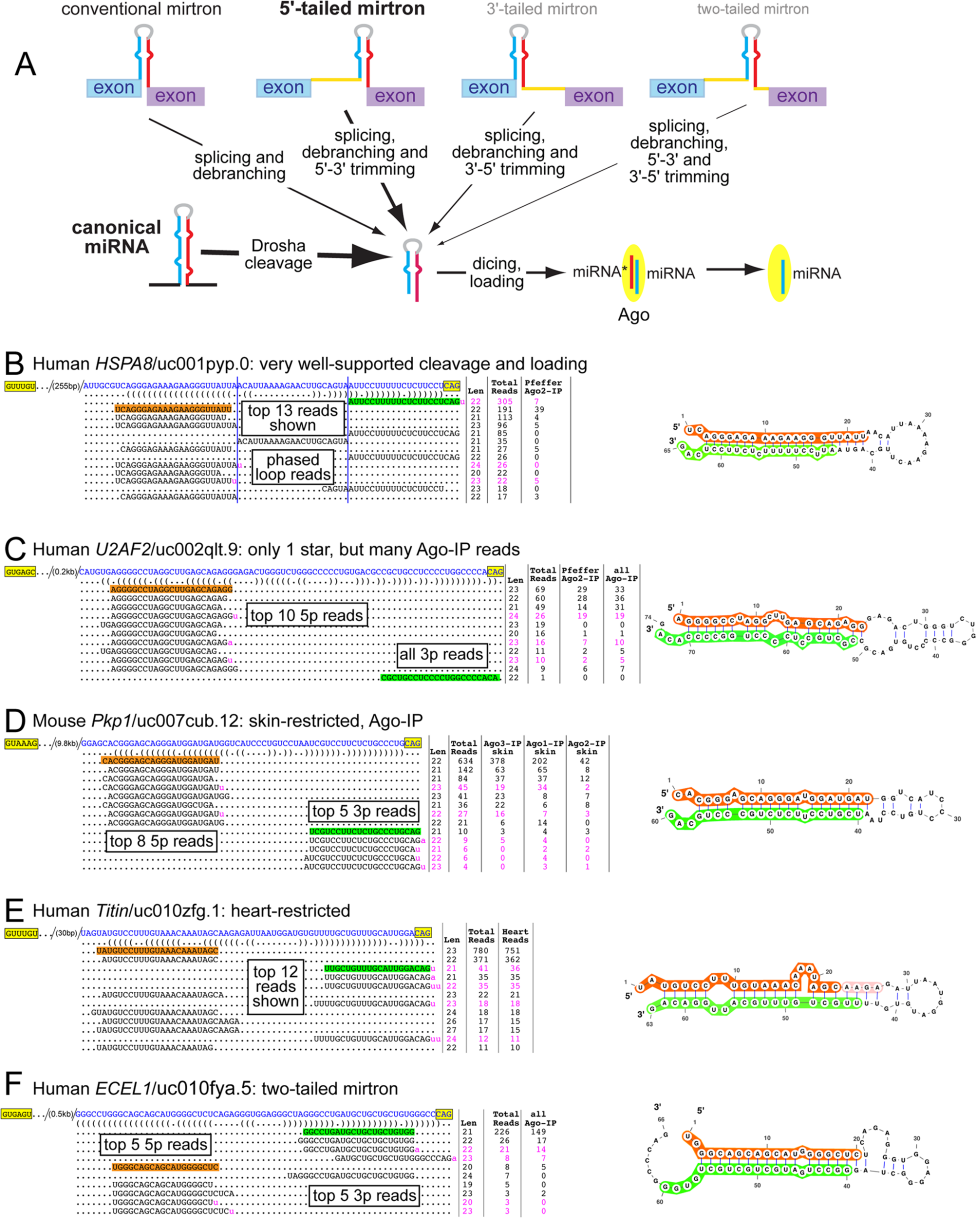
Examples of novel mirtrons confidently annotated in this study. (A) Drosha-mediated and splicing-mediated pathways for the generation of Dicer-substrate pre-miRNA hairpins. For both canonical miRNA loci and mirtron loci of all four classes, the critical judgement for their annotation is whether the small RNA evidence supports the notion that their progenitor hairpins were subject to Dicer cleavage to generate specific miRNA/miRNA* (star) duplexes. The thickness of the arrows leading to Dicer indicates the relative number of substrates generated by each pathway. (B) Mirtron supported by especially robust evidence, including the presence of both miRNA and star reads in Ago-IP data and the recovery of abundant phased loop reads. Note that loop reads were not found in the Ago-IP data, providing evidence for the selection of specific species in Ago complexes. (C) A mirtron locus lacking substantial "star" read evidence, whose confidence is bolstered by hundreds of reads in Ago-IP libraries. (D) Example of a skin-restricted mirtron whose reads are highly represented in Ago1-IP, Ago2-IP and Ago3-IP data. (E) Example of a heart-restricted mirtron. Over 96% of reads collected from nearly 700 human small RNA datasets were from heart. (F) Example of a "two-tailed" mirtron supported by abundant Ago-IP reads. We infer that generation of the pre-miRNA involves splicing followed by removal of both 5' and 3' tails (see A).

Following the delineation of the canonical miRNA biogenesis pathway, non-canonical loci were recognized to be independent of enzyme(s) believed obligate for the production of functional miRNAs [6]. The major alternative pathway utilizes splicing to generate pre-miRNA hairpin mimics termed mirtrons, thereby bypassing Drosha [7,8]. Mirtrons come in several flavors (Fig 1A), depending on how directly the pre-miRNA hairpin ends are defined by splicing [9]. With conventional mirtrons, both hairpin ends are generated by splicing and form a short 3' overhang that is appropriate for nuclear export and dicing. With 3'-tailed mirtrons, the 5' hairpin end corresponds to the 5' splice donor, but an unstructured region follows to the 3' splice site. Where studied in *Drosophila*, the 3' tail is resected by the RNA exosome to generate the pre-miRNA substrate [10]; this mechanism has not yet explicitly been shown in vertebrates. Mirtrons with an opposite polarity also exist, namely 5'-tailed mirtrons where an unstructured region precedes a hairpin that resides precisely at the 3' end of the intron [11–13]. The enzyme(s) responsible for resection of 5' tails has not yet been identified. Recently, two intronic miRNAs (*mir-3062* and *mir-7661*) were proposed to be dependent on splicing, even though neither of their hairpin termini directly abut splice sites [14]. For such "two-tailed mirtrons", neither the nucleases nor the order of tail removal is known.

Non-canonical miRNA loci are often collectively considered as a minor class, as bulk miRNA reads in most cells and tissues derive from canonical loci. Nevertheless, there are situations where reads from non-canonical loci are not only substantial, but can even be functionally predominant. For example, the unusual Dicer-independent locus *mir-451* obligately depends on slicing by Ago2 for its maturation [15–17], and this miRNA is one of the top-expressed miRNAs in erythrocytes [18]. As another example, while miRNAs are present in mouse oocytes, they appear to be functionally inactive in this setting [19,20], leaving endo-siRNAs as the main functional Dicer-substrate small RNA in mouse oocytes [21–23]. The net functional contribution of non-canonical miRNA pathways is further hinted at by phenotypic discrepancies amongst mutants of different core components of the canonical miRNA biogenesis pathway [24].

Another way to evaluate the contribution of non-canonical miRNA loci is by the numbers of confidently annotated loci. The number of miRNA genes in any species is under constant revision, and depends on the types of data and stringency of annotation applied. Some studies of mammalian miRNAs have varied from ~500 [25] to several thousand [26,27]; these studies have typically focused on canonical miRNA loci. Although these estimates range over many fold, they are in any case only a small fraction of the 100,000s to millions of plausible miRNA-like hairpins that can be computationally predicted in mammalian genomes. Since the majority of these genomes is transcribed [28,29], this suggests that the general limitation of genomic hairpins to generate miRNAs is not at the level of transcription, but instead reflects our insufficient knowledge of features required for miRNA biogenesis. Perhaps surprisingly, then, we recently used strigent annotation criteria to report well over 400 novel loci that generate splicing-derived miRNAs in mouse and human [13]. Therefore, even though the levels of small RNAs generated by most mirtrons is modest, they actually comprise a substantial fraction of clear Dicer-substrate loci in mammals. Of note, the levels of bulk mirtrons are comparable to many hundreds of other recently-emerged mammalian miRNAs [13]. These observations suggest that non-canonical pathways may have substantial impact on the dynamics of miRNA evolution and species-specific regulation [13,30].

In this study, we mined additional deep sequencing data to identify ~500 novel mammalian mirtrons using stringent criteria. We use this expanded set of ~1000 loci to identify the characteristic sequence, structural, and genomic features of mammalian mirtrons. Although mirtrons generate miRNA-class regulatory RNAs via pre-miRNA dicing, as with canonical miRNAs, we find that mirtron hairpins and their small RNAs have a multitude of distinctive properties compared with canonical miRNAs. These reveal novel aspects of endogenous Dicer substrates that are recognized when Drosha is not the initiating nuclease, and implicate unexpected pathways that process the 5' and 3' ends of pre-miRNAs.

## Results

### Updated annotation of mammalian mirtrons reveals hundreds of novel loci

We recently analyzed a large collection of mouse and human small RNA data to identify nearly 500 mirtrons, i.e. loci that generate splicing-derived miRNAs [13]. We defined these as intronic hairpins for which one or both hairpin termini abut intron junctions, and that are associated with confident evidence for endogenous dicing into small RNA duplexes. These predominantly comprised 5'-tailed mirtrons, with smaller numbers of conventional mirtrons and a minor set of 3'-tailed mirtrons.

Our previous study also classified many candidate splicing-dependent hairpins with more limited expression evidence, and these followed a similar distribution of mirtron subtypes as the confident loci [13]. Given the compelling read evidence of many of these candidates, which nonetheless did not meet our stringent annotation criteria, we inferred many of them might be considered genuine mirtrons with additional small RNA data. Notably, even with recent state-of-the-art efforts for mammalian miRNA annotation [26], the distinct structural features of mirtrons (i.e. short hairpins) means that they have generally escaped efforts for miRNA annotation to date. Moreover, the recognition of "two-tailed mirtrons" [14] and mirtron trimming pathways [31] suggested that it is necessary to consider broader annotation criteria to fully capture the diversity of splicing-dependent small RNAs (Fig 1A). Therefore, we also paid attention to intronic read pileups in proximity to, but that did not directly abut, splice junctions.

Many additional small RNA datasets have since become available, covering a broader range of tissue and cell types than we considered previously. We processed 189 additional human and 125 mouse datasets, and aggregated these with other data (S1 Table); in total over 1000 deeply sequenced libraries. The newly-analyzed data include many Ago-IP libraries, which have particular power to increase annotation confidence. This was particularly relevant to mouse, for which we incorporated substantial Ago-IP datasets from brain [32], T cells [33], skin cells [34] and NIH-3T3 cells [35]. We then re-ran our pipeline to identify candidate mirtrons, and vetted these extensively by individual inspections of their read evidence.

Because of the greater read depth and library variety now analyzed, we were able to increase the stringency of our annotation criteria from our previous study [13] (see Materials and Methods). Some loci were supported by ancillary layers of evidence for their biogenesis. For example, for the novel mirtron located in human *HSPA8*, we recovered not only precise miRNA and star reads, but also the phased loop read representing the Dicer byproduct (Fig 1B). Notably, while both its miRNA and star reads accumulated in Ago2-IP data, its cloned loop species did not. Although special hairpin loops associate with Ago proteins [36,37], pre-miRNA loop species are typically rejected from Argonaute complexes.

We also recognized needs for flexible annotation criteria. In particular, the existence of substantial reads in Ago-IP libraries seemed appropriate to classify miRNA-generating loci, even when certain other features were suboptimal. For example, we typically demand that both miRNA and star species be multiply cloned to annotate mirtrons and canonical miRNAs with confidence [13,38]. However, some highly cloned reads from intron terminal hairpins exhibited few companion star species, and therefore did not meet our previous duplex criterion. Although it seemed prudent to consider these only as "candidates", strongly asymmetric strand selection might preclude accumulation of star species [39,40]. We elevated the status of a small set of candidate loci with modest numbers of star species, if they generated ≥100 total mature arm reads of which ≥20 reads were from Ago-IP datasets (see Materials and Methods). Fig 1C depicts a novel mirtron hairpin within human *U2AF2* with hundreds of mature reads in aggregate data, including >100 Ago2-IP reads, but only one star read.

In total, the greater library depth and Ago-IP datasets allowed us to "upgrade" many mirtrons from our previous lists of candidate loci [13]. As well, we identified some mirtrons with exquisite tissue-specificity, which required the analysis of new libraries (e.g. Fig 1D and 1E). Reciprocally, our refreshed efforts barely necessitated that we "downgrade" any previously annotated mirtrons; that is, nearly all of them looked even more robust with larger data. The vast majority of novel mirtrons in both human and mouse are 5'-tailed loci, with modest numbers of conventional mirtrons and minor sets of 3'-tailed loci and two-tailed loci. In particular, we expand the latter class from two reported earlier to 24. A robust example of a novel two-tailed mirtron is shown in Fig 1F, for which small RNAs from *ECEL1* dominantly accumulated in Ago-IP data.

**Fig 2:**
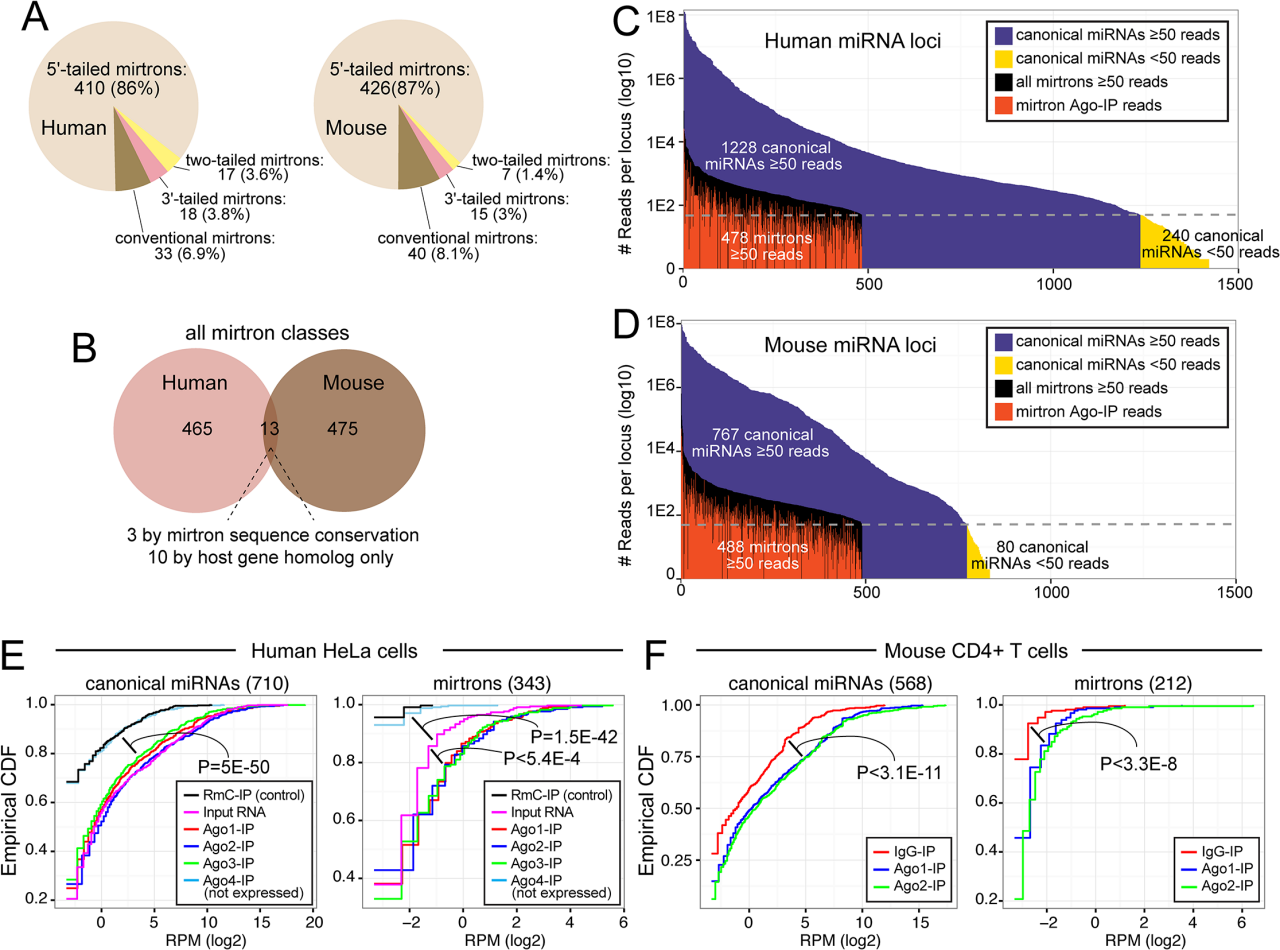
Greatly expanded annotations of human and mouse mirtrons. (A) Numbers of splicing-derived miRNAs in human and mouse, categorized as conventional, 5'-tailed, 3'-tailed, and two-tailed mirtrons. Most of the miRNAs newly annotated in this study were 5'-tailed mirtrons, reflecting their status as the dominant mirtron class in human and mouse. (B) Few mirtrons were annotated from small RNA data in both mouse and human, and only a subset of these were constrained in primary sequence. (C, D) Human and mouse mirtrons are generally modestly expressed, but were annotated to higher levels of evidence than hundreds of human and mouse miRNAs in the miRBase registry (i.e. that have <50 reads in the aggregate data analyzed in this study). Most mirtrons were supported by evidence from Ago-IP datasets (red bars). (E, F) Cumulative distribution function (CDF) plots of enrichment of canonical miRNAs and mirtron-derived miRNAs in Ago complexes. (E) Analysis of human small RNAs. Rat RmC was used as control IP; since Ago4 is not expressed in HeLa cells, it effectively serves as another control IP. Canonical miRNAs were enriched in Ago1-3-IP data as well as input RNA (which is mostly composed of Ago-bound miRNAs), relative to control IP data. Mirtron-derived small RNAs showed similar Ago-IP enrichment, except that they also exhibited enrichment between Ago1-3-IP and input RNA libraries. (F) Analysis of mouse small RNAs shows similar enrichment of canonical miRNAs and mirtron-derived small RNAs in Ago1 and Ago2 complexes relative to control IgG complex.

In total, we confidently identify 478 human (242 novel to this study) and 488 mouse (248 novel to this study) loci as generating splicing-derived, diced small RNA duplexes. We summarize the tallies of the various classes of mirtron loci in human and mouse in Fig 2A. Additional information regarding the evidence supporting these loci is provided in S2 and S3 Tables, and extensive information on the reads and libraries that map to all human and mouse mirtrons can be explored in the Supplementary Websites. Curiously, hardly any of these stringently-annotated mirtrons are conserved between human and mouse (Fig 2B and S1 Fig). Even amongst the modest number of positionally conserved mammalian mirtrons that generate small RNAs, only in a few of these cases do the mirtron hairpins exhibit a classic "saddle-shaped" pattern of evolution indicative of evolutionary constraint as trans-regulatory species [41,42] (S2 Fig).

### Overall expression properties of the mammalian mirtrons

We emphasize our conservative procedures in annotating novel mirtrons. Beyond incorporation of strict duplex evidence, the minimum numbers of reads supporting our mirtron annotations are comparable to or exceed those of many extant canonical miRNAs (Fig 2C and 2D). In fact, 320 canonical human and mouse miRNA loci in the miRBase repository have less read support than our set of ~1000 mirtrons. As well, the vast majority of our mirtrons contribute reads to Ago-IP libraries (Fig 2C and 2D).

To investigate this in more detail, we selected human and mouse datasets with rigorous control and multiple Ago-IP libraries, and compared the behavior of canonical miRNAs and mirtrons. Meister and colleagues analyzed endogenous human Ago1-4 contents from HeLaS3 cells, and compared these to total RNA as input and control IP using rat RmC antibody [43]. Since Ago4 is not expressed in these cells, Ago4-IP and RmC-IP show the same background levels of miRNA accumulation, whereas input RNA and Ago1/2/3-IP datasets all show enrichment for canonical miRNAs (Fig 2E). The similar representation of miRNAs in the input and Ago-IP datasets is likely due to the fact that canonical miRNAs are the predominant small RNA species in these cells, regardless of Ago purification. Although mirtrons are overall lower-expressed, we find that they actually exhibit more informative segregation across these datasets. That is, RmC-IP and Ago4-IP showed almost no mirtron reads, whereas the input library had intermediate levels relative to Ago1/2/3-IP libraries (Fig 2E). Therefore, mirtron-derived small RNAs are genuinely enriched in Ago complexes. We also examined data from Corey and colleagues, who generated IgG-, Ago1- and Ago2-IP datasets from mouse CD4+ T cells [33]. Although mirtrons accumulated to much lower levels than canonical miRNAs, both classes of miRNAs were significantly enriched in both Ago1-IP and Ago2-IP datasets relative to IgG-IP library (Fig 2F). The behavior of individual canonical miRNA and mirtron loci in these control and Ago-IP datasets is provided in S4 and S5 Tables.

Together, these analyses provide evidence that mirtrons generally generate small RNAs with the biochemical properties of genuine miRNAs. Still, as our effort relied upon a large meta-analysis, a question arises how many loci are due to aggregation of a few reads in each of many libraries. To address this, we plotted the maximum number of reads mapped to mouse and human mirtrons across individual libraries (S3A and S3B Fig). These analyses showed a few dozen mirtrons fit the bill of having only single-digit reads in any particular dataset, even though all passed a 50 read minimum cutoff.

It might be that some mirtrons are expressed lowly, but tissue-specifically. We attempted to address this scenario by grouping similar libraries-of-origin and repeating the above analysis. This treatment of data substantially reduced the numbers of these most modestly-represented mirtrons (S3C and S3D Fig), consistent with the notion that many of them might be spatially restricted. We explored this further by comparing the library distribution of mirtrons and canonical miRNAs. We observed that mouse and human mirtrons were biased to present their dominant expression in a small number of libraries, whereas canonical miRNAs tended to accumulate more broadly across many libraries (S3E and S3F Fig). The interpretation of this analysis may be biased by the overall lower accumulation of mirtrons relative to canonical miRNAs. Nevertheless, we observed many mirtrons with exquisite tissue restriction. For example, even though we analyzed many hundreds of human and mouse libraries, small RNA reads from mirtrons in mouse *Pkp1* (Fig 1D) and human *Titin* (Fig 1E) were recovered nearly exclusively from skin Ago-IP or heart total RNA data, respectively.

In summary, the splicing pathway contributes strongly to the cellular pool of endogenous Dicer substrates in different mammals. The overall view is that the many hundreds of mirtrons are expressed in a modest range, but a range that encompasses a substantial set of modestly-expressed mammalian miRNAs. We subsequently performed all of our analyses independently on these mostly non-overlapping sets of mouse and human mirtrons, from which we could derive general features of mammalian mirtrons and compare them to Drosha-substrate miRNAs.

### Mirtron expression is correlated with host genes, but these can be uncoupled

The majority of intronic miRNAs are found on the sense transcribed strands of host protein-coding transcripts [44], and the accumulation of intronic miRNAs and host mRNAs is overall correlated [45]. Nevertheless, intronic miRNAs are not necessarily produced in the course of transcribing host protein-coding genes, since miRNA biogenesis can be regulated post-transcriptionally. Conversely, some miRNAs might be transcribed from intronic promoters, and thus their expression might be decoupled from their inferred host genes [46,47]. Mirtron biogenesis is presumably more intimately coupled with the maturation of their host genes. Nevertheless, their accumulation might not necessarily mirror each other, for the same reasons that apply to canonical miRNAs.

To test the expression correlation of mirtrons with their host genes, we utilized publicly available datasets of tissue-specific mouse and human mRNA-seq data (see Materials and Methods). We began by comparing the correlations of mRNA and small RNA accumulation across tissues. When plotted as cumulative frequency distributions, we observed strong positive correlations between mRNA and miRNA in both mouse (p<5.05E-9) and human (p<1.62E-5) data, compared to the background distributions obtained when cognate tissue identities were shuffled (S4 Fig). Given that these analyses involved tissue samples and expression data prepared independently and that may not be directly comparable, the extent of correlations is likely an underestimate.

**Fig 3:**
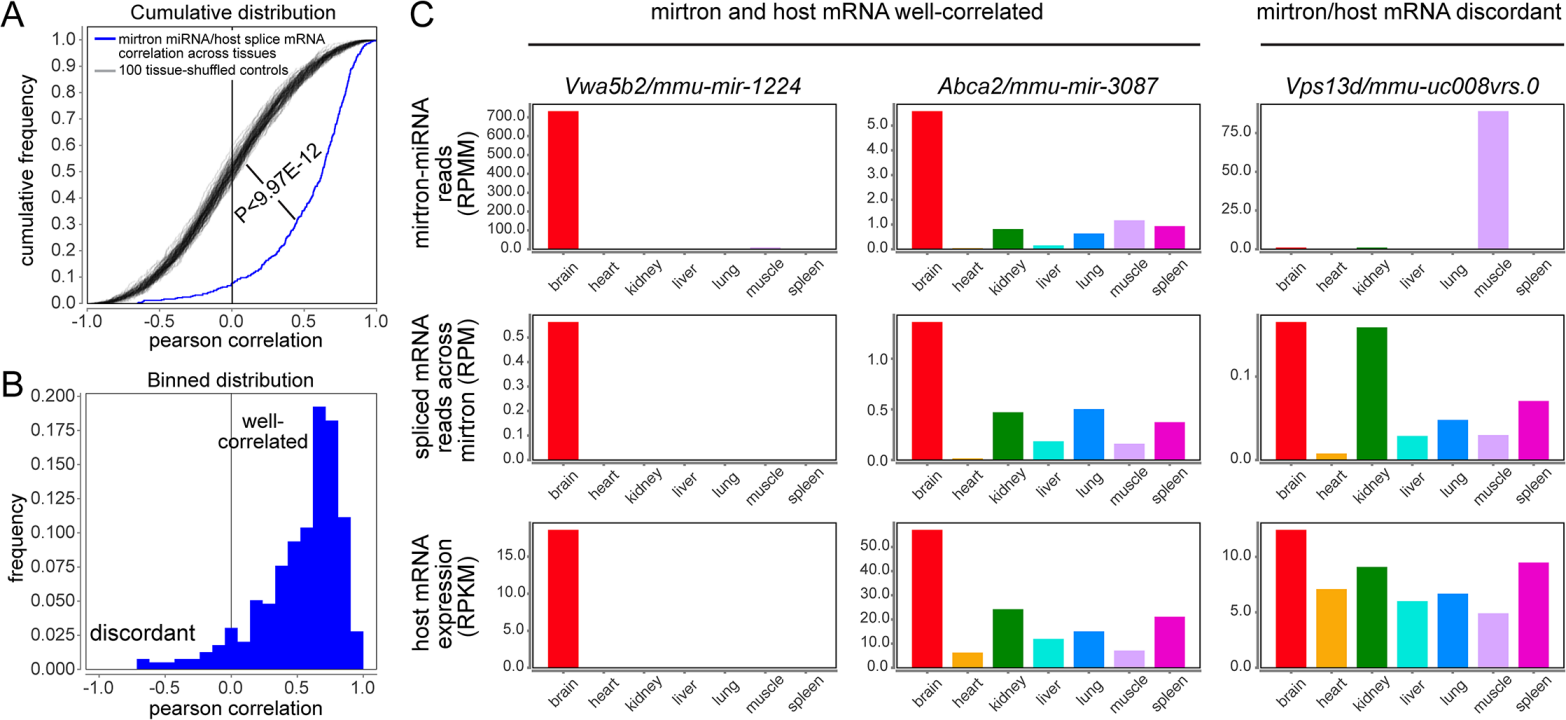
Correlation of mirtron and host gene expression. (A) We calculated the Pearson correlation coefficients of the accumulation of mouse mirtron-derived small RNAs and spliced RNA-seq reads directly flanking the mirtron across seven tissues. We also performed 100 control comparisons where the tissue origins were shuffled. The cumulative distribution function (CDF) of these correlations was plotted, and observed to be significantly positively correlated (by Mann-Whitney U-test). (B) The binned distribution of mirtron/mRNA Pearson correlation coefficients was plotted. This visualization emphasizes their positive correlation, but also highlights a subset of discordant loci. (C) Examples of correlated and discordant expression of mirtron-derived miRNAs and host mRNAs across tissues. We show host level gene expression as reads per kilobase of transcript per million mapped reads (RPKM) and the spliced exonic reads that directly cross the mirtronic locus as reads per million mapped reads (RPM). Mirtron-derived miRNAs are quantified as reads per million mapped miRNA reads (RPMM).

One way the true correlation might be underestimated in the above analysis, utilizing the mRNA expression of all exons, would be if mirtrons were generated from specific mRNA isoforms. We attempted to remedy this by performing correlation analysis only with spliced mRNA reads that span exon-exon junctions across mirtrons. This analysis generated a positive correlation in the human data, but it was less significant than with the gene level analysis. We can easily rationalize this, however, due to undersampling, since we observed some human loci with no spliced RNA-seq reads across a given mirtron locus (S5 Fig). However, the available mouse RNA-seq data (~3.8 billion from 7 tissues; ~550 million reads/tissue), were much deeper than the human data (370 million reads from 6 tissues; ~61 million reads/tissue). Indeed, the superior depth of the mouse data for spliced reads across mirtrons substantially improved the correlation of host gene-mirtron expression. As shown in the CDF plot (Fig 3A), there is a strong bias for well-correlated pairs (p<9.97E-12).

A similar conclusion can be seen via the binned data plot (Fig 3B). Interestingly, this visualization makes apparent a subset of loci for which the accumulation of spliced flanking exon reads and mirtron-derived miRNAs are particularly uncorrelated (Fig 3B). We present some individual cases of highly correlated and uncorrelated host mRNA/mirtron mouse expression profiles in Fig 3C and show additional mouse and human examples in S5 Fig. In many of these instances, we observe that the host gene is expressed more broadly than where the mirtron-derived small RNAs accumulated. Such cases are suggestive of post-transcriptional regulation of mirtron biogenesis.

### Host introns of mirtrons and tailed mirtrons exhibit biased lengths

**Fig 4:**
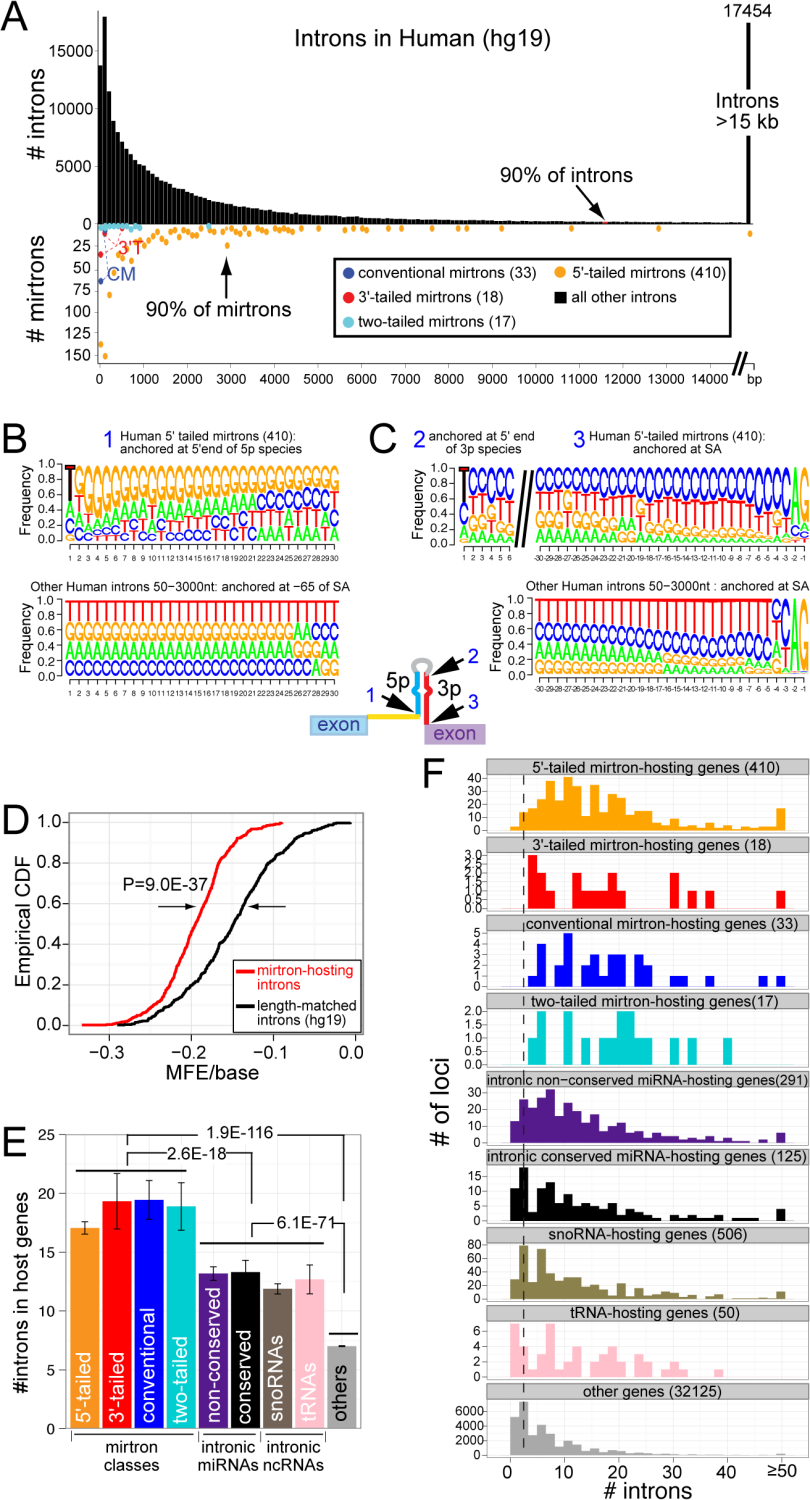
Sequence and length properties of mirtron-containing introns. (A) Comparison of mirtron-bearing introns with total introns in human. The distribution of total intron lengths is much broader than for mintrons. The dominant class of 5' tailed mirtrons derives mostly from introns that are <3kb in length, while the 3'-tailed mirtrons and conventional mirtrons derive from very short introns. (B, C) Nucleotide bias of small RNAs from 5'-tailed mirtrons. Three anchor points were considered, as schematized on the 5'-tailed mirtron model in the center (1, 2, 3, arrows). (B) Biased nucleotide identities of mirtron-5p reads from the dominant class of 5'-tailed mirtrons. Compared to an equivalent sequence range of control introns of similar length, mirtron-5p reads exhibit substantial 5'-U bias and overall enrichment of G across their lengths. The G bias is greater in the 5' than 3' regions of the mirtron-5p reads, and is not evident in bulk intron sequences downstream of their ~22 nt lengths. (C) Biased nucleotide identities of mirtron-3p reads from the dominant class of 5'-tailed mirtrons. Compared to control introns, there is substantial 5'-U bias (evident with aligning by their 5' ends) and substantial C-bias across their length. Note that the bulk introns exhibit polypyrimidine tracts upstream of the splice acceptor site (YAG), but mirtrons exhibit greater representation of C while control introns show greater bias for U. (D) Mirtronic regions exhibit much lower minimum free energy (MFE) than control intronic regions. CDF (cumulative distribution function) is plotted for MFE/base distribution. (E) All four classes of mirtrons are hosted by genes with greater numbers of introns than average genes. Various classes of other intronic non-coding RNAs (e.g. tRNAs, snoRNAs, and either conserved or non-conserved canonical miRNAs) typically reside in genes with larger numbers of introns than bulk genes, but their averages are intermediate to all classes of mirtrons. (F) Bar graphs that emphasize the individual properties of genes that host various classes of non-coding RNAs. It is evident that the all four classes of mirtrons have a broader distribution of intron numbers relative to other types of non-coding RNAs.

In analyzing the structural features of mirtrons, we sought properties that might distinguish them from other mammalian introns (i.e., "bulk" introns). We compared the length distribution of introns that hosted mirtrons and tailed mirtrons relative to all other introns, plotting them in bins of 100 nt and pooling remaining introns >15 kb and >10 kb in length in human and mouse, respectively. In both the human and mouse genomes, the number of introns is greatest for the 100–200 nt window, gradually tailing off from there with increasing lengths (Fig 4A and S6A Fig). Conventional mirtrons were mostly restricted to the <100 nt window, in both mouse and human. Although the certain 3'-tailed mirtron loci ranged into larger sizes, these mostly derived from similar sizes as conventional mirtrons. Therefore, although the number of 3'-tailed loci was modest, their similar length properties in both mammalian species suggests that the capacity for 3' trimming does not dramatically increase the range of introns that mirtrons can occupy.

The 5'-tailed mirtrons exhibited distinct properties from the other mirtron classes. On the one hand, they also exhibited a preference for relatively smaller introns in both human and mouse. Amongst all 100nt bins of intron sizes, the largest numbers of 5'-tailed mirtrons derived from <100nt and 100–200 nt introns. Still, there were significant populations of 5'-tailed mirtrons >1 kb, with ~90% of these loci cumulatively derived from introns up to 3 kb in length (Fig 4A and S6A Fig). Despite this flexibility in length, the frequency of 5'-tailed mirtrons in progressively larger introns decreased more quickly than the overall length profile of bulk introns, with 90% of all introns are contained within the <10 kb range (Fig 4A and S6A Fig). This implies that 5'-tailed mirtrons arise more readily in introns of relatively smaller length, mostly <3 kb.

The length bias of 5’-tailed mirtrons suggests that the 5' resection pathway is progressively less effective on tails longer than a few kb. Based on our identification of several examples of potentially alternative unannotated splicing of mirtron precursors, it is possible that some instances of 5'-tailed mirtrons in long annotated introns may actually derive from alternative splicing of shorter introns. The longest annotated host intron of a 5'-tailed mirtron belongs to *mir-5129*, located in an 87 kb intron of mouse *Zeb2* [48]. Given that the next largest host intron of a tailed mirtron is ~36 kb (mouse *Klf7*), and the vast majority of 5'-tailed mirtrons reside in introns of up to a couple kb, one wonders whether *mir-5129* is generated directly from splicing of such a long intron. Examination of the UCSC reveals a population of uncharacterized *Zeb2* ESTs that utilize an internal 5' splice site joining the *mir-5129* 3' splice site, resulting in a ~32 kb intron. While this is still rather extreme, it supports the possibility that "long" introns bearing 5’-tailed mirtrons may be subject to alternative splicing of shorter unannotated introns, or perhaps, some type of recursive splicing [49,50].

### Sequence characteristics of mirtrons distinguish them from bulk introns

We next examined the primary sequence properties of mirtrons. We were again drawn to the 5'-tailed mirtrons because of their abundance. We generated sequence logos of 422 human and 429 mouse 5'-tailed mirtron hairpins that were anchored at three different locations: by the 5' end of the 5p species, by the 5' end of the 3p species, and by the 3' end of the 3p species (i.e., by the AG splice acceptor). We compared these to the nucleotide content of bulk introns 50–3000 nt in length, which comprise the strong majority of 5'-tailed mirtron host intron lengths. To provide a proxy for the start of the mirtron hairpin in bulk introns, we aligned these introns from the -65 position from the end of the intron, corresponding to the average distance of the start of 5'-tailed mirtrons from their intron ends in human/mouse.

Several characteristics of 5'-tailed mirtrons emerged from this analysis. First, the 5' ends of both 5p and 3p species exhibit 5' uridine for a majority of loci in both human (Fig 4B) and mouse (S6B Fig). Such 5' U bias is typical for canonical miRNA species, but was not observed in comparable regions of bulk introns. These data support the notion that these processed small RNAs of 5'-tailed mirtrons have been selected according to similar rules as canonical miRNAs, likely including the preference of the MID domain of mammalian Ago2 to select small RNAs with 5'-U [51]. Second, the duplex regions of 5'-tailed mirtrons are characterized by high guanine content on their 5p arms and high cytidine content on their 3’ arms, resulting in a high degree of G:C pairing (Figs 4B and 4C and S6B and S6C). Although the 3’ ends of introns generally contain polypyridimine tracts, they exhibit somewhat greater uridine bias, whereas the 3' ends of 5'-tailed mirtrons exhibit substantial cytidine enrichment in human (Fig 4C) and mouse (S6C Fig). The region of bulk introns from -65 to -35 of the splice acceptor exhibits little nucleotide bias, making the enrichment of G residues in 5'-tailed mirtron-5p arms particularly striking.

The high GC content of all classes of mirtrons suggested that they adopted much more stable structures than bulk introns. We compared all classes of mirtron hairpins with a length-matched distribution of control introns (see Materials and Methods). These analysis showed that mirtrons indeed collectively exhibit far lower minimum free energy (MFE) per base than do non-mirtronic introns in human (Fig 4D) and mouse (S6D Fig). Altogether, these observations provide evidence that mammalian mirtrons and tailed mirtrons emerge from an intron subpopulation with length and sequence characteristics that are distinct from bulk mammalian introns.

### Mirtron-hosting genes contain more introns than genes hosting other miRNAs or other ncRNAs

We next studied the numbers of introns in genes that host mirtrons. The null hypothesis might be that mirtrons are equally eligible to arise in most genes, with some expectation that genes with more introns might have more chances to harbor an intronic non-coding RNA locus. Directed expression tests in *Drosophila* [7,8,52] and mammalian cells [13,53,54] demonstrate that functional mirtrons can be generated from single intron constructs, and the average mammalian genes have ~7 introns, which might provide reasonably abundant opportunities for their emergence. However, we observed that the average numbers of introns in mirtron-hosting genes far exceeded the genomewide averages in both human (Fig 4E) and mouse (S6E Fig). In fact, these trends were true for all three classes of splicing-derived miRNAs, as conventional mirtrons, 5'-tailed mirtrons, 3'-tailed mirtrons, and two-tailed mirtrons all derived from host genes that bore 2.5–3 times the average numbers of introns (p = 1.9E-116 and p = 6.4E-118 in human and mouse, respectively). This may imply an evolutionary advantage for the potential of mirtron emergence in genes with larger numbers of introns.

We analyzed in detail the distribution of intron numbers for protein-coding host genes for the different mirtron classes and for a selection of other non-coding RNAs (ncRNAs), including canonical miRNAs, snoRNAs and tRNAs. In particular, since mirtrons are generally recently-emerged, we split up the canonical miRNAs into ones that are well-conserved across mammals and ones that are specific to rodents or to primates. Both classes of miRNAs and the other ncRNAs derive from genes with intermediate numbers of introns, relative to the different classes of mirtrons and bulk genes in human (Fig 4E). The same trends were also observed in mouse (S6E Fig).

We gain additional information by binning each class of non-coding element. We clearly observe that the distribution of host gene intron numbers for all classes of mirtrons is larger and broader than for bulk genes. In contrast, while the distribution of intron numbers for genes that host various types of ncRNAs is broader than that of bulk genes, the preferred host intron numbers for tRNAs, snoRNAs and conserved miRNAs overlaps substantially with bulk genes (Fig 4F and S6F Fig). Therefore, all classes of mirtrons appear to emerge preferentially from a population of protein-coding genes that is distinct from the habitat of many other types of intronic ncRNAs.

### Splicing can generate longer pre-miRNA hairpins than does Drosha cleavage

Plant miRNAs exhibit great heterogeneity in their hairpin lengths, with some spanning hundreds of nucleotides [3]. Select canonical miRNA hairpins in *Drosophila* are also exceptionally long [55]. For example, the *pre-mir-989* hairpin is 143 nt in length, inclusive of the cloned miRNA/star species. We emphasize that *mir-989* is a canonical miRNA that generates a typical AGO1-loaded miRNA, since some other "long miRNAs" in *Drosophila* are actually members of the hpRNA class that generates endo-siRNAs [56]. In mammals, pre-miRNAs for confidently annotated loci rarely exceed 80 nt (http://www.mirbase.org/). Potential explanations for this restriction are that it reflects preferred substrate requirements of Dicer, or perhaps a need to avoid inadvertent activation of the interferon pathway, which recognizes dsRNA non-specifically.

Amongst miRNAs annotated prior to 2012, the only pre-miRNA longer than 100 nt (i.e. the hairpin length inclusive of the cloned miRNA/star species) were the human loci *mir-548o* and *mir-1236*, and the mouse loci *mir-3102*, *mir-702* and *mir-1983*. The veracity of *mir-548o* is potentially questionable since all reads ascribed to this locus map to multiple genomic locations, many of which exhibit more compact hairpins (http://www.mirbase.org/). Curiously, the remaining >100 nt pre-miRNAs are all Drosha-independent, with *mir-1983* deriving from a tRNA and the others deriving from splicing [11,25,42]. These observations might imply that alternative sources of pre-miRNAs may have more flexible hairpin lengths than does the canonical biogenesis pathway.

**Fig 5:**
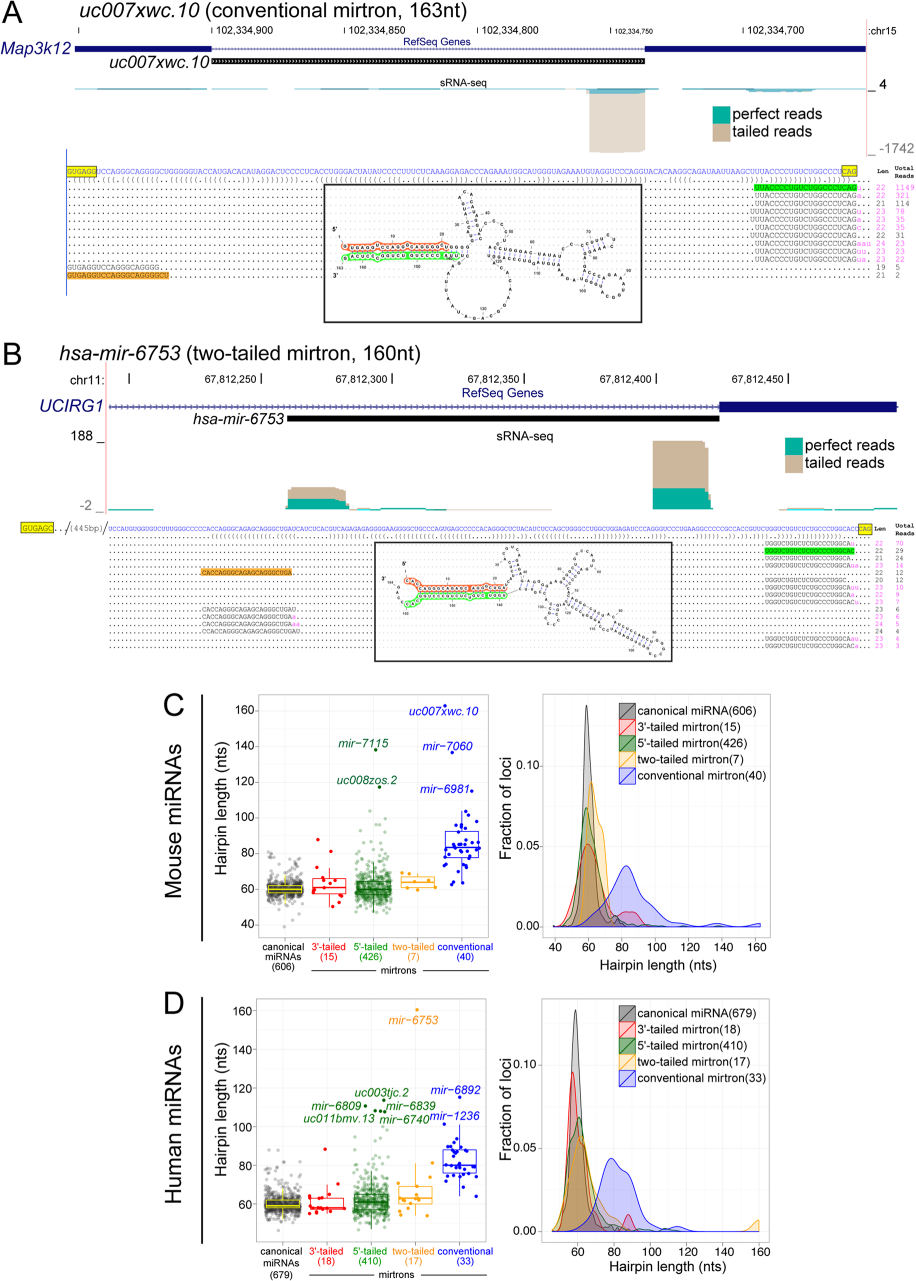
Broader distribution of hairpin lengths in mirtrons vs. canonical miRNAs. (A) Example of a conventional mirtron with extremely long pre-miRNA hairpin. (B) Example of two-tailed mirtron with extremely long pre-miRNA hairpin. In both cases, small RNA reads were recovered specifically from the genomically distant miRNA/star duplexes. (C) Analysis of mouse pre-miRNA lengths. The left plot illustrates individual loci, while the right plot summarizes their overall behavior. Canonical miRNAs exhibit a very tight distribution with no pre-miRNAs greater than 82 nt. The average lengths of 3'-tailed, 5'-tailed, and two-tailed mirtron pre-miRNAs are similar to canonical pre-miRNAs, but 5'-tailed mirtrons exhibit a noticeably broader length distribution. Conventional mirtrons exhibit noticeably longer pre-miRNA lengths than the other classes. (D) Analysis of human pre-miRNA lengths. Their overall properties are similar to mouse loci, including the subpopulation of long 5'-tailed mirtron hairpins and the substantially increased length of conventional mirtron pre-miRNAs as a class.

With this in mind, we were struck by the number of recently-annotated mouse and human mirtrons (this study and [13]) with pre-miRNA hairpins in excess of 100 nt. Fig 5A and 5B highlight cases of exceptionally long pre-miRNA hairpins in conventional and two-tailed mirtrons. Although the hairpin structure usually deteriorates distal to the miRNA/star duplex, we still observed specific small RNA duplexes that link genomically distant miRNA/star species. These loci add to the catalog of non-canonical loci in the upper echelon of mammalian pre-miRNA lengths, and suggested that splicing can generate a broader distribution of hairpin lengths than can Drosha.

We analyzed this systematically by comparing the length distributions of canonical pre-miRNAs and mirtrons. As expected, the distribution of Drosha-generated pre-miRNAs in mouse (Fig 5C) and human (Fig 5D) is tightly centered around 60 nt (mean±SD: 60±4.7 nt and 60±4.3 nt for human and mouse, respectively). Curiously, although we observe examples of atypically long pre-miRNAs in diverse mirtron biogenesis subtypes (e.g., Fig 5A and 5B), the dominant signal for long pre-miRNAs was contributed by conventional mirtrons. We do see that the distribution of 5'-tailed (mean±SD: 63±9.9 nt for human and 62±8.9 nt for mouse), 3'-tailed mirtron (mean±SD: 61±7.8 nt for human and 64±10.2 nt for mouse), and two-tailed mirtron (mean±SD: 69±24.4 nt for human and 64±3.7 nt for mouse) pre-miRNA hairpins is somewhat broader and more extreme, and includes members that extend into an exceptionally long range (>100 nt, Fig 5C and 5D). The length distribution of 5'-tailed mirtrons is statistically significant from canonical pre-miRNAs (p = 2.4E-7 and p = 8.5E-4 for human and mouse, respectively) since the set of canonical miRNAs is several fold larger, but lacks any extreme pre-miRNA lengths. Still, the average lengths of pre-miRNAs from tailed mirtrons are ~60 nt in both species.

By contrast, the lengths of conventional mirtron pre-miRNA were noticeably increased in both mouse (Fig 5C) and human (Fig 5D) datasets. Their average lengths were 80–85 nts, a range almost never broached by canonical miRNA hairpins. Moreover, their length distribution is much more heterogenous than any other miRNA classes (mean±SD: 83±9.7 nt and 87±18.5 nt for human and mouse, respectively). Comparison of conventional mirtron and canonical miRNA pre-miRNA are highly statistically significant (p = 1.1E-14 for human and p = 3.8E-11 for mouse). Therefore, the long-standing observation of a typical length restriction of canonical pre-miRNA hairpins is not a universal feature of miRNA substrates. Instead, we infer that the conventional mirtron class of Drosha-independent hairpins accesses greater structural freedom relative to other miRNA substrates, including other classes of splicing-derived miRNAs, whilst maintaining capacities for Dicer cleavage.

### Mirtrons exhibit greater terminal heterogeneities than canonical miRNAs

Confident annotation of miRNAs rests heavily on the terminal specificity and precision of the mature species, and stringent criteria are necessary to distinguish genuine miRNAs from amongst a sea of genomic hairpins whose incidental transcription and random degradation generate cloned short RNAs [3,30]. However, the recognition of abundant genomic substrates of mirtron resection pathways raises new complexity in defining the precision of “confident” miRNAs. In particular, the implied involvement of exonucleases in the biogenesis of tailed mirtrons may induce additional heterogeneity than is typical for canonical miRNAs.

**Fig 6:**
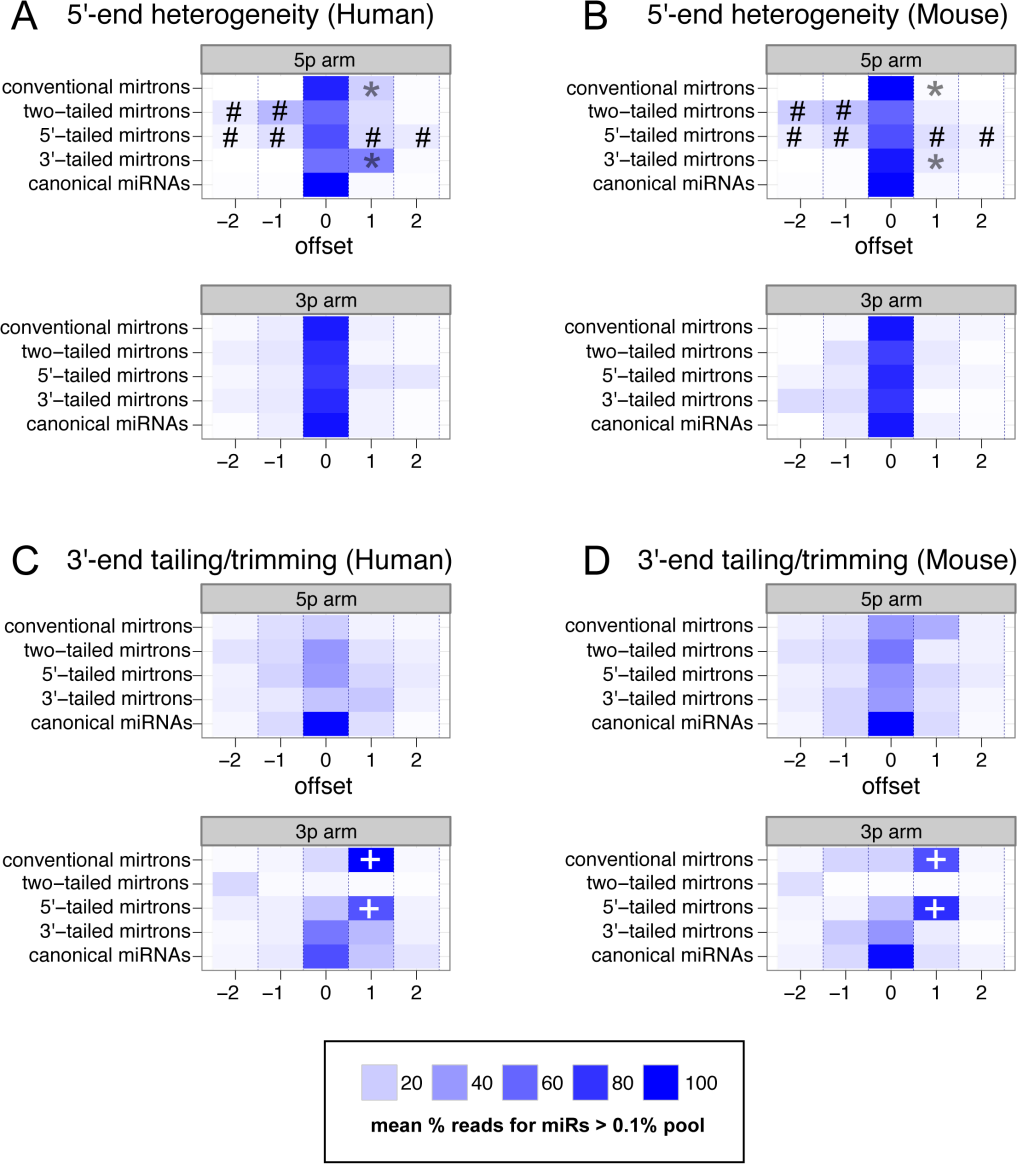
Distinct patterns of terminal heterogeneity in mirtron-derived small RNAs. (A, B) 5'-end heterogeneity in the 5p and 3p reads from human (A) and mouse (B) miRNA loci. There are several distinctions between canonical miRNAs and specific classes of mirtrons. These include substantial populations of mirtron-5p reads that lack their 5' nucleotide defined by splice donor sites, namely 5p reads from conventional mirtrons and 3' tailed mirtrons (*), and overall greater 5' heterogeneity in the 5' reads from 5' tailed mirtrons (#). (C, D) 3'-end heterogeneity in the 5p and 3p reads from human (C) and mouse (D) miRNA loci. Particularly notable are the dominant populations of 3'-tailed reads from 3p arms of conventional mirtrons and 5'-tailed mirtrons (marked by + signs), i.e., those reads that are defined by splice acceptor sites.

We analyzed terminal heterogeneities at the 5' ends and 3' ends of 5p-arm and 3p-arm reads from the three classes of mirtrons, and compared these to canonical miRNAs (Fig 6). We indeed observed that specific mirtron classes exhibit substantially increased terminal heterogeneity. For example, we observe that the 5' ends of 5p-arm reads of 3'-tailed mirtrons exhibited relatively diffuse start positions upstream and downstream of the dominant 5' end (Fig 6A and 6B, poundsigns), whereas conventional and 5' tailed mirtrons exhibited directional heterogeneity 1-nt downstream of the dominant 5' start of 5p-arm reads defined by the splice donor reference, particularly in human data (Fig 6A, asterisks). On the other hairpin side, we observed clear signatures in which the 3' termini of 3p-arm reads from conventional and 5'-tailed mirtrons were dominantly extended by one nucleotide from the splice acceptor reference (Fig 6C and 6D, plus-signs).

The 3' tailing of the 3p-arms of conventional and 5'-tailed mirtrons are mostly due to untemplated uridylation, and to a lesser extent adenylation (S7 Fig). We have described this phenomenon previously in invertebrate and vertebrate mirtrons [31], and our data here extend this more broadly. Indeed, simple inspection of many loci highlighted in main Figs show that mirtrons with 3p reads defined by splicing frequently accumulate high levels of untemplated uridylation and/or adenylation (e.g., Figs 1B and1D and 1E and 5 and 7A and 7B; see also Supplementary Websites for the full read patterns of all annotated mirtrons). Indeed, the presence of high levels of 3'-modified read is a characteristic feature that we can now use to positively evaluate novel mirtron annotations. However, as the 5' heterogeneity patterns of mirtrons had not been analyzed, we sought to characterize their bases more deeply.

**Fig 7:**
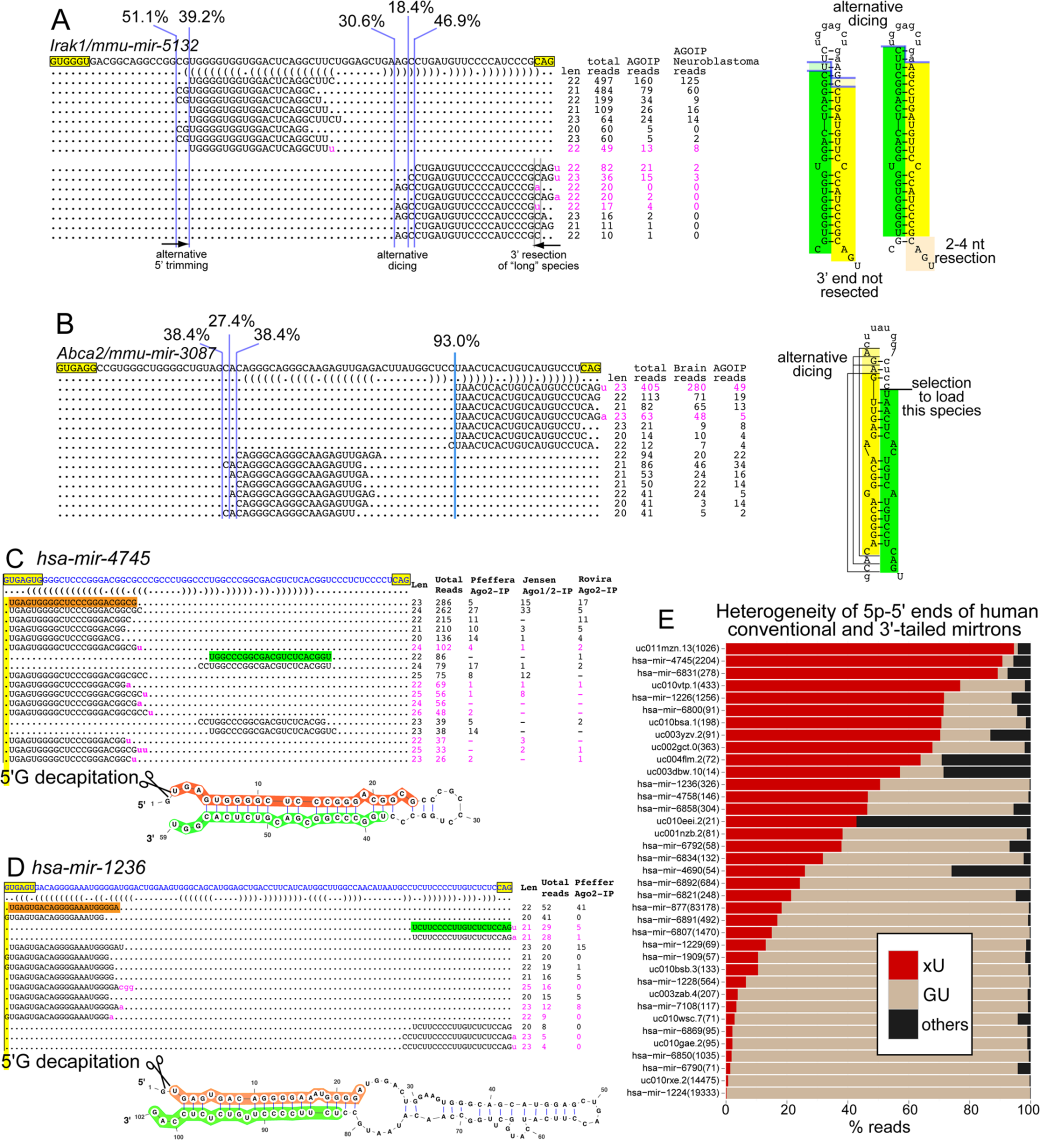
Unexpected patterns of 5' heterogeneity and processing of mirtrons. (A) A 5'-tailed mirtron in *Irak1* exhibits strong heterogeneity in its 5p species that differ in register by 2 nt; this is accompanied by strong heterogeneity in its 3p species. Inspection of this array of isomiR sequences suggests that distinct 5' ends of 5p species may instruct alternative Dicer cleavage. This may be accompanied by subsequent 3' resection of "long" 3p reads produced by Dicer cleavage closer to the terminal loop, and retention of 3'-uridylated 3p reads when the Dicer cleavage is further from the loop. (B) A counter-example in which broad 5' heterogeneity of 5p species, here distributed equally over three nucleotides, is not accompanied by 5’ heterogeneity of 3p species, which are extremely precisely-defined. All of these reads accumulate similarly in total and Ago-IP data. (C-E) Frequent 5' decapitation of select mirtron-5p reads defined by splicing. (C) Example of 3'-tailed mirtron (*hsa-mir-4745*) exhibiting nearly complete decapitation of 5'-G from its 5p reads (i.e., "xU" reads). These reads are present in multiple Ago-IP datasets. (D) Example of a conventional mirtron (*hsa-mir-1236*) exhibiting high frequency "xU" reads supported by Ago-IP evidence. (E) Summary of 5' heterogeneity amongst 5p reads from human conventional and 3'-tailed mirtrons, indicating that many mirtrons are subject to 5' decapitation.

### Tailed mirtrons can generate ordered 5' heterogeneity atypical for canonical miRNAs

Since the alteration of miRNA 5'-termini will redirect their targeting function, it is commonly thought that most "useful" miRNAs should not have heterogenous 5' ends. This is perhaps relevant to the abundant 5'-tailed mirtron pool. Although *Giardia* Dicer measures substrate cleavage from the 3' end [57], it was more recently shown that mammalian Dicer measures its cleavage position from the 5’ end of the hairpin [58]. Heterogeneous definition of the 5' ends of 5'-tailed mirtron hairpins may therefore induce corresponding heterogeneity in the 5' ends of their 3p species.

Fig 7 illustrates contrasting loci with respective 5'-end heterogeneity. A dramatic example of alternative 5'-termini is provided with mouse *mir-5132*, located in *Irak1* (Fig 7A). It exhibits alternative 5' ends of both 5p and 3p species, differing in registers over 3–4 nt each, an isomiR range that is very unusual for canonical miRNAs. Because the 3' end of the mirtron hairpin is fixed, in contrast to alternative Drosha cleavage which coordinately affects both sides of the hairpin, we infer that the degree to which the 5' end of the pre-miRNA hairpin exonucleolytically trimmed will create different small RNA duplexes with different outcomes. The 5p “CGUGG…” terminus likely generates a hairpin that can undergo some alternative dicing to yield alternative 5' ends of corresponding 3p reads, and these are dominantly uridylated. However, further 5' trimming to generate the 5p "UGG…" terminus may be expected to shift the Dicer cleavage site so as to generate a 25-nt uridylated species. Since this is beyond the typical length of Ago cargoes, this seems to be associated with 3' trimming of 3p reads generated from the strongly alternative dicing reaction, as observed in Ago-IP data (Fig 7A).

Many 5'-tailed mirtrons exhibit increased 5' heterogeneity of reads from both hairpin arms (Fig 6A and 6B). However, this is not universally the case. For example, a mirtron in mouse *Abca2* (*mir-3087*) exhibits extremely heterogeneous 5' ends of its 5p species, with three different starts being represented equally. However, the vast majority of 3p reads are defined precisely. The disparate behavior of 5' terminal precision of 5p and 3p reads are represented in Ago-IP data (Fig 7B). Such behavior suggests that strategies for strand selection are more complex than currently envisioned. Overall, it appears that definition of 5' termini of miRNA-5p species by a nuclease other than Drosha (i.e., with 5' tailed mirtrons) can influence the register of Dicer cleavage in ways that are not currently predictable. Moreover, the processing of many mirtrons lacks 5' precision, which is usually taken as a hallmark for genuine miRNAs, even though such mirtrons still exhibit clear evidence for dicing and are frequently captured in Ago-IP data. This data indicate the complexity and challenges of annotating Dicer-substrate miRNAs.

### High frequency decapitation of 5'-G on select mirtron-5p reads

Unlike the 5' termini of 5p species from 5'-tailed mirtrons, the 5p species from conventional mirtrons and 3'-tailed mirtrons are directly defined by splicing. Therefore, a base expectation is for them to initiate relatively homogenously with the 5' splice donor sequence "GU". While this is the case for many loci, we unexpectedly observed several conventional mirtrons and 3'-tailed mirtrons whose dominant 5p species did not initiate with 5' GU. Instead, these loci exhibited frequent loss of 5'-G of 5p reads, which we refer to as "xU" reads. Some prominent cases with the highest frequency of "xU" species had hairpins with short 5' overhangs. For example, *hsa-mir-4745* generates >90% "xU" reads and its pre-miRNA exhibits 4:3 nt (5':3') overhangs (Fig 7C). In essence, loss of 5'-G might be interpreted as removal of a single-nt 5' tail. However, loci such as *hsa-mir-1236* exhibit a typical pre-miRNAs with a 3' overhang, but still generate high frequency "xU" reads (Fig 7D).

We sought the breadth of this phenomenon by plotting the "xU" frequency of all splice donor-derived mirtron-5p loci. Considering loci with ≥10 total 5p reads, we observed that 27/37 human and 11/31 mouse mirtron-5p loci that accumulated at least 10% 5' G-decapitated species (Fig 7E and S8 Fig). Moreover, there were 12 human and 2 mouse 5p mirtrons for which xU reads were the majority of cloned species. Such 5'-directed trimming converts mirtron-5p species from having the least-favored (5'-G) to the most-preferred (5'-U) nucleotide for binding to the Ago2 MID domain [51], and we confirm the accumulation of abundant "xU" species in Ago-IP datasets (e.g. Fig 7C and 7D).

Conceivably, these phenomenon may be related to the fact that mammals exhibit seemingly robust pathways for 5' mirtron tail removal, as suggested by the >800 such loci (Fig 2A). Notably, the exonucleotic removal of single 5' nucleotides from the 5p species of canonical pre-miRNA hairpins would not be distinguishable from alternative Drosha processing. However, this process can utilize conventional pre-miRNA structures (Fig 7D). Overall, the observation of substantial 5' decapitation of nucleotides from the 5' end suggests a mechanism to alter seed identity via an unidentified enzyme.

## Discussion

### A greatly expanded annotation of mammalian mirtrons

In spite of a still-growing appreciation of non-canonical miRNA biogenesis pathways [6], the bulk of miRNA reads in most cell types are generated from canonical loci. In fact, the majority of miRNA reads in individual celltypes can often be accounted for by a dozen or so miRNAs, and sometimes fewer [59]. On the other hand, our recent [13] and current efforts to annotate mirtrons in mouse and human genomes yield the unexpected conclusion that a substantial fraction of confident miRNA loci in mammals are actually non-canonical. As with our efforts with canonical miRNAs, we utilized strict criteria to evaluate small RNA evidence for specific dicing of precursor hairpins, and the vast majority of mirtrons generate reads present in Ago complexes (S2 and S3 Tables).

The regulatory impact of small RNAs is concentration-dependent [60,61]. As the bulk of mirtrons generate small RNAs that accumulate modestly, it is unclear what impact individual mirtrons have on endogenous gene expression. In fact, the massive turnover of mirtrons between mouse and human (Fig 2B) indicates they only infrequently incorporated into conserved regulatory programs. Despite this, mirtrons do generate miRNA-class molecules and generally incorporate into Ago complexes (Fig 2C–2F).

One way for mirtron-derived small RNAs to gain enhanced impact is to "piggyback" onto existing conserved miRNA target networks that are already selected for function. Such a strategy was proposed for certain mammalian non-canonical miRNAs [62], and we observe that the *Drosophila* 3'-tailed mirtron *mir-1017* has converged onto the pre-existing seed of the canonical Brd box family [10]. Although mouse and human mirtrons do not seem to be enriched for seed similarity to conserved mammalian miRNAs, relative to other portions of their mature sequences, we do observe a set of potential mirtron "seed mimics". We summarize information on these relationships in S6 Table, which may serve to prioritize a subset of mirtrons for functional studies.

### Non-canonical miRNAs reveal novel complexities of Dicer substrate processing

Mirtrons are advantageous for characterizing post-primary processing of miRNA species, because alterations to hairpin termini that are generated by splicing can be easily distinguished [31]. In general, terminal modifications of canonical miRNAs can be confidently inferred only when the resultant reads fail to match the genome (e.g. with additions of untemplated nucleotides). Otherwise, 5' trimming, 3' trimming and 3' tailing events that fortuitously match the genome are difficult to distinguish from alternative RNase III processing events. In contrast, such modifications to mirtrons can be categorized as reads that lack 5'-GU or AG-3' nucleotides at splicing-derived termini.

In this study, we found that mirtron-5p species initially defined by splice donor sequences can exhibit high frequency 5' trimming of guanine residues. In essence, "xU" reads may represent removal of a single 5' nucleotide tail. This modification may increase the capacity for loading mirtron-5p reads, since it alters their 5' ends from being disfavored (5'-G) to optimal (5'-U) binding to the Ago MID domain [51]. Such trimming is uniquely noticeable with mirtrons whose 5' pre-miRNA hairpin end is defined directly by splicing, and would not be distinguished from alternate Drosha cleavage of canonical pre-miRNAs. Since 5' trimming of even a single nucleotide is expected to induce a radical shift in miRNA target networks, it will be interesting to know if such post-primary processing events occur in a "hidden" fashion with any canonical pre-miRNAs.

On the other hairpin end, close inspection of mirtron-derived read patterns raises questions regarding the strategy for selection of cleavage position(s) by Dicer. An initial model from *Giardia* studies posited that the PAZ domain allows Dicer to measures substrate cleavage from the 3' end [57]. However, it was more recently shown that mammalian Dicer contains a 5' phosphate-binding pocket, which allows it to measure its cleavage position from the 5' end of the hairpin [58]. In vitro experiments showed that variation of pre-miRNA 3' overhang length by several nts did not adjust the register of Dicer cleavage. Consistent with this, we observe that abundant 3' untemplated uridylation is often associated with a common dominant 5' end of mirtron-3p species, suggesting that that this modification may not adjust Dicer cleavage.

Reciprocally, we also observe many 5'-tailed mirtron hairpins that exhibit variable 5' ends of 5p species, presumably due to exoribonucleolytic trimming that is less precise than Drosha cleavage. While we find many cases in which alternative 5' hairpin ends likely induce alternative Dicer cleavages, in keeping with a 5' measuring rule, we also find cases in which highly variable 5' hairpin ends are associated with precise definition of partner 3p species. These observations hint at further complexity in defining the positions of Dicer cleavage.

Overall, our deep annotation of mammalian mirtrons reveals multiple aspects of small RNA processing that are not apparent with the general study of canonical miRNAs, and delineates informative sets of test substrates with which to examine these further.

### Dynamics of mirtron evolution

Although some mirtrons are well-conserved as with canonical miRNAs [13,42], these are clearly the exception. We previously documented increased evolutionary flux of mirtrons relative to canonical miRNAs across *Drosophila* species [30]. This notion is bolstered by our current appreciation that of ~500 mouse mirtrons and ~500 human mirtrons that we annotated according to stringent criteria, only a few percent are shared between these species. Indeed, the paucity of conserved mirtrons may suggest they are actively prevented from incorporation into conserved regulatory networks.

We speculate that the Drosha/DGCR8 complex effectively filters a subpopulation of hairpin substrates for further processing by Dicer, which is more indiscriminate in substrate selection. On the other hand, the splicing pathway may fortuitously generate a diversity of "dice-able" hairpins. In support of this scenario, we showed the structural properties and small RNAs patterns from mirtrons are collectively much more heterogeneous than canonical miRNAs, and in some cases, embody characteristics that would normally make their annotation as miRNAs somewhat suspect. If we consider that mirtrons are generally adventitious substrates that access Dicer, then this could have facilitated the emergence of mechanisms that generally suppress their biogenesis. Conceivably, this may be related to the high rates of mirtron uridyation [31]. We observe that uridylation of mirtron-3p termini defined by splicing is a pervasive feature in mammalian mirtrons (Fig 6 and S7 Fig), which indeed mostly fall into the 5' tailed or conventional classes (Fig 2A). Since hairpin uridyation has been linked to the turnover of certain pre-miRNAs [63], it is conceivable that mirtron tailing is linked to their differential evolutionary lability. Evidence for such a mechanism has recently been presented in the *Drosophila* system [64,65], and this scenario merits investigation in mammals.

## Materials and Methods

### Processing and curation of mouse and human small RNA data

We collected 690 human and 348 mouse small RNA data sets from various tissues/cell lines from NCBI GEO/SRA. Among them, 189 human and 125 mouse new small RNA data sets were added since our previous meta-analysis [13]. The accession numbers of all data sets analyzed in this study are summarized in S1 Table. After removing 3’ adaptor sequences, we mapped human and mouse small RNA reads to human (hg19) and mouse (mm9) genome assemblies, respectively. Unmapped reads were iteratively trimmed one nucleotide each iteration retaining a read length of ≥17nt, and then mapped to the genome using Bowtie with no mismatches, up to 4 iterations.

### Mirtron annotation

We obtained intron annotations from UCSC Genome Browser via table browser and used the “knownCanonical” table including the canonical splice variant of genes. We re-annotated mirtrons using our annotation pipeline for small RNA mapping to intron termini and analysis of hairpin and duplex properties [13], with modifications to increase read depth stringency. In the main pipeline, we required at least 50 total duplex reads (mature and star strand reads), with at least 5 star strand reads from which 3' duplex overhang characteristics were assessed. Because we included a number of Ago-IP libraries in this analysis, we also considered loci with <5 star strand reads to be confident if there were at least 100 reads from the mature arm, at least 20 of which were recovered from Ago-IP datasets. However, only a relatively small number of loci necessitated this second criterion (see S2 and S3 Tables). As we analyzed greater small RNA library variety and read depth than before [13], we were able to identify novel 242 human and 248 mouse mirtrons even though our criteria were more stringent. All of the loci were manually vetted to ensure confident inference that their progenitor hairpins were both generated by splicing, and subject to dicing to yield the mapped small RNAs.

There are 9 loci that failed these criteria, but we believe should be recorded as confident mirtron loci (listed in S2 and S3 Tables). 5 loci had ≤ 3 star reads but ≥ 20 reads were recovered in Ago-IP libraries (human: *uc002okf*.*4*, *uc009vxw*.*2*, *uc003bmk*.*3*, *uc001uej*.*1*; mouse: *uc007nzl*.*1*), and 4 loci had 3–4 star reads but ≥10 Ago-IP reads and ≥100 total reads (human: *uc001bvb*.*1*, *uc003yzv*.*2*, *uc002ojz*.*2*; mouse: *uc008hbc*.*17*). We also downgraded 4 mirtrons in mirBase which failed the 50 total reads criteria (*hsa-mir-1178*, *hsa-mir-7107*, *hsa-mir-1238*), or lacked both star reads and Ago-IP reads (*hsa-mir-4641*). Finally, we note that while the reads assigned to the vast majority of mirtron loci map uniquely, in rare cases some reads were mapped to multiple locations. The quality of dicing features amongst the aggregate reads had to be satisfactory at each genomic locus considered as a mirtron. Detailed information on the mapped reads, their modified reads, the libraries of origin, and other genomic information on all the mirtrons annotated in this study can be browsed at these web links: http://compgen.cshl.edu/mirna/mam_mirtrons/hg19_candidate/ (human) and http://compgen.cshl.edu/mirna/mam_mirtrons/mm9_candidate/ (mouse).

### Analysis of 3' untemplated additions

Mirtron hairpin boundaries were defined on the basis of intron splice donor (GT) or acceptor (AG) sequences. The derivation of mirtron-3p reads from splicing permitted us to utilize the "AG" splice acceptor as an external reference for the primary-processed species. Thus, reads that extended past the "AG" likely bear untemplated nucleotides, and were called as such regardless of whether they match the genome or not. The boundaries of canonical pre-miRNA hairpins were based on miRBase v19 with sequences defined by the most abundant matching read from all aggregated small RNA libraries analyzed in this study.

### Correlation of mirtron and host mRNA expression across tissues

We also obtained mRNA-seq data for human (body map II; SRA accession: ERP000546) and mouse (SRA accession: SRP016501) across different tissues. We mapped these mRNA-seq data using TopHat to corresponding genomes hg19 and mm9, respectively. For each mirtron:host pair an expression value was calculated for both the mirtron and the host gene. The mirtron expression level was quantified as a proportion of small RNA reads per million mapped miRNA reads. Since mRNAs are subject to various types of alternative processing, which could potentially affect the observed correlation levels, the host gene expression was estimated by multiple measures to enable as robust assessments as possible. Host gene expression was alternately estimated at the (1) gene-level (RPKM, union of all gene exons), (2) mirtron-flanking exons (RPKM), and (3) mirtron spanning spliced reads (RPM). While this final category provides the most precise quantification of host intron usage, junction spanning reads are considerably more rare, motivating the use of gene and flanking exon level estimates.

For each mirtron:host mRNA pair the Pearson correlation was calculated between expression levels in matched samples (human: [brain, breast, heart, kidney, liver, white blood cells], mouse: [brain, heart, kidney, liver, lung, skeletal muscle, spleen]). The distribution of observed correlation values was represented using histogram and cumulative distribution plots. An alpha = 0.99 empirical confidence envelope (the gray shaded area on the cumulative plot) was generated by repeating the analysis 100 times with shuffled tissue labels. A two-tailed 1-sample Wilcoxon test was used to assess the significance of the observed deviation of the distribution median from zero.

### Additional computational analysis and statistical tests

#### Mirtron conservation

For mirtrons, both sequence and positional conservation between human and mouse were searched: the sequence conservation required a conserved 7-mer seed region while the positional conservation required mirtrons hosted by homologous introns only. The alignments of both sequence and positionally conserved mirtrons were made using the UCSC human/hg19 46-way multiple alignment (S2 Fig). We removed species where sequences with more than 30% indels relative to the human reference sequence.

#### Sampling of a length-matched distribution of control introns to mirtron hairpins

As comparisons of MFEs between mirtron-hosting introns and other bulk introns are sensitive to the length of the sequences analyzed, a subset of the bulk intron set with sequence lengths matched to the mirtron set was sampled to have the same length distribution by “matchControls” function in R package e1071 [66]. t-test was used to measure statistical significance of mean MFE/base difference between mirtron hosting introns and other introns.

#### Intron numbers in host genes

Number of introns for mirtron-hosting genes was compared to that for other types of small RNA hosting genes including intronic conserved/non-conserved miRNAs, snoRNAs, tRNAs, as well as compared to that for other genes. In Fig 2, the trimmed mean (trimming off the top 2% genes) was calculated to remove the outlier genes with extremely high number of introns for each set. For example, 10 genes with ≥ 56 introns and 7 genes with ≥ 88 introns were excluded from human and mouse 5’-tailed-mirtron set, respectively. Wilcoxon rank-sum test was used to measure statistical significance of mean number of introns.

#### Analysis of mirtron enrichment in Ago-IP data

We utilized dataset GSE45506 comprising endogenous Ago1-4, Control-IP (RmC-IP) and Input (input RNA before IP) from human HeLaS3 cells [43]; note that Ago4 is not expressed in these cells. Among 478 annotated human mirtrons in this study and 1465 canonical miRNAs from miRBase, 138 mirtrons and 755 canonical miRNAs had zero reads in all of these libraries and were excluded from analysis. We also analyzed dataset SRP014535 comprising Ago1-IP, Ago2-IP and IgG-IP from mouse CD4+ T cells [33]. The replicated samples were combined. Among 488 annotated mouse mirtrons in this study and 852 cannonical miRNAs from miRBase, 277 mirtrons and 284 canonical miRNAs did not have any reads in any of these samples were excluded from the analyses. We analyzed all other loci with at least one read in at least one mouse (212 mirtrons and 568 canonical miRNAs) or human dataset (343 mirtrons and 710 canonical miRNAs). To enable plotting on log scales, we included an offset of 0.1 RPM for any locus that lacked reads in any corresponding library (e.g., as was the case for many loci in control IP datasets). Cumulative expression distributions of mirtrons and canonical miRNAs for each sample were plotted. Expression units: log2(x+0.1) for x RPM. Statistical significance was by t-test with adjusted p-values by Holm method. A summary of fraction of mirtrons and canonical miRNAs expressed at varying read depths are included in S4 and S5 Tables.

## Supporting Information

S1 Fig Breakdown of mouse/human mirtron overlaps by biogenesis subtype class. Note that even these are all orthologous mouse-human introns that harbor splicing-derived miRNAs, in many cases, the mature small RNAs are substantially diverged and/or the mirtron subtype has shifted.
(PDF)

S2 Fig Alignments of mouse/human orthologous mirtrons. Shown are introns that generated small RNA duplexes in both mouse and human (highlighted), with their respective alignments across the available vertebrate genomes. Positions of divergence, with respect to the reference genome at top, are shaded in red. Note that there is not evidence for small RNA generation across most of the aligned species, and only in a small number of the species exhibit a classic "saddle-shaped" evolutionary profile in which the hairpin loop clearly evolves more quickly than does the hairpin arms (e.g. as is seen for *mir-1224*, *mir-3064*, and *mir-877*).
(PDF)

S3 Fig Distribution of canonical miRNAs and mirtrons across sRNA-seq samples. (A, B) Plot of the maximum number of reads that were mapped to mouse and human mirtrons across individual libraries (x-axis: maximum #reads in individual libraries; y-axis: #mirtrons). (C, D) Plot of the maximum number of reads that were mapped to mouse and human mirtrons across grouped sets of similar tissues or cell lines (x-axis: maximum #reads in different tissues/cell lines; y-axis: #mirtrons). (E, F) Expressed mirtron distribution across sRNA-seq libraries, in comparison to canonical miRNAs. Considering mirtrons with greater than 5 RPM and canonical miRNAs with greater than 20 RPM in a single sample as expressed miRNAs, we observed that a majority of mirtrons expressed in 2–5 samples, indicating mirtrons tend to be specifically expressed. By contrast, canonical miRNAs have a different distribution with a peak of miRNAs expressed in > = 100 samples in both human and mouse, and a peak of 2–5 samples in mouse and of 11–20 samples in human, consistent with both broadly expressed and specifically expressed canonical miRNAs. Other cutoffs show a similar trend.
(PDF)

S4 Fig Correlation of mirtron and host gene across tissues in human and mouse. We compared the expression of mirtrons and host mRNAs across various human and mouse tissues. For 399 mouse mirtrons small RNA and mRNA were compared from brain, heart, kidney, liver, lung, skeletal muscle, and spleen samples. For 223 human mirtrons, the tissues used were brain, breast, heart, kidney, liver, white blood cells. We utilized two strategies for assessing mRNA expression, by quantifying the union of all gene exons (A, C), as well as a more restricted analysis using only the reads that specifically span the mirtron-containing intron (B, D). The expression of each mirtron in each tissue was quantified as the number of reads per million mapped miRNA reads. We calculated the Pearson correlation coefficient for each mirtron:mRNA pair across tissues. We plotted their distribution as a cumulative distribution (upper row), and contrasted this with 100 permutations in which the analysis is repeated with shuffled tissue-labels. In the lower row the observed distribution of correlation coefficients is represented as a histogram. A two-tailed 1-sample Wilcoxon test was used to assess the significance of the observed deviation of the distribution median from zero. In all comparisons there is a statistically significant shift of the median of (mirtron:host) correlation towards positive correlation values. In the mouse data, the shift is less pronounced when using all host gene exons (A) than when using only the spliced mRNA reads that traverse the mirtron-containing intron (B), likely reflecting the latter is a more specific comparison. This trend was not observed in human, likely due to undersampling of these highly specific spliced reads (the mouse data are about an order of magnitude deeper than the human mRNA-seq data). This can be observed in the individual gene examples provided in S5 Fig.
(PDF)

S5 Fig Examples of mirtron: Host mRNA correlation across tissues. Four rows of data are shown for each locus: (1) mirtron- miRNA reads (in reads per million mapped miRNA reads, RPMM), (2) spliced mRNA-seq reads that directly traverse the mirtron-containing intron (in reads per million mapped, RPM), (3) mRNA-seq reads in the two flanking exons upstream and downstream of the mirtron-containing intron (in reads per kilobase of transcript per million mapped reads, RPKM), and (4) mRNA-seq reads in the entire mirtron host gene model (in RPKM). (A) Mouse data. (B) Human data. For both species, examples of well-correlated as well as discordant mirtron and host mRNA expression patterns are shown. Note that the available human mRNA-seq data were nearly an order of magnitude smaller than the those in mouse; thus, the measurements of spliced reads across mirtron-containing introns are adversely affected in some cases. While the overall patterns are similar amongst most of the mRNA measurements, no spliced reads were recovered for *WA5B2(hsa-mir-1224)*, indicating undersampling. As well, the pattern of spliced *TBC1D24(uc002cqk*.*5)* reads is discordant with other measures of mRNA expression, and could represent an undersampling artifact.
(PDF)

S6 Fig Sequence and length properties of mirtron-containing introns in mouse. (A) Comparison of mirtron-bearing introns with total introns in mouse. The distribution of total intron lengths is much broader than for mintrons. The dominant class of 5' tailed mirtrons derives mostly from introns that are <3kb in length, while the 3'-tailed mirtrons and conventional mirtrons derive from very short introns. (B, C) Nucleotide bias of small RNAs from 5'-tailed mirtrons. (B) Biased nucleotide identities of mirtron-5p reads from the dominant class of 5'-tailed mirtrons. Compared to an equivalent sequence range of control introns of similar length, mirtron-5p reads exhibit substantial 5'-U bias and overall enrichment of G across their lengths. The G bias is greater in the 5' than 3' regions of the mirtron-5p reads, and is not evident in bulk intron sequences downstream of their ~22 nt lengths. (C) Biased nucleotide identities of mirtron-3p reads from the dominant class of 5'-tailed mirtrons. Compared to control introns, there is substantial 5'-U bias (evident with aligning by their 5' ends) and substantial C-bias across their length. Note that the bulk introns exhibit polypyrimidine tracts upstream of the splice acceptor site (YAG), but mirtrons exhibit greater representation of C while control introns show greater bias for U. (D) Mirtronic regions exhibit much lower minimum free energy (MFE) than control intronic regions. CDF (cumulative distribution function) is plotted for MFE/base distribution. (E) All four classes of mirtrons are hosted by genes with greater numbers of introns than average genes. Various classes of other intronic non-coding RNAs (e.g. tRNAs, snoRNAs, and either conserved or non-conserved canonical miRNAs) typically reside in genes with larger numbers of introns than bulk genes, but their averages are intermediate to all classes of mirtrons. (F) Bar graphs that emphasize the individual properties of genes that host various classes of non-coding RNAs. It is evident that the all four classes of mirtrons have a broader distribution of intron numbers relative to other types of non-coding RNAs.
(PDF)

S7 Fig 3' untemplated additions in mammalian canonical miRNA-3p and mirtron-3p species. Pie charts depicting the fractions of 3p-arm canonical miRNAs and mirtron-derived miRNAs that match the genome directly, or following trimming of 3' untemplated nucleotides. For both 5'-tailed and conventional mirtrons, reads ending precisely at the AG splice acceptor and reads extending beyond the AG splice acceptor were pooled from all loci. For canonical miRNAs, the reads ending precisely at and extending beyond the annotated 3p boundaries, which was defined by the most abundant matching read from all aggregated small RNA libraries analyzed in this study, were pooled from all loci. The pie charts illustrate categories of untemplated nucleotide additions with >1% of total reads; the remaining reads were classified as “others”. The vast majority of human and mouse canonical miRNA-3p reads match the genome. In contrast, the majority of mirtron-3p species bear untemplated additions, primarily uridylation and to a lesser extent adenylation.
(PDF)

S8 Fig Summary of 5' heterogeneity amongst 5p reads from mouse conventional and 3'-tailed mirtrons. Since these are generated directly by splicing, such 5p reads should bear the 5'-GU splice donor sequence. We tally the frequency of unmodified GU reads, "guanine-decapitated" reads ("xU"), and other reads in this graph. Many mirtron hairpins are subject to substantial 5' decapitation.
(PDF)

S1 Table Small RNA datasets analyzed in this study. (XLS)

S2 Table Mouse mirtron loci annotated and analyzed in this study. (XLS)

S3 Table Human mirtron loci annotated and analyzed in this study. (XLS)

S4 Table Ago-IP enrichment analysis of human canonical miRNAs and mirtrons. (XLS)

S5 Table Ago-IP enrichment analysis of mouse canonical miRNAs and mirtrons. (XLS)

S6 Table Mirtrons with at least 6-mer seed matching to conserved mammalian miRNAs. (XLS)
