# Supplementary material for: Analysis of Nearly One Thousand Mammalian Mirtrons Reveals Novel Features of Dicer Substrates
Source: PLoS Comput Biol. 2015 Sep 1;11(9):e1004441. doi: 10.1371/journal.pcbi.1004441 (PMC4556696; doi:10.1371/journal.pcbi.1004441)
Supplement: S1 Fig — Note that even these are all orthologous mouse-human introns that harbor splicing-derived miRNAs, in many cases, the mature small RNAs are substantially diverged and/or the mirtron subtype has shifted. (PDF) [file pcbi.1004441.s001.pdf]

Human-mouse orthologous introns that contain mirtrons

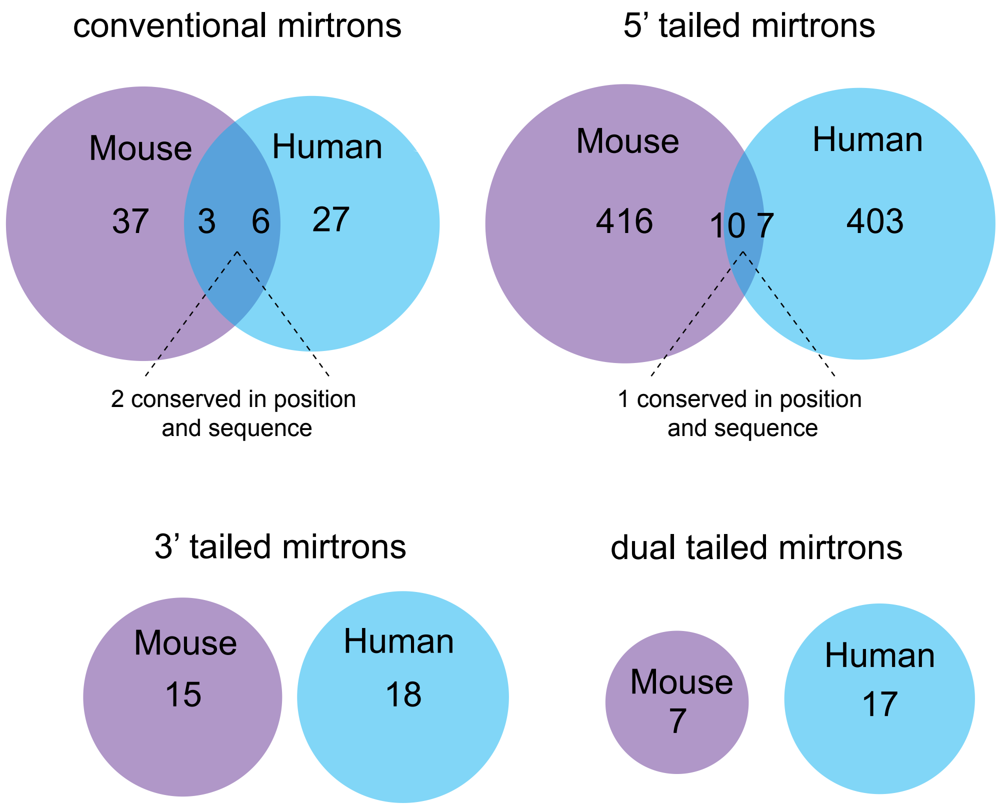

**Mouse**		**Human**	
3 conserved in both position and sequence:			
mmu-mir-1224	conventional mirtron	hsa-mir-1224	conventional mirtron
mmu-mir-3064	5p-tailed mirtron	hsa-mir-3064	5p-tailed mirtron
mmu-mir-877	conventional mirtron	hsa-mir-877	conventional mirtron
10 conserved in position only:			
mmu-mir-5134	5p-tailed mirtron	hsa-mir-6767	5p-tailed mirtron
mmu-mir-6921	5p-tailed mirtron	hsa-mir-6777	5p-tailed mirtron
mmu-mir-6956	5p-tailed mirtron	uc003atr.19	conventional mirtron
mmu-mir-6988	5p-tailed mirtron	hsa-mir-6751	5p-tailed mirtron
uc007iry.12	5p-tailed mirtron	hsa-mir-1229	conventional mirtron
uc008jba.8	5p-tailed mirtron	uc004bvw.9	conventional mirtron
uc008jev.23	5p-tailed mirtron	uc011maw.1	conventional mirtron
uc008kvn.5	5p-tailed mirtron	hsa-mir-6745	5p-tailed mirtron
uc008wwz.19	conventional mirtron	uc002rji.2	5p-tailed mirtron
uc009met.10	5p-tailed mirtron	uc002nhg.3	5p-tailed mirtron

Note: Even though these are all orthologous mouse-human introns that harbor splicing-derived miRNAs, in many cases, the mature small RNAs are substantially diverged and/or the mirtron subtype has shifted.

Wen and Ladewig
Supplementary Figure 1
