# Supplementary material for: Analysis of Nearly One Thousand Mammalian Mirtrons Reveals Novel Features of Dicer Substrates
Source: PLoS Comput Biol. 2015 Sep 1;11(9):e1004441. doi: 10.1371/journal.pcbi.1004441 (PMC4556696; doi:10.1371/journal.pcbi.1004441)
Supplement: S2 Fig — Shown are introns that generated small RNA duplexes in both mouse and human (highlighted), with their respective alignments across the available vertebrate genomes. Positions of divergence, with respect to the reference genome at top, are shaded in red. Note that there is not evidence for small RNA generation across most of the aligned species, and only in a small number of the species exhibit a classic "saddle-shaped" evolutionary profile in which the hairpin loop clearly evolves more quickly than does the hairpin arms (e.g. as is seen for mir-1224, mir-3064, and mir-877). (PDF) [file pcbi.1004441.s002.pdf]

Wen and Ladewig
Supplementary Figure 2

Alignments of mouse/human orthologous mirtrons. Shown are introns that generated small RNA duplexes in both mouse and human (highlighted), with their respective alignments across the available vertebrate genomes. Positions of divergence, with respect to the reference genome at top, are shaded in red. Note that there is not evidence for small RNA generation across most of the aligned species, and only in a small number of the species exhibit a classic "saddle-shaped" evolutionary profile in which the hairpin loop clearly evolves more quickly than does the hairpin arms (e.g. as is seen for *mir-1224*, *mir-3064*, and *mir-877*).

hsa-mir-1224

```
Human          GUGAGGACUCGG---GAGGUGGAGGGUGGU--GCCGCCGG---GGCCGGG-CG---CUGUUUCAGCUCGCUU-CUCCCCCCA------CCUCCUCUCUCCUCAG
Chimp          GUGAGGACUCGG---GAGGUGGAGGGUGGU--GCCGCCGG---GGCCGGG-CG---CUGUUUCAGCUCGCUU-CUCCCCCCA------CCUCCUCUCUCCUCAG
Gorilla        GUGAGGACUCGG---GAGGUGGAGGGUGGU--GCCGCCGG---GGCCGGG-CG---CUGUUUCAGCUCGCUU-CUCCCCCCA------CCUCCUCUCUCCUCAG
Orangutan      GUGAGGACUCGG---GAGGUGGAGGGUGGC--GCCGCCGG---GGCCGGG-CG---CUGUCUCAGCUCGCUU-CUCCCCCCA------CCUCCUCUCUCCUCAG
Rhesus         GUGAGGACUCGG---GAGGUGGAGGGUGGC--GCCGCCGG---GGCCAGG-CG---CUGUCUAAGCUCGCUU-CUCCCCCCA------CCUCCUCUCUCCUCAG
Baboon         GUGAGGACUCGG---GAGGUGGAGGGUGGC--GCCGCCGG---GGCCAGG-CG---CUGUCUAAGCUCGCUU-CUCCCCCCA------CCUCCUCUCUCCUCAG
Marmoset       GUGAGGACUCGG---GAGGUGGAGGGUGGC--GCCGCCCG---G--CGGG--G---CUGUCUCAGCAUGCUU-CUCCCCCCA------CCUCCUCUCUCCUCAG
Mouse_lemur    GUGAGGACUCGG---GAGGUGGAGGGUGGC--AUCGUCAG---GGCCUGGACG---CUGUCUUAGCUCGCUU-CUCCCCCCA------CCUCCUCUCUCCUCAG
TreeShrew      GUGAGGACUCGG---GAGGUGGAGGGCGGC--GUCGCCAG---GGCCGGG-CG---CCGUCUCAGCCCGC-U-CGUCCCCCA------CCUCCUCUCUCCUCAG
Mouse          GUGAGGACUGGG---GAGGUGGAGGGUAGC--AUCAUUAG---AGCCAGAGCU---CUGUCUCAGCUCCCU--CUCCCCCCA------CCUCUUCUCUCCUCAG
Rat            GUGAGGACUGGG---GAGGUGGAGGGUAGU--ACCAUUAG---AGCCAGAGCU---CUGUCUCAGCUCCCU--CUCCCCCCA------CCUCUUCUCUCCUCAG
Kangaroo_rat   GUGAGGACUCGG---GAGGUGGAGGGUGGC--GUUGCCAG---GGCCAGGGUG---CCAUCUCAGCUCGCUUCUCCCCCCCA------CCUCCUCUCUCCUCAG
Guinea_Pig     GUGAGGACUCGG---GAGGUGGAGGGAGGCCGGUCGCC-G---GGCCUGGGCUGGCGUGUCCAGUUUGCCU--GUCCCCCCA------CCUCCUCUCUCCUCAG
Squirrel       GUGAGGACUCGG---GAGGUGGAGGGUAGU--GUCACCAG---AGCUAGAGUG---AUGUCUCAGCUCACUU-CUCCCCCCA------CCUCCUCUCUCCUCAG
Rabbit         GUGAGGACUCGG---GAGGUGGAGGGCGGC--GUCGCCAG---GGCCAGAGCG---CUGUCUCAGCUCGCUU-CUCCCCCCA------CCUCCUCUCUCCUCAG
Pika           GUGAGGACUCGG---GAGGUGGAGGGUAUC--AUUGCCAG---AGCCGGGAUG---CUGUCUCAACUCGCUU-CUCCCCCCA------CCUCCUCUCUCCUCAG
Alpaca         GUGAGGACUCGG---GAGGUGGAGGGUGGC--GUGGCCAG---GGCCAGGGCA---CUGUCUCAGCUCGUUU-CUCCCCCCA------CCUCCUCUCUCCUCAG
Dolphin        GUGAGGACUCGG---GAGGUGGAGGGUGGC--ACUGCCAG---GGCCAGGGCG---CUGUCUCAGCUCGAUU-CUCCCCCCA------CCUCCUCUCUCCUCAG
Cow            GUGAGGACUCGG---GAGGUGGAGGGUGGC--GCUGCCAG---GGCCAGGGCA---CUGUCUCAGCUCGCUU-CUCCCCCCA------CCUCCUCUCUCCUCAG
Horse          GUGAGGACUCGG---GAGGUGGAGGGUGGC--ACUGCCAG---GGCCAGGGCG---CCGUCUCAGCUCGCUU-CUCCCCCCA------CCUCCUCUCUCCUCAG
Cat            GUGAGGACUCGG---GAGGUGGAGGGUGGU--GCUGCCAG---GGCCAGGGCA---CUGUCUCAGCUCACUU-CUCCCCCCA------CCUCCUCUCUCCUCAG
Dog            GUGAGGACUCGG---GAGGUGGAGGGUGGC--GCCGCCAA---GGCCAGGGCA---CUGUCUCAGCUCGCUU-CUCCCCCCA------CCUCCUCUCUCCUCAG
Microbat       GUGAGGACUCGG---GAGGUGGAGGGUGGU--GUUGCCAG---GGCCAGGGCA---CUGUCUCAGCUCACUU-CUCCCCCCA------CCUCCUCUCUCCUCAG
Megabat        GUGAGGACUCGG---GAGGUGGAGGGUGGU--GUU---------GCCAGGCCG---UUGUCUCAGCUCACUU-CUCCCCCCA------CCUCCUCUCUCCUCAG
Hedgehog       GUGAGGACUCGG---GAGGUGGAGGGUGGC--GCUGGCAG----GCCCCGGCG---CCGGCUCAGCUCGCC---UCCCCCCA------CCUCCUCUCUCCUCAG
Elephant       GUGAGGACUCGG---GAGGUGGAGGGUGGC--GUCGCCAA---GGCCAGGGCG---CUGUCUCAGCUGGCCU-CUCCCCCCA------CCUCCUCUCUCCUCAG
Rock_hyrax     GUGAGGACUCGG---GAGGUGGAGGGUGGU--GUCACCAG---GGCCAGGGCA---CAGUCUCAGCGCGCUC-CUCCCCCCA------CCUCCUCUCUCCUCAG
Tenrec         GUGAGGACUUGG---GAGGUGGAGGGCGGC--GUGGCCGGCCCGGC--GGGCG---CCGUCUCAGCUCGCCG-CGCCCCCCA------CCUCCUCUCUCCUCAG
Armadillo      GUGAGGACUCGG---GAGGUGGAGGGUGGC--CUCUUGAG---GGCC-GGGCG---UUGUCUCAGUUCGCUUCUUCCCCCCA------CCUCCUCUCUCCUCAG
Sloth          GUGAGGACUCGG---GAGGUGGAGGGUGGA--CUUGUCAG---GGCCGGGGCG---CUGUCUCAUCUCGCUU-UUCCCCCCA------CCUCCUCUCUCCUCAG
Wallaby        GUGAGGGUACAGACAGGGAUGGAAGGAA----UUUGGCAG---GGGUGGGGCA---GAGCCU-GGCCUGCUC-CUCAACCUGAGCCUGCCCUGCCCCCCUCACAG
```

hsa-mir-1229

```
Human          GUGGGUAGGGU--------------UUGGGGG-----------A-GAGCG------------------------------------------UGGG--CUGGGGUUCAG--------------GGACACC-CUCUCACCACUGCCC-----UC---CCA----CAG
Chimp          GUGGGUAGGGU--------------UUGGGGG-----------A-GAGCG------------------------------------------UGGG--CUGGGGUUCGG--------------GGACACC-CUCUCAGCGCUGCCC-----UC---CCC----CAG
Gorilla        GUGGGUAGGGU--------------UUGGGGG-----------A-GAGCG------------------------------------------UGGG--CUGGGGUUCGG--------------GGACACC-CUCUCAGCGCUGCCC-----UC---CCC----CAG
Orangutan      GUGGGUAGGGU--------------UUGGGGG-----------A-GAGCG------------------------------------------UGGG--CUGGGGUUCGG--------------GGACACC-CUCUCAGCGCUGCCC-----UC---CCC----CAG
Rhesus         GUGGGUAGGGGG-------------CUGGGGG-----------A-GAGUG------------------------------------------UGGG--CUGGGGUUCGG--------------GGACACC-CUCUCAGCGCUGCCC-----UC---CCC----CAG
Marmoset       GUGGGUAGGGG--------------CUGGGGG-----------A-GAGCG------------------------------------------UGGG--CUGCGGUUCAG--------------GGACACC-CUCUCAGCACUGCCC-----UC---CCC----CAG
Mouse_lemur    GUGAGUGGGAA---------------CGGGG------------A-GAGCAGAUGCUGGGCUGAGCAGGCAGGCCCUGUU-------------UGGG--CUGGGGUUUGG--------------GGACACC-CUCUCAGCACUGCCC-----UC---CCC----CAG
Bushbaby       GUGAGUAGGGG---------------UGGGGG-----------A-GAGCUUGUGCUGGGCUGAGCAGGCAGUCCCUGUU-------------UAGG--CUGGUGUUUGGC-------------GGACACC-CUCCCAGCACUGCCU-----UC---CCC----CAG
Mouse          GUGAGUAGGGC----------------------------------GGGCC-------------------------UGUG-------------UGGG--CUGGG-UUUGGGUGUUGCUUUCG--UGUUUAC-AUCUAGGCACCGCGC-----UC---CCA----CAG
Rat            GUGAGUAGGGC--------------------------------A-GGGCC-------------------------UGUG-------------UGGG--CUGGG-UUUGGGUGUUGUUGUUGGGUGUUUAC-AUCUAGGCACUGCGC-----UC---CCA----CAG
Kangaroo_rat   GUGAGCGGG------------------------------------GAGCC-------------------------AGGC-------------UAGG--CUGAGGUUUGGGCUAGGUGUCUG--UUUUGCC-AACUGAAAACCUUUC-----UCAUCUCG----CAG
Guinea_Pig     GUGAGCGUUGC--------------------------------A-GGGCC----------------------CAGUGGG-------------UGCA--UUGUGAGGUGGGAGCCCAGGCCG--CCUGGCCUCUCUGAGCUCCUUGCAACCUCC---CUG----CAG
Rabbit         GUGAGGGGGGG--------------------------------G-GAGUG----------------------CUCUCGG-------------CGGG--AGGGGGCUGGGCACGC--------CCCGGAC-CUCACGGCCCCACCC-----UC---UUC----CAG
Alpaca         GUGAGUAAGGGGCAGUCAGGUGGAGGUGGGGG--------GA-G-AGGCU-------------------------GGAC-------------UGAC--CAAGGGUCUUG--------------GGGCAAC-CUCUCAUUGCUGCCU-----UC---CCC----CAG
Dolphin        GUGAGUGAGGGGCAGUCAAGUGGAGCUGAGGG--------GA-G-AGGC-------------------------------------------------AGGAGUCUUG--------------GGACAAC-CUCUCAUUGCUGCAC-----UC---CCC----CAG
Cow            GUGAGUGAGGGGCUACGGAGCGGAGGUGGGGGCGAGGCUCGA-U-GAGC-------------------------------------------------AGGGCUCUUG--------------GGGCCAC-CUCUCACUGCUGCGC-----UC---CCC----CAG
Horse          GUGAGUGG-GGUCAGGCCCGCAGAGGUGUGGG--------A--G-AGGCU-------------------------GGGC--------------GA---CAGGGGUCUUG--------------GGACGGC-CUCUCAGCGCUGCCC-----UC---CCC----CAG
Cat            GUGAGUGGAGGG--GGCAGGUGGAGGUGGGG------------G-CAGUA-------------------------GGCU--------------AAG--UUGGGGUCUGG--------------AGGCAAU-CUCUCAGCGCUGCCC-----UC---CCU----CAG
Dog            GUGAGUGGGGGGCAGGCAGGUGGACAUGAGGA--------GUGG-AGGCA-------------------------GGGC--------------GAG--CAGAGGUCUGG--------------GGGCAGU-GGCUCAGUGCUGCCC-----UC---CCUUCCUCAG
Microbat       GUGAGUGG-GGUCCGGCGGGCAGAGGUGGGGG--------G--G-AGGCU-------------------------GGGC-------------UGAG--CAGGGGUCUUG--------------GGACGAG-UUCUCAGCACUGCCG-----UC---CCC-----AG
Megabat        GUGAGUGC-UGUU-GGCAGGCAGAGGUGUGGG--------G--ACAGGCU-------------------------GGAC-------------UGAG--CAAGGACCUGG--------------GGGUAAG-CUCUCAGUGCCGCCC-----UC---CCC----CAG
Shrew          GUGAGUUGGGGU-----GGGCGAGGGCGGGGA-----------G-GGGCA-------------------------GGGG-------------GGAAGCGGGGAGGCUCA--------------AACUGGG-CUGUC-----CGCCU-----UC---CCC----CAG
Rock_hyrax     GUGAGUUGGGC-------------GGUGGGGC-----------A-GGGCU-------------------------GAGCAGCCAAGACGCCCGGGG--CCGGGUCUCCG--------------GGACCCC-CUCUCAGUGCUGCCC-----GC---CCC----CUA
Opossum        GUGAGUAG---------------------GCC-----------A-GAGCC----------------------AGGAAGA-------------UGAG--CAGAGGCUCUG--------------AGGUGCC-CCUCCA--------------CC---CCG----CAG
```

hsa-mir-3064

```
Human          UCUGGCUGU--UGUGGUG----UGCAAAACUCCG---UACAUUGCUAUUUUGCCACACUGC--AACAC---CUUACAG
Chimp          UCUGGCUGU--UGUGGUG----UGCAAAACUCCG---UACAUUGCUAUUUUGCCACACUGC--AACAC---CUUACAG
Gorilla        UCUGGCUGU--UGUGGUG----UGCAAAACUCCG---UACAUUGCUAUUUUGCCACACUGC--AACAC---CUUACAG
Orangutan      UCUGGCUGU--UGUGGUG----UGCAAAACUCCG---UACAUUGCUAUUUUGCCACACUGC--AACAC---CUUACAG
Rhesus         UCUGGCUGU--UGUGGUG----UGCAAAACUCCG---UACAUUGCUAUUUUGCCACACUGC--AACAC---CUUACAG
Baboon         UCUGGCUGU--UGUGGUG----UGCAAAACUCCG---UACAUUGCUAUUUUGCCACACUGC--AACAC---CUUACAG
Marmoset       UCUGGCUGU--UGUGGUG----UGCAAAACUCCG---UACAUUGCUAUUUUGCCACACUGC--AACAC---CUUACAG
Tarsier        UCUGGCUGU--UGUGGUG----UGCAAAACUCCG---UACAUUGCUAUUUUGCCACACUGC--AACAC---CUUACAG
Mouse_lemur    UCUGGCUGU--UGUGGUG----UGCAAAACUCCG---UACAUUGCUAUUUUGCCACACUGC--AACAC---CUUACAG
Bushbaby       UCUGGCUGU--UGUGGUG----UGCAAAACUCCG---UACAUUGCUAUUUUGCCACACUGC--AACAC---CUUACAG
TreeShrew      UCUGGCUGU--UGUGGUG----UGCAAAACUCCG---UACAUUACUAUUUUGCCACACUGC--AACAC---CUUACAG
Mouse          UCUGGCUGU--UGUGGUG----UGCAAAACUCCG---UACAUUGCUAUUUUGCCACACUGC--AACAC---CUUACAG
Rat            UCUGGCUGU--UGUGGUG----UGCAAAACUCCG---UACAUUGCUAUUUUGCCACACUGC--AACAC---CUUACAG
Kangaroo_rat   UCUGGCUGU--UGUGGUG----UGCAAAACUUCG---UACAUUGCUAUUUUGCCACACUGC--AACAC---CUUACAG
Guinea_Pig     UCUGGCUGU--UGUGGUG----UGCAAAACUCUG---UACAUUGCUAUUUUGCCACACUGC--AACAC---CUUACAG
Rabbit         UCUGGCUGU--UGUGGUG----UGCAAAACUCCA---UACAUUGCUAUUUUGCCACACUGC--AACAC---CUUACAG
Pika           UCUGGCUGU--UGUGGUG----UGCAAAACUCCG---UACAUUACUAUUUUGCCACACUGC--AACAC---CUUACAG
Alpaca         UCUGGCUGU--UGUGGUG----UGCAAAACUCCG---UACAUUGCUAUUUUGCCACACUGC--AACAC---UUUACAG
Dolphin        UCUGGCUGU--UGUGGUG----UGCAAAACUCCG---UACAUUGCUAUUUUGCCACACUGUCUAACAC---CUUACAG
Cow            UCUGGCUGU--UGUGGUG----UGCAAAACCCUG---UACAUUGCUAUUUUGCCACACUGC--AACAC---CUUACAG
Horse          UCUGGCUGU--UGUGGUG----UGCAAAACUCCG---UACAUUGCUAUUUUGCCACACUGC--AACAC---CUUACAG
Cat            UCUGGCUGU--UGUGGUG----UGCAAAACUCCG---UACAUUGCUAUUUUGCCACACUGC--AACAC---CUUACAG
Dog            UCUGGCUGU--UGUGGUG----UGCAAAACUCCG---UACAUUGCUAUUUUGCCACACUGC--AACAC---CUUACAG
Microbat       UCUGGCUGU--UGUGGUG----UGCAAAACUCCA---UACAUUGCUAUUUUGCCACACUGC--AACAC---CUUACAG
Megabat        UCUGGCUGU--UGUGGUG----UGCAAAACUCCG---UACAUUACUAUUUUGCCACACUGC--AACAC---CUUACAG
Hedgehog       UCUGGCUGU--UGUGGUG----UGCAAAACUUCG---UACAUUGCUAUUUUGCCACACUGC--AACAC---CUUACAG
Shrew          UCUGGCUGU--UGUGGUG----UGCAAAACUCCG---UACAUUGCUAUUUUGCCACACUGC--AACAC---CUUACAG
Elephant       UCUGGCUGU--UGUGGUG----UGCAAAACUCUG---UACAUUGCCAUUUUGCCACACUGC--AACAC---CUUACAG
Rock_hyrax     UCUGGCUGU--UCAGCUC----UGCAGAACU--G---UAUGUUGCU-UUUUGCCACACAAC--AACAC---CUUACAG
Tenrec         UCUGGCUGU--UGUGGUG----UGCAAAACUCCG---UACAUUGCUCUUUUGCCACACUGC--AACAC---UUUACAG
Armadillo      UCUGGCUGU--UGUGGUG----UGCAAAACUCCG---UACAUUGCUAUUUUGCCACACUGC--AACAC---UUUACAG
Sloth          UCUGGCUGU--UGUGGUG----UGCAAAACUCCG---UACAUUGCUAUUUUGCCACACUGC--AACAC---CUUACAG
Opossum        UCUGGCUGU--UGUGGUG----UGCAAAACUUCC---UAUGUUGCUUUUUUGCCACACUGC--AACAC---UUUACAG
Platypus       UCUGGCUGU--UGUGGUG----UGCAAAACUUCG---UACAUUGCUUUUUUGCCACACUGC--AACAC---UUUACAG
Chicken        UUUGGCUGU--UGUGGUG----UGCAAAACUUUG---UACCUUGC-UUUUUGCCACACUGC--AACAC---UUUACAG
Zebra_finch    UUUGGCUGU--UGUGGUG----UGCAAAACUUUG---UACCUCGC-UUUUUGCCACACUGC--AACAC---UUUACAG
Lizard         UCUGGCUGU--UGUGGUG----UGCAAAUUUUUG---UGCAUUGC-CUUUUGCUGCACUGC--AACAC---UUUACAG
X_tropicalis   UCUGACCCU--UGUGGUG----UGCAAAACCUUA---U---UUGCUGUUUGCCACACUGC--GACAC---UCUACAG
Tetraodon      UCUGGCUGU--UGUGGUG----UGCAAAAUCCUG---UGUG---GUGUUUUGCCACACUGC--AACAC--UUUCACAG
Fugu           UCUGGUUGU--UGUUGUGGCUCUGGCACACCUUU---UGUGCUAUUACUGUGCCACACUUC--AACAUAAUCUUGCAG
Stickleback    UCUGGUUGU--UGUGGUGGCGCUGGCGCACGUCUAUGUGUGCUGUCUUUAUGCCACACCGC--AACACAAUCUUUCAG
Medaka         CCUGGUUGU--UGUGGUGGCUCUGGCACACUUCU----GUGCUGCUUUUCUGCCACACGGC--AACGCAACUUUACAG
Zebrafish      CCUCGCUGC--UAUGGCGGUGU--GCACCCGCAC----------UUGCCAUGGCACGCC-C--GUUUUGUCUUAACAG
Lamprey        UGUGGCUGUCUUGCGGUG----UGUAAA-------------------UGGCGCCUUGCUGC--GUCAC---CCUUCAG
```

hsa-mir-6745

```
Human          UGGGUGGA------AGAAGG------------UCU---GGUU-------CUCA--C-CA---GGCC-----C-C-UCCUCUCC--C-------A------CCCAG
Chimp          UGGGUGGA------AGAAGG------------UCU---GGUU-------CUCA--C-CA---GGUC-----C-C-UCCUCUCC--C-------A------CCCAG
Gorilla        UGGGUGGA------AGAAGG------------UCU---GGUU-------CUCA--C-CA---GGCC-----C-C-UCCUCUCC--C-------A------CCCAG
Orangutan      UGGGUGGA------AGAAGG------------UCU---AGUU-------CUCA--C-CA---GACC-----C-C-UCCUCUCC--C-------A------CCCAG
Rhesus         UGGGUGGA------AGAAGG------------UCU---GGUC-------CUCA--C-CA---GAAC-----C-C-UCCUCUCC--C-------A------CCCAG
Baboon         UGGGUGGA------AGAAGG------------UCU---GGUC-------CUCA--C-CA---GAAC-----C-C-UCCUCUCC--C-------A------CCCAG
Marmoset       AGAAUGGA------AGAAGG------------UCU---GGUC-------CUCA--C-CA---GGCC-----C-C-UCCUCUCC--C-------A------CCCAG
Mouse_lemur    UGCAUGGA------UAAAGG------------UCU---UGUC-------CUCA--C-CA---GACC-----C-CAUCCCCUCC--C-------A------UCUAG
Bushbaby       UACGUAGA------UGAAGC------------UCU---CCUC-------CUCA--U-UU---GACC-----C-UUUCCCCUCU--C-------A------CCCAG
TreeShrew      UGAGUGGG------GGAAGG------------UCU---GGUC-------CUCA--C-CA---GCCC-----U-C-UCCUCUCC--U-------G------CUCAG
Mouse          UGAGUGGA------AGGCAG------------U-C---UGUC-------CUCA--C-UG---CCUC-----C-C-UUCUCUCU--C-------A------CCCAG
Rat            UAAGUGGA------AGGCAG------------U-C---UGUC-------CUCA--C-UG---CCUC-----C-C-CUCUCUCU--C-------A------CUCAG
Guinea_Pig     UA--------------------------------C---AGUG-------CUAA--C-UG---GCCC-----C-U-ACCUCUUC--C-------A------CCUAG
Squirrel       UGAGUGGA------GAGUGG------------UCC---CAUC-------CUCA--C-UGGCACCUC-----C-C-UUCUCUUU--C-------A------CCCAG
Rabbit         UGGGUGGA------AGAAGG------------CCU---GGUC-------CUCA--CCUG---GUCC-----C-U-UAUCCUCC--C-------G------UGCAG
Pika           CAGGUGGA------AGAGGGUGACAGUGGCGCCCU---GGUC-------CUCA--CCUG---CUCC-----A-C-AACUGUCU-GU-------U------UGCAG
Dolphin        --------------ACAAGG------------UCU---UAUC-------CUCA--C-CA---GACC----CC-C-UCCUCUCC--C-------A------CCCAG
Cow            UGAGUGGAAGAAGAAGAAGG------------UCU---UGUC-------CUCA--C-CA---GAGC----CC-C-UCCUCUCC--CC------A------CCCAG
Horse          UGAGCGGA------AGAAGG------------UCU---UGUC-------CUCA--C-CA---GGCC----UC-C-UCCUCGCC--C-------A------CUCAG
Cat            UGAGUGGA------AGAAGG------------UCU---UGUC-------CUCA--C-CG---GGCC-----C-C-UCCGGUCC--C-------A------CCCAG
Dog            CGAGUGGA------AGACGG------------UCU---UGUG-------CUCA--C-CA---GGCC-----C-C-UCCUCUCC--C-------A------CCCAG
Microbat       UGAGUGGA------AGGAGG------------UCU---CGUG-------CUGA--C-CA---GGUC----CC-C-UCCUCCCC--C-------A------CCCAG
Megabat        UGAGUGGA------AGAAGG------------UCUUAUCAUC-------CUCA--C-CA---GGCC----UC-C-UCCUUUAC--C-------A------CCCAG
Hedgehog       UGAGUG--------GGAAGG------------UCU---AGCG-------CUCA--C-CA---GGGC----CU-C-ACUCUGCC--C-------C------CUCAG
Elephant       UGGGUGGA------AGAAGG------------CCU---CAUC-------CUCA--C-CA---GGCU-----C-C-UCCCCUCU--C-------A------CCCAG
Rock_hyrax     UGGAUGAA------UGAAGG------------UCU---CAUC-------CUCA--C-CA---GGCU-----C-U-ACCCCUCU--C-------A------CCCAG
Tenrec         CGGGUGGU------AGGAGG------------CUU---C----------CUCA--C-CA---GGCC-----CGU-GCCCCUCA--C-------A------CGCAG
Opossum        CGAGGGGA------GGUGGG------------CCC---CAGCACGGCCGCUGA--C-CG---GCUC-----C-C-UCCCGGCC--U--------------CCCAG
Wallaby        -----------------CUG------------ACC---UGCU-------CCCC--U-UA---CACC-----U-C-UCUGCUCU--CCUGGCUGC------CCCAG
Platypus       GGGAUGGA------GCGAGG------------GCG---AGAG-------CCCG--A-CG---GGCCGGCGUU-C-UCCUCCUC--C-------GGGCGCUGCCAG
X_tropicalis   UGUCUCAA------UGGAGAUGGU-----------------U-------CUCAGGC-UG---GUGU-----C-U-UCCUCCCCAU-----------------CAG
```

hsa-mir-6751

```
Human         UUGG--GGGUG-AGG----U------------------UGGUG--UCUG---------------GCCCCAG---CAGCCCAGACUGAGCCU----------------------------------------CU-----CUCUCUCC----AG
Chimp         UUGG--GGGUG-AGG----U------------------UGGUG--UCUG---------------GCCCCAG---CAGCCCAGACUGAGCCU----------------------------------------CU-----CUCUCUCC----AG
Gorilla       UUGG--GGGUG-AGG----U------------------UGGUG--UCUG---------------GCCCCAG---CAGCCCAGACUGAGCCU----------------------------------------CU-----CUCUCUCC----AG
Orangutan     --GG--GGGUG-AGG----U------------------UGGUG--UCUG---------------GCCCCAG---CAGCCCAGACUGAGCCU----------------------------------------CU-----CUCUCUCC----AG
Rhesus        UGGG--GGGUG-AGG----U------------------UGGUG--UCUG---------------GCCCCAG---CAGCCCAGACUGAGCCUUUCU------------------------------------CU-----CUCUCUCC----AG
Baboon        UGGG--GGGUG-AGG----U------------------UGGUG--UCUG---------------GCCCCAG---CAGCCCAGACUGAGCCU----------------------------------------UU-----CUCUCUCC----AG
Marmoset      UUGG--GGGUG-AGG----U------------------UGGUA--UCUG---------------GCCCCAGAAGCAGCCCAGACUGAGCCUCUCUCUCUCUCUCUCUCUCUCUCUCUCUCUCUCUCUCUCUCU-----CUCUCUCC----AG
Mouse_lemur   UGGA--GGCUG-GGG----U-------------------GGUA--UCAG---------------GCCCAGG---AAGCCCAGACUGAGCCU----------------------------------------CU--A-CCUCUCUCC----AG
Bushbaby      UGGA--GGCUG-GGG----U-------------------GGUG--UCAG---------------GCCAAGG---CAGCACAGACUGAGACU----------------------------------------CU--A-CCUUUCUCC----AG
TreeShrew     --GA--GGAUG-GGU----U------------------GGUUG--UCAG---------------GCCCAAG---CAGCCCAGACUGAGACU----------------------------------------CU--G-CCUCUCUCC----AG
Mouse         UCUG--GGGUG-GAG----A-------------------GCUG----AG---------------GCCCAGG---UAGCUCAGCCUGACCUC----------------------------------------UG--UAUCUCCUGCC----AG
Rat           UUCA--GGUUA-AAG----A-------------------GGUG----AG---------------GCCCAGG---UAGCUCAGACUGACCUC----------------------------------------UG--UGUCUCCUGCC----AG
Kangaroo_rat  UUGG--GGGUA-AAGCAUCA-------------------GCUG----AA----------------CCCAGG---CAGCCCAGACUUAAGUU----------------------------------------UC--CACCUUUCUCC----AG
Guinea_Pig    UAGA--GGGCG-GAC----A-------------------GGCA----GGCAGGGGUGCCUGAUGGCUCAGG---UAGCCCCAACUUACCUC----------------------------------------CC--C--CUACCCCC----AG
Squirrel      UAGA--AGGUG-UAG----G-------------------GGGGUAUCAG---------------GCCCAGG---UAGC--AAACUGAGCCU----------------------------------------CC--A--CCUCUUCC----AG
Rabbit        CAGG--GGGAA-GGC----G-------------------GUGG----GG---------------GGC--GG---CAGCGCUAACGG---CC----------------------------------------UC--U--CCCUCUCC----AG
Pika          ------------GAA----G-------------------GUGA----GG---------------GACCAGG---CAGCCCUGACUGGAUCU----------------------------------------UC--A--CUCCCUGC----AG
Dolphin       UGGG--AGGUGUGGG-----------------------GGUG--UCAG---------------GCCCAGG---CAGCCCAGGCUGAGCCU----------------------------------------CU--C-UCUCUCUCC----AG
Cow           GGGG--AGGUG-GGG-------------------------GGUG--UCAG---------------GCCCAGG---C---------UGAGCCU----------------------------------------CU--C-UCUCUCUCCCUUCAG
Horse         CAGA--GGGCG-GCG-------------------------GGUG--GCAG---------------GCCCAGG---CAGUCCAGGCUGAGCCU----------------------------------------UU-------CUCUCC----AG
Cat           UGGA--GGGAU-CUG-------------------------GGCA--UCAG---------------GCCCAGG---CCGCCCCGGCUGAGCCU----------------------------------------CUCUG-CCUCCCUCC----AG
Dog           UGGA--GGGUA-GGG-------------------------GGUG--UUAG---------------GCCCAGG---C---------UGAGCCU----------------------------------------CU--G-CCUCUCUCC----AG
Megabat       UGGA--AGGCA-GGG-------------------------CUUG--UCAG---------------GCCCAGA---CAGCCCGGGUUAAGCCU----------------------------------------CU--A-CCUCUCUCC----AG
Hedgehog      UGGG--GGCCC-AGG-------------------------G-----UGGG-------------------CGG---ACGGCCCUGUUGAGCCC----------------------------------------CU--G-CCUCCCUCC----AG
Shrew         UGGG--AAGCA-GGG-------------------------C-----UCGG---------------GCCCUGG---ACGCCCCGGCUGAGCCC----------------------------------------CC--G-CCUCUCCGC----AG
Elephant      UGGA--CGGGG-AGG-------------------------GUGG--GCCA---------------GGCCCAG---CAGC-CAGUUUGAGCUA----------------------------------------CU--G-CCUCUCCCC----AG
Opossum       UUUU--GGGUU-GGG----GAGCCUUUGGUUCCCUCUGUGGGG--CCCA---------------GCUCGAA---UAUCUUUGGCACAUCUG----------------------------------------CU--UUGUGCUCUUC----AG
Wallaby       CAGACUGGGCA-CAG----G-------------------GAGG--CCUU---------------GGUCAAG---CAGCUUGGGCAUACCCU----------------------------------------CU--U--CUCUCUAC----AG
X_tropicalis  CACA--GGGUU-CAU----C------------------UUGUU--UCAU---------------UCUACAU---CAUUUUGACCAUAAUUU----------------------------------------AU--C--CAUCACAC----AG
```

hsa-mir-6767

```
Human         UCGCAGACAGGGAC----------------ACAUGGAGAACGCCCCCACCAGUUCCCA--CGUGCUUC--UCUUUCCGCAG
Chimp         UUGCAGACAGGGAC----------------ACAUGGAGAACGCCCCCACCAGUUCCCA--CGUGCUUC--UCUUUCCGCAG
Orangutan     UUGCAGACAGGGAC----------------ACACGGAGAACGCCCCCACCAGUUCCCA--CGUGCUGC--UCUGCCCACAG
Rhesus        UUGCAGACAGGGAC----------------GCAUGGAGAAUGCCUGCCCUAGUUCCCA--CGCGCUGC--UCUUUCCGCAG
Baboon        UUGCAGACAGGGAC----------------GCAUGGAGAAUGCCUGCCCUAGUUCCCA--CGCGCUGC--UCUUUCCGCAG
Marmoset      UUGCAAGCAGGGAC----------------A-----------CCUGCCCUAGUU--CA--GCUGCUGC--UGUAUCCACAG
Tarsier       UUGGAGACAGGGAC----------------ACUUUGAGAACACAUGCCCUCGUUUUCAGGGGUGGUGC--UCUUUCCACAG
Mouse_lemur   CUGGAGAC--GGGC----------------GCCUGGAGGACCGGUGCCCUGGUGUUCA-GGGCAGCAC--UCUUUCCACAG
Mouse         GGGC-AGCUGUGAU----------------GCU-----------UGUCCUAGUUUUCA--CGGGUGGCCCUCUUUCUGCAG
Squirrel      ------GUUGGGAC----------------AUGCAGGGAAGC-UUGUCCUAGUUUUCA--AGGGUGGCUCUCUCUCCACAG
Rabbit        GGAU-GGUUGGAGG----------------GCU---UGUAGCGCGGUGCCGGCC--------------C--UCUGCCCACAG
Dolphin       UGGGAGACG-GGAC----------------AC-UCAUGAAGGCUUGGCCUGGUUUUCA--GGGAUGGC--UCUUCCCACAG
Cow           UGGGAGACA-GGACCCCGUGAGGUUCGGGGC-UCACAGAGGCUCUGCCUGGUGUUCA--GGGGCAGC--UCUUCCCACAG
Horse         UGGGGGACA-GGAC----------------AC-UCGUGAAUGCUCCCCUUAGUUUUCA--GGGAUGGC--UCUUC--GCAG
Cat           UG--------GGAC----------------GC-UCACAAAUGUGCGCCCGAGUUUCCA--GGGCUGGC--UCUCCCUGCAG
Microbat      UGGGAGACA-GGAC----------------AC-UCAUGAGCGCUCGCUCUAGUGUUCA--GGGGUGGC--UCUUCCCAAAG
Megabat       UGGGAGACA-GGAC----------------GU-UUGUGAAUACUCGCUCUAGUGUUCA--GGGGUGGC--UCUUCCCACAG
```

hsa-mir-6777

```
Human         ACGGG-GAG---UCAGGCAGUGG---------------UGGAGAUGGAGA------GC----CCUGAGCCUCCACU-----CUCCUGGCC-------CCC-------AG
Chimp         ACGGG-GAG---UCAGGCAGUGG---------------UGGAGAUGGAGA------GU----CCUGAGCCUCCACU-----CUCCUGGCC-------CCC-------AG
Gorilla       ACGGG-GAG---UCAGGCAGUGG---------------UGGAGAUGGAGA------GC----CCUGAGCCUCCACU-----CUCCUGGCC-------CCC-------AG
Orangutan     AUGGG-GAG---UCAGGCAGUGG---------------UGGAGAUGGAGA------GC----CCUGAGCCUCCACU-----CUCCUGGCC-------CCC-------AG
Rhesus        AUGGG-GAG---UCAGGCAGUGG---------------UGGAGAGGGACA------GC----CCUGAGCCUCCACU-----CUCCUGGCC-------CCC-------AG
Baboon        AUGGG-GAU---UCAGGCAGUGG---------------UGGAGAGGGACA------GC----CCUGAGCCUCCACU-----CUCCUGGCC-------CCC-------AG
Marmoset      AUGGG-GAGGAGUCAGGUAGUGG---------------UGGAGAGGGAAG------AG----CCUGAGUUUCCACU-----CUGCUGGUC-------CCCCUUGCUCAG
Mouse_lemur   UUGGG-GAC---AUAA--AGCCG---------------UGUGUGGAAGGA------GC----CCUGAACCUGGACU-----GUCCUGGCCUUCUCG-CCC-------AG
Bushbaby      CUGGG-GAC---AUAA--AGCAG---------------UGUGUGUGUGUA------GC----CCUGAACCUGGACU-----CUUUGGCCCUACUCA-CCC-------AG
TreeShrew     AGGGG-GCG---CUGGGCGGUGGGUGCUGUGCAGGGGGCGGGAGUGCAGA------GCUGGGCCUGAGCCUCUGCU-----CUC-----C-------CGC-------AG
Mouse         GAGGG-GCA---UGAGGUAGGAAGCC---GGGAGU---GGGGGGACAGUA------AA----UCUGAAACGGGUGA-----CUACU--CCUUGCCU-CUC-------AG
Rat           GUGGG-GUG---UGAGGCAGGGAGC-------------GAGGGGACAGUC------AU----CCCGAAACUAGCCA-----UUACU--CCUCACCC-CUC-------AG
Kangaroo_rat  GUGGGUACU---UGAAGCAGCAGAUACGGGGGAGA---GGGGGGACAG--------GG----CCUGAACCUGGCCU-----CUCCUGGCCCCACCCACUC-------AG
Squirrel      GUGAGGACC---UGAAGCAACAGUUGCCUGGAGG----GAGAGAGCAG--------GG----ACGGAACUUCAUCU-----CUGCUGCCCCU-----CCC-------AG
Dolphin       --GGG-AUG---UA---UGUGAGGUGUCGGGAGG----GAGCGGGGGCCU------GG----CUGGAACCUCUCCUGGCCCCUCCUG----------CCC-------AG
Cow           --GGG-ACA---GA---CAUGAGGUGUCUGAAGG----GAGUGGGG-CCU------GA----CCUGAACCUCUCCUGGUUCCUCCUG----------CCC-------AG
Dog           --GGG-AUG---UGAGGCAGCAGG-----GGAGG----GAGGGAUGGCCC------GG----CGCGAACCCCAACU-----CUCCUGGC--------CCC-------AG
Microbat      --GGC-ACA---GGAAGCAGAUGGUG-------G----GAGAGGGGGCCU------GG----CCUGAACCCCGACU-----CACCCGGC--------------------
Megabat       --GGG-ACA---UGAGGCAGCUGGUGUGGGGAGA----GAGUGGGGCCCU------GG----CCUGAGCCCCUGCU-----CUCCCGGC--------CCC-------AG
Hedgehog      --GGG-AGA---GUGGACGGGCUGCA---GGUGA----CGGUCCUGGCCU------GA----CCUUAACCCCAACA-----UCCCUUGC--------CCC-------AG
Elephant      --GGG-GUG---GGGGUUGGU-----------------GGCUGCAGCCAA------GC----CCUGACUACCUG-G-----CACCUGCC--------CUC-------AG
Tenrec        --GAG-GCG---GAGGUCAG------------------GACUGCAGCCAA------GU----CCUGACUGCCCCCG-----CGCCUGCC--------CUC-------AG
Armadillo     --CGC-CGG---GGGGACGGCUCCCC------------GCCAGCCGCCA--------C----CCUGACCCCC---A-----CCCGCUCC--------CCC-------AG
Opossum       ---GG-GAG---CUUUGUGGCC----------------UGGGGAAGGGCACCCCCUUU----CCCAAGCCCCGACU-----CCUUU--------------CUU-------AG
Wallaby       AGGGG-GCC---ACAGGUAUGGG---------------AAGGGGAACUCC------CU----UUGAAGACCCCCUG-----UUUCUGUU--------UUU-------AG
Platypus      CCGGG-CAA---ACAAUCGGUGC---------------UGUUUACUGAUC------G-------GGGCGUCCAUC-----UCGGU---------------------AG
```

hsa-mir-877

```
Human          GU-AGAGGAGA-----U--GGCGCAGGGGACACGGGCA--AAG----A-CUUG------GGGGUUCCUGGGACCCUCAGACGUGUGUCCUCUUCUCCCUCCUCCCAG
Chimp          GU-AGAGGAGA-----U--GGCGCAGGGGACAUGGGCA--AAG----A-CUUG------GGGGUUCCUGGGACCCUCAGACGUGUGUCCUCUUCUCCCUCCUCCCAG
Gorilla        GU-AGAGGAGA-----U--GGCGCAGGGGACACGGGCA--AAG----A-CUUG------GGGGUUCCUGGGACCCUCAGACGUGUGUCCUCUUCUCCCUCCUCCCAG
Orangutan      GU-AGAGGAGA-----U--GGCGCAGGGGACACGGGCA--AAG----A-CUCG------GGGGUUCCUGGGACCCUCAGACAUGUGUCCUCUUCUCCCUCCUCCCAG
Rhesus         GU-AGAGGAGA-----U--GGCGCAGGGGACACGGGCU--AAG----A-CUCG------GGGGUUCCUGGGACCCUCAGACAUGUGUCCUCUUCUCCCUCCUCCCAG
Baboon         GU-AGAGGAGA-----U--GGCGCAGGGGACACGGGCU--AAG----A-CUCG------GGGGUUCCUGGGACCCUCAGACAUGUGUCCUCUUCUCCCUCCUCCCAG
Marmoset       GU-AGAGGAGA-----U--GGCGCAGGGGACACGGGCU--AAA----A-CUCG------GGGGUUCCUGGGGCUCUCAGACAUGUGUCCUCUUCUCCCUCCUCCCAG
Mouse_lemur    GU-AGAGGAGA-----U--GGCGCAGGGGACACAGGU---AAG----A-CUU-------GGGGUUCUUGGGGCCCUUGAACAUGUGUCCUCUUCUCCCUCCUCCCAG
Mouse          GU-AGAGGAGA-----U--GGCGCAGGGGACACAAGGU--AGG----C-CUUG------CGGGUCUGUGGA-CCCUUGGACAUGUGUCCUCUUCUCCCUCCUCCCAG
Rat            GU-AGAGGAGA-----U--GGCGCAGGGGACACAAGGU--AGG----C-CUUG------CGGGUCUGUGGG-CCCUUGGACAUGUGUCCUCUUCUCCCUCCUCCCAG
Kangaroo_rat   GU-AGAGGAGA-----U--GGCGCAGGGGACACGAGCU--AAG----A-CUCA------GGGGUCCCCGGA-CCCUCAGACAUGUGUCCUCUUCUCCCUCCUCCCAG
Guinea_Pig     GU-----GAAG-----U--GACAC-GGGGACGGGUGGU--GGGGAGAC-CU---------GGGUCCCUGG--CCUUCGGUCCCACGUCCUCU------CCCUCCUAG
Squirrel       GU-AGAGGAGA-----U--GGCACAGGGGACAUGGUCUGCGAG----A-CUUGGGGGGAGGGGCCUCUAGG-CCCUCAGACAUGAGUCCUCUUCU---UCCUCCCAG
Rabbit         GU-AGAGGCAG-----U--GGCACAGGGCGCCUGGGCU--GAG----A-UUCG------GGGGUGCCGGGG-CCCUGUGGCAUGCGUCCUCUCCUCUGCCAUCCCAG
Pika           GU-AGAGGAGG-----C--GGUGCGGGGACCCGGGCGU--GAG----C-CGCG------GGUGU-CCCUGG-CCCUCAGACGUG---CCCCAUUCCUGCUCCUCCAG
Alpaca         GU-AGAGGAGA-----U--GGCGCAGGGGACAC-GGCU--GAG----A-C-UG------GGG--UCCCGGGACUUCCAAACAUGUGUCCUCUUCCCCUUCCUCCCAG
Dolphin        GU-AGAGGAGA-----U--GGCGCAGGGGACACGGGCU--AAG----A-C-UG------GGGACUCCUGGGACCUCGCAGCAUGUGUCCUCUUCUCCCUCCUCCCAG
Cow            GU-AGAGGAGA-----U--GGCGCAGGGGACACGGGCU--AAG----A-C-UG------GGGGCUCCCGGGACCCCCCAACAUGUGUCCUCUUCUCCCUCCUCCCAG
Horse          GU-AGAGGAGA-----U--GGCGCAAGGGACACAAACG--AAG----A-CUUG------GGGGUUCCUGGGACCUUCAGACAUAUGUCCUCUUCUCCCCCCUCCCAG
Cat            GU-AGAGGAGA-----U--GGCGCAGGGGACAUGGGCU--AAG----A-GUUG------GGGGUUCCUGGAACCUUUGGACAUGUGUCCUCUUCUCCCUCCU-CCAG
Dog            GU-AGAGGAGA-----U--GGCGCAGGGGACAUGGGCU--GAG----A-CUUG------GGAGUUCCUAGAACCUCUAGACAUGUGUCCUCUUCUCCCUCCU-CCAG
Megabat        GU-AGAGGAGA-----U--GGCGCAGGGGAUACGGACU--AAG----A-CUUG------GGGGUUCCUGCAAUCUUCAGACCUGUGUCCUCCU------CUUCUCAG
Hedgehog       GU-AAAGAACA-----U--GACAU-----ACAUGGGCU--AGA----G-UUUG------GGGGUUCCUGGAACCUUUGAACAUGUUUCCUUAUCUUCUCCCUCCUAG
Shrew          GU-ACAGCGAA-----U--GGCAUAGAGGACAAGGUUU--CAA----GAUUUG------GGGGUCCCUGGGGCCUCUGGACAUGCGUCUUCCUCU--CUCCUUCCAG
Elephant       GU-AGAGGAGA-----U--GGCGCAGGGGACACAGGCU--AUG----A-CAUG------GGGGCUCCUGGGGCCCUUGAACAUGUGUCCUCUUCUCCCUCCUCCCAG
Rock_hyrax     GU-AGAGGAGA-----U--GGCGCAGGGGACACAGGCU--GUA----A-CACA------GGGACUCCUGGGGCCCUUAAACAUGUGUCCUCUUCUCCCUCCUCCCAG
Tenrec         GU-AGAGGAGA-----U--GGCGCAGGGGACACGGGCG--GAG----A-C-UG------GGGCUGGCUGGGGUCCUCCAACAUGUGUCCUCUUCUCCCUCCUCCCAG
Opossum        GU-GAAGGACA-----UUCAGAGAACAGUACAUAGGGA--GAC----A-UUCA------GGGACUUUCAGGACUUAAAAUCAUCAUUAUCCUUUUACCUUCUUCCAG
Medaka         GUUAUUUCUG------U--GUUUG--AGGAGAAAAAUG--UCA----G-UCUGCC----UUGGUUUUUGGUUUUCAUGAUGACUAGCUAUCAACUUCCUUCU-CCAG
Zebrafish      GUCAGAUAAAAGGAGCU--CACUG--AGG----GCAUG--CGA----A-CGUG------CUGAUCCAUGGAGUUGCGAAGGAUAA--------------GCUCUCAG
```

uc002nhg.3

```
Human          CCAGGG--U----GGGAUG---AGGCUUGGGAAAAAUAGAAGCAGCCCUCUUGGCCUCAC--CC-UCUCUAG
Chimp          CCAGGG--U----GGGAUG---AGGCUUGGGAAAAAUAGAAGCAGCCCUCUUGGCCUCAC--CC-UCUCUAG
Orangutan      CCAGGG--U----GGGAUG---AGGCUUGGGAAAAAUAGAAGCAGCCCUCUUGGCCUCAC--CC-UCUCUAG
Rhesus         CUGGGG--U----GGGAUG---AGGCUUGGGAAAAAUAGAAGCAGUCCUCUUGGCUUCAC--CC-UCUCUAG
Baboon         CUGGGG--U----GGGAUG---AGGCUUGGGAAAAAUAGAAGCAGUCCUCUUGGCCUCAC--CC-UCUCUAG
Marmoset       CCAGGG--U----GGGAUG---AGGCUUGGGAAAAAUAGAAGCAGCCCUCUUGGCCU-----CC-UUUCUAG
Bushbaby       CUGAGG--U----GGGAUG---AGGCAU-GAAAGAAUAGAACAUUCCCUCUUGGCCUCCC--GG-UCCCUAG
Squirrel       CGCAGC--C----AAUA-G---ACCCCG----------------CCCCUCUUGGCC-UCC--UGGUCCACAG
Dolphin        CCGAAG--U----GGGAGG---AGGCGUGGAA----------ACCCCCUCUUGUCCAUUCCCUG-UCCCCAG
Cow            CUGAAG--UGGAAGGGAGG---AAGCGUGGAAGUAAUAGAACGCCCCCGCCUGUCCAAUCCGUG-UCCCCAG
Cat            CUGAGGA-G----GGGAGG---AGACAUGGAAAGGAUAGAAACCUCCCUGUGUUCCGCUU--GG-UCCACAG
Dog            CUGGGGGUG----GGGGGG---AAGCAUAGAAGCGAUAGAAACUUCCUUCCUGUCCACUU--CG-UCCACAG
Megabat        CUGAGG--U----AGGAGG---AGGCAUGGAAACAAUAG-AACAUCCUUCUUGUCCAUUU--UG-UCCCCAG
X_tropicalis   CCUAGA--U----GUGAUUCAAAAAUAUGGAGAGAUAAAAAAU-UUUCUGUUGUUCCUAU-----UUUCUAU
```

uc002rji.2

```
Human          UACCC---GGAGGCUGGAUG-----AUGCUUGGGGGAAAGU---------------GUGAACAACUCAGC-UAAGC---U----CCCUGCC----UCCUG---UAG
Chimp          UACCC---GGAGGCUGGAUG-----AUGCUUGGGGGUAAGU---------------GUGAACAACUCAGC-UAAGC---U----CCCUGCC----UCCUG---UAG
Gorilla        UACCC---GGAGGCUGGAUG-----AUGCUUGGGGGUAAGU---------------GUGAACAACUCAGC-UAAGC---U----CCCUGCC----UCCUG---UAG
Orangutan      UACCC---GGAGGCUGGAUG-----AUGCUUGGGGGUGAGU---------------GUGAACAACUCAGC-UAAGC---U----CCCUGCC----UCCUG---UAG
Rhesus         UACCU---GGAGGCUGGAUG-----AUUCUUGGGGGUGAGU---------------GUGAACAACCCAGC-UAAGC---U----CCCUGCC----UCCUG---UAG
Baboon         UACCU---GGAGGCUGGAUG-----AUUCUUGGGGGUGAGU---------------GUGAACAACCCAGC-UAAGC---U----CCCUGCC----UCCUG---UAG
Marmoset       GACCU---AGAGACUGGAUG-----AUGCUUAGGGGUGAGU---------------GUGAACAACCCAGC-UAAGC---C----CACUGCC----UCCUG---UAG
Bushbaby       GACCU---GAUGCCCGAACG-----A-UGUUAGGAGUAAAU---------------GUAAGCAACCCAGC-UAAGC---U----CCCUGUC----UCCUG---CAG
TreeShrew      GGCCU---GGAGGCUGGAUG-----CUGUCCAUGGGUGUAU---------------GUGACCAGUCCGGC-UAAGU---G----CCCUGCC----UCCCU---UAG
Mouse          AGCCG---CGGGGC-GGAUG-----UCGCUUGUGGGUGAGU---------------GUGAGC-ACCCCAC-UGACC---C----UCCUGUC-----CCUG---CAG
Rat            AGCCGGGUUGGGGCUGGAUG-----UUGUCUCUGAGUGAAU---------------GUGACCGGCCCCAC-UGGAC---U----UCCUGUU-----CCCA---CAG
Kangaroo_rat   GGCUG---GGGAGCCAGGUG-----AUGUCUAUGAGUGAGU---------------GUGACCAACCCUGC-UAAGC---U----CCCUGCC----UCCCU---UAG
Guinea_Pig     GGGCU---GGAGCCUGAAUG-----AUGCUUGUGGAUGAAU---------------AUGACCAGUCCCAC-UAAGC---U----CCCUUCCUGGCUCCCU---UAG
Squirrel       GGCCU---AUAGGCUGGAUG-----GUGUUUGUAGGUGAAU---------------GUGACCAACCCUGC-UAAAC---U----CCCUCUC----CCUCU---UAG
Rabbit         GGCC----------GGGCG-----GUGUCGGUGCAGGAGC---------------GUGGCC-AGCCAGC-UAACC---C----CCCUGCC----UCCCU---CAG
Pika           GGCCG-------UCUGGGUG-----AUGCCUAGGAGCCAGC---------------ACGACC-AGCUGAC-UGAGU---G----UCCCACC----CCCUU---CAG
Alpaca         GGCC----AGAGGCUGGAUG-----A-UGUCAUGGGUGAGU---------------GUGACCAGUUCAGC-UAAGC---U----CCCUGUG-----CCA----UAG
Dolphin        GGCU----GGAGGCUGGAUG-----A-CGUCACGGGUGAGU---------------GUGAGCAACCCAGC-UAAGC---U----CCCUUUG-----CCCU---UAG
Cow            GGCU----GGAGGCCAGAUG-----G-CAUCAUGGGUGAGU---------------GUGCGCAACACAGC-UAAGC---U----CCCUGUG-----CCCU---UAG
Horse          GGCU----GGAGGCUAGAUG-----A-UGUCAGGGGU--GU---------------GUGACCAACCCAGC-UAAGC---U----CCCUGCA-----CCCU---UAG
Cat            GGCU----GGAGGCUAGAUG-----C-GGUCAGGGGUGAGU---------------GUGACCAACCCAG-----------------CUGCA-----CCCUUCGUAG
Dog            GGCU----GGAAGCUGGAUU-----A-AGUCAUGGUUGAGU---------------GUAACCAACCCAG-----------------CUGCA-----CCCU---UAG
Microbat       GGCU----GGAGGAU-------------GGCAUGGGUGAGU---------------GGGACCAACCGAGC-UAAGC---U----CCCUGCA-----CCCU---UAG
Megabat        GGCU----GGAGGAU-------------GUCAUGG----GU---------------GUGACCAAUCCAGC-UAAAC---UUCUGCUCUUCA-----CC-------G
Hedgehog       GGCG----GGAGACUGGGCG-----C-UGUCCCUGGCUAGU-----------------CACCAAC-CAGC-CAAGCCCUC----UCCUAUG-----CCCU---UAG
Elephant       GGGCU---GGAGGCUGGCUG-----AUGCCGGUGUGUGGGU---------------GUGACCAACCCAGC-UAAUC---U----CUCUGCC----ACCCU---CAG
Rock_hyrax     GGGCA---GGAGGCUAGCUG-----AUGCUGGUGAGUGGGU---------------UUGACCAACUGAAC-UAAGC---U----CUUUGCC----ACCCG---UAG
Tenrec         --GCU---GGAGG-UGGAUGGGACAGCGCUCAUGAGUGUGU---------------GUGGCCAGCCCAUC-UGAGC---C----CCCUGCC----GCCCC---CAG
Armadillo      GGGUG---GGGG---------------------GGGUGAGU---------------ACGGCCGGUCCCAC-UGAGC---U----CCCUGCC----GCCCU---CAG
Sloth          GGGCC---AGAGGCCACGGG-----GUAUCCAUGGGUGGGU---------------GUGGCCCAU-AUAC-UAAGC---U----CCCUG-C----ACCCU---CAG
Opossum        GC------AGAGCACAAGUG-----AGGCUCAGG-----GU---------------CUAACGGAUAUCAC-CUGAC---U----UCUCGCU----GUUCU---UAG
Wallaby        UGC-----AGAGCAUGAGUG-----A-------AUCUCUGU---------------CUGAAGACCACUGCUUGACC---U----CCUUGCU----UUUCU---UAG
Platypus       GGGCG---GGCGGGCGGGCG-----UGGUUCCUGGGCGGGUCCCGUGACGGUACCCGGGCCCUGCCCGAC-U--GC---C----GUCUGCG------CGC---CAG
```

uc003atr.19

```
Human         GUGGGCCUGGCCCC----AG-----GUUGGGGG-------------GA------------C--A--------CGGG---UGG----GUCC-CG-----------GCA-----CC-CCUCCCCUGACCACCGUGCC--UCU------CCCAG
Chimp         GUGGGCCUGGCCCC----AG-----GUUGGGGG-------------GA------------C--A--------CGGG---UGG----GUCC-CG-----------GCA-----CC-CCUCCCCUGACCACCGUGCC--UCU------CCCAG
Gorilla       GUGGGCCUGGCCCC----AG-----GUUGGGGG-------------GA------------C--A--------CAGG---UGG----GUCC-CA-----------GCA-----CC-CCUCCCCUGACCACUGUGCC--UCU------CCCAG
Orangutan     GUGGGCCUGGCCCC----AG-----GUUGGGGG-------------GA------------C--A--------CGGG---UGG----GUCC-CA-----------GCA-----CC-CCUCCCCUGACCACUGUGCC--UCU------CCCAG
Rhesus        GUGGGCCUGGCCCC----AA-----GUUGAGGG-------------GA------------C--A--------CGGG---CGG----GUCC-CA-----------GCA-----CC-CCUCCCCUGACCACCGUGCC--UCU------CCCAG
Baboon        GUGGGCCUGGCCCC----AA-----GUUGAGGG-------------GA------------C--A--------CGGG---CGG----GUCC-CA-----------GCA-----CC-CCUCCCCUGACCACCAUGCC--UCU------CCCAG
Marmoset      GUGGGCCUAGCCUC----AG-----GUUGGGGG-------------AA------------C--A--------UGGG---UGG----GUCC-CG-----------GCA------C-CCUGCCCUGACCACUGUGCC--CCU------CCCAG
Mouse_lemur   GUGGGCCUGGUCCC----GG-----GAAGGUGG--GC---------GG------------C--A--------UGGG---UGG----GUCC-C------------UCA-----UC-CCUG-CCUGACCAUCUCGCC---CU------CCCAG
Mouse         GUAGG-CGGGGCCCUGGCAG-----GCAGUGAG-------------UG------------G--G--------UGGG---ACU----GGCC-UG-----------GAG-----UCAGUCACCCUGACCGGCCUAUC---CU------CUCAG
Rat           GUAGA-CUGGCCCCCAGCAG-----GCUCUGAG-------------UA------------G--G--------CCAA---ACG----GGCC-UG-----------GGG-----UC-UCCGCCCUGACUGCCCUAUC---CU------CUCAG
Kangaroo_rat  GUGGGCCCGGCCCCUAUGGG-----GCUGGGGG-------------UG------------G--A--------CAUG---GUG----GGGC-CA-----------GCA-----UC--GAGGCCUAACCGUCCCUCCCCUCC-------CCCAG
Guinea_Pig    GUGGG-C---CUCGAAGCAG-----GCAUGGGC-------------UC------------G--GGCCGCCUUCCAG---CCU----GACCACC-----------GCG-----CCUCCUGCCCUGACCACUGCGCC--UCC------CGCAG
Squirrel      GUGGGCCUGGCCCGGGGGCGGGGCUGCAGGGGG-------------CG------------G--GGCUGC---AGGG---GCG----GGGCGGG-----------GCA-----GC-ACCCGCCUGACCGCGCCCCC---CU------C-CAG
Rabbit        GUGGGCCUGGGCGC-----G-----GCCGGGGG-------------GC------------G-------------G---GGC----GGCC-UG-----------GAG-----CC-CCCGCCCUGACCACGCCGCC---CC------C-CAG
Pika          GUGGGCCCAGCCCC----AG-----GAUGUGGG-------------AU------------GUUG--------UGCA---GGU----GGCC-UGGCCUUGCCUCUGCG-----CC-CCAACCCUGACCACUCCUCC---CCACCCCAC-CAG
Dolphin       GUGGGCCUGGCCCC-GCUGG-----GGUGGGGG-------------UGGGGCGUGGGCAUCC--C--------GGCG-------------------------------------CC-CCUGCACUGACCAUCCCACC--CCU------CCCAG
Cat           GUGAGCCUGGCCCG----GG-----GUGGGGGG-------------CU------------C--C--------GGGG-ACGGC----GGUC-CA-----------UCG-----UC-CCCGCGCUGACUGUCCC-UG--CCC------CCCAG
Dog           GUGAGCCUGGCCCG----GG-----GUCGGGGG-------------CA------------C--C--------GGGGCACAGC----AGUC-CA-----------UCA-----AC-CCUGCGCUGACCAUCCUGUG--CCU------CCCAG
Microbat      GUGGGCCUGGCCCC----AG-----UGGGUGGG-------------GA------------C--G--------GGCG---------------CC-CA-----------GCA-----GC-CCUGCGCUGACCAUCCC-CC--UCC------CCCAG
Megabat       GUGGGCCUGGCCCC----GG-----GCUGGGGG-------------UG------------U--G--------AGUG-GC-----------CC-CG-----------GCG-----GC-CUUGCUCUGACCAUCUC-CC--GCC------CCCAG
Hedgehog      GUGGGCCCCACCCC----AC-----GUUGGAGA-------------CC------------A--U--------GCGG---CGCCGGCGGCC-CG-----------UCG-----UC-CCCACACUGACUGUCCCCCA--ACC------ACUAG
Shrew         GUGGG-CCCGGCCC----GC-----GGCGGGGAGGGG---------CG------------G--G--------GAGG---GGCAG--GGCG-CG-----------GCG-----------CGCGCUGACC-CCCGCCG--CCC------GCCAG
Elephant      GUUGGCCUGGCCCC----CA-----GUAGGGUG-------------GG------------A--G--------UGGG---UGG----UCCC-CG-----------GCA-----CC-UCUGCACUGACUGUCCCUCC----C------CCCAG
Wallaby       GUAGGAGGAGUCCC----AG-----GGCAGG--GGGGGAGCCAGCACG------------C--A--------GAAG---GGU----GUCC-CA-----------GCACAGAACC-CAUUCCCACACCA----------------------CCAG
```

uc004bvw.9

```
Human          GUGUGGAGGGAA------------U-GGGGGCUAUGUG-UGAG-GG---AGCAGGGCUG------------GGG-----------------UCCC-------------GGGA---CU-AAG---C--UCC--CCCUU-UUC-UCCCAG
Chimp          GUGUGGAGGGAA------------U-GGGGGCUAUGUG-UGAG-GG---AGCAGGGCUG------------GGG-----------------UCCC-------------GGGA---CU-AAG---C--UCC--CCCUU-UUC-UCCCAG
Gorilla        GUGUGGAGGGAA------------U-GGGGGCUAUGUG-UGAG-GG---AGCAGGGCUG------------GGG-----------------UCCN-------------GGGA---CU-AAG---C--UCC--CCCUU-UUC-UCCCAG
Orangutan      GUGUGGAGGGAA------------U-GGGGGCUAUGUG-UGAG-GG---AGCAGGGCUG------------GGG-----------------UCCC-------------GGGA---CU-AAG---C--UCC--CCGUU-UUC-UCCCAG
Rhesus         GUGUGGAGGGAA------------U-GGGGGCUAUGUG-CGAG-GG---AGCACGGCUG------------GAG-----------------CCCC-------------GGGA---CU-AA----C--GUC--CCCUU-UUC-UCCCAG
Baboon         GUGUGGAGGGAA------------U-GGGGGCUAUGUG-CGAG-GG---AACAGGGCUG------------GGG-----------------CCCC-------------GGGA---CU-AAG---G--UCC--CC-UU-UUC-UCCCAG
Marmoset       GUGUGGAGAGAA------------U-GGGGGUUGUGUA-CGAA-UG---AGUAGGGCUG------------GGG-----------------CCCUGGG----------GGGA---CU-GAG---C--UCC--CCUUU-UUC-UCCCAG
Mouse_lemur    GUGUGGCGGGCG------------U--GGGGCUCUGCG-UGUG-GG---GGCGGGGCUG------------GGC-----------------CCCCAGGGGCC-----CUGGA---CU-GAG---C--UCC--CCCUU-GUC--CCCAG
TreeShrew      GUGUGGAGGGGG------------C-AGAG-----GUG-UGGA-GG---GGCAGGGCUGUACAUGGAGGGAGGG-----------------CUUUAGGGGCAUCUCGCCAGG---CU-GAG---C--UCC--CUGUC-UGC-CCCCAG
Mouse          GUGUG--GAGCA------------U-ACGGACUGUGUG-AGGA-GA--GGGCUGGGCUG--------------A-CCAAUGGGGA------UGCU-------------GGGA---CU-GAG---C--CCC--GCCUGCCUC-CUCUAG
Rat            GUACG--AAGGG------------U-UCAAACUGUGUG-ACGA-GA----GCUGGGCUG--------------A-CCCCUGGGGA------UGCU-------------GGGA---CU-GAG---C--CUG--GUCUGCCUC-CUCUAG
Kangaroo_rat   GUAUG--GAAGG------------UGGGGGGCUGUGUG-UGGA-GG---GGCUGGG---------------------ACUCUCGGGG------UACU-------------GGGA---CU-GAG---C--CCA--CU----CUC-CUCCAG
Guinea_Pig     GUGUG--AAGGG------------C-UGGGGCCACAUG-UGGA-CAGGGGACGGGACUG------------GAG-UCCUCAGGUG------UCCU-------------GGGA---CU-GAC---C--UCC--UU----UUG-CCCCAG
Squirrel       GUGUGU-GGGGG------------C-AAGGGUUCUAUGCUGGA-GG--CGGCAGGUCUG------------GGG-CCACAAGG--------UCCU-------------GAGA---CCAGAU---G--CUC--CU----CUG-CCACAG
Rabbit         GUGUGC-AGGGG------------C-CAGGGCUGUGCA-UGGAUGG---GGCGGGGCUG------------GGG-CAUGGGGGGGGCGGUUCCCU-------------GGCA---CU-GAG---CCGCCC--UC----UCC-CCCCAG
Dolphin        GUGUGGAGUGGA--------------GAAGGCUG-GCU-AGUA-GG---GGCAGGGCUG------------GGACCCUUAGGAGA------CCCC-------------GGGA---CU-GAG---C--CC---CC-UU-UUG-CCCCAG
Cow            GUAUG---------------------GAAGGCUG-GCG-AACA-GG---GGCAGGGCUG------------GGCCCCUCCAGAGA------CCUC-------------GAGA---CU-GAG---G--CC---GC-UU-CUG-UCCCAG
Horse          GUGUGGAGAGGA--------------GA-GGUGUGCA-UGUA-GG---GGCAGGGCAG------------GGCCCGUCAGGAGA------CCCC-------------GGGA---CU-GAG---C--CCC--CC-UU-UUGCUCCCAG
Cat            GUGUGGACGGGA--------------GACCUCUGUGCA-AUUA-GG---GGUAGGGCAG------------GGGCCCUCGGGGGG------CCGU-------------GGGA---CU-GAG---C--CCCACC-UU-UUG-UCCCAG
Dog            GUGUGGCCAGGA--------------GACAGCUUUGCA-GUUA-GG---GGCAGGGCAG------------GGGCUAUU-GGGGG------CCCC-------------AGGA---CU-GAG---U--CCU--CC-UU-UUG-UCCCAG
Microbat       GUACGGACAGGA--------------GAGGGCUGUGUA-CGUG-GG---GAGAGGGCAG------------GGG-GGUCAGGGGG------CCCC-------------GGGA---CU-GAA---C--CUC--CC-UU-UGG-CCGCAG
Megabat        GUAUGGACAGGA--------------GAGGGUUUUGUG-UGUA-GG---GACAGGGCAG------------GGG-UGUCAGGGGG------ACCC-------------AGGA---CU-GAA---C--CCU--UC-UU-UUG-CUCCAG
Elephant       GUACGAAAGGGG------------U-GAGAGCUGUGCG-UGUA-GG---GGUACGGCUG------------GGC-CGUUGGGGG-------CCCC-------------AGGA---CU-AAG---U--CCC--UUCUU-GCU-CCUCAG
Rock_hyrax     GUGUGGAAUGGG------------C-AAGGGCUGUGUG-UGCA-GG---GGCAGGGCUG------------GGC-UGUCAGGGA-------CCCC-------------AGGA---CU-CAG---C--CCC--UCGUU-GCU-CCUCAG
Tenrec         GUAUGGGGCCGG------------G-GAGGGCUG--------------------GGCGG------------AGC-CUUCGGGGC-------GCCC-------------CGGC---CU-GAGCACC--CUC--UCAUU-GGC-CCCCAG
Armadillo      GUGUGGAGGGGG------------U-GAGGGCUGUGUG-AGGA-GG---GGCGGGGCUG------------GGG-CGGUCAGUU-------CUCA-------------GGGU---CU-GAG--CC--CUC--CACUU-UUC-CCUCAG
Wallaby        GUGAGAGUCUGG------------U-GGAGGCGAGGGG-UGGG-GG---U-------------------------------------------------------GGAGGGGG---CU-GAG---C--UGA--CCCAU-UUC-UUCCAG
Platypus       GUGAGGGGAGACGUAGCGUGGCGCC-GGGGGGAGUCUG-GGGA-GG---UGGAGGGCUG------------GAG------AGGGG------CCGU-------------GGGAGGGCG-GAG---G--UCC--CACGG-CCC-CCUCAG
```

uc011maw.1

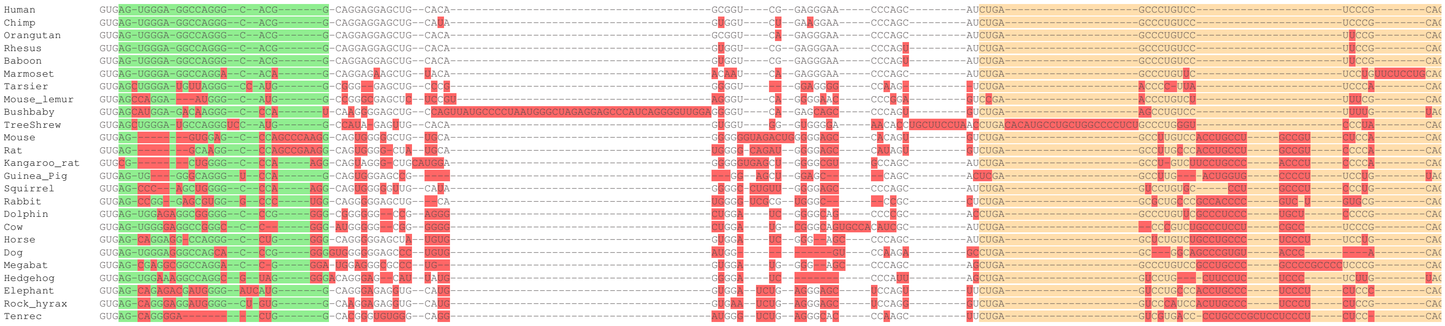
Human
Chimp
Orangutan
Rhesus
Baboon
Marmoset
Tarsier
Mouse_lemur
Bushbaby
TreeShrew
Mouse
Rat
Kangaroo_rat
Guinea_Pig
Squirrel
Rabbit
Dolphin
Cow
Horse
Dog
Megabat
Hedgehog
Elephant
Rock_hyrax
Tenrec
