# Supplementary material for: Analysis of Nearly One Thousand Mammalian Mirtrons Reveals Novel Features of Dicer Substrates
Source: PLoS Comput Biol. 2015 Sep 1;11(9):e1004441. doi: 10.1371/journal.pcbi.1004441 (PMC4556696; doi:10.1371/journal.pcbi.1004441)
Supplement: S3 Fig — (A, B) Plot of the maximum number of reads that were mapped to mouse and human mirtrons across individual libraries (x-axis: maximum #reads in individual libraries; y-axis: #mirtrons). (C, D) Plot of the maximum number of reads that were mapped to mouse and human mirtrons across grouped sets of similar tissues or cell lines (x-axis: maximum #reads in different tissues/cell lines; y-axis: #mirtrons). (E, F) Expressed mirtron distribution across sRNA-seq libraries, in comparison to canonical miRNAs. Considering mirtrons with greater than 5 RPM and canonical miRNAs with greater than 20 RPM in a single sample as expressed miRNAs, we observed that a majority of mirtrons expressed in 2–5 samples, indicating mirtrons tend to be specifically expressed. By contrast, canonical miRNAs have a different distribution with a peak of miRNAs expressed in > = 100 samples in both human and mouse, and a peak of 2–5 samples in mouse and of 11–20 samples in human, consistent with both broadly expressed and specifically expressed canonical miRNAs. Other cutoffs show a similar trend. (PDF) [file pcbi.1004441.s003.pdf]

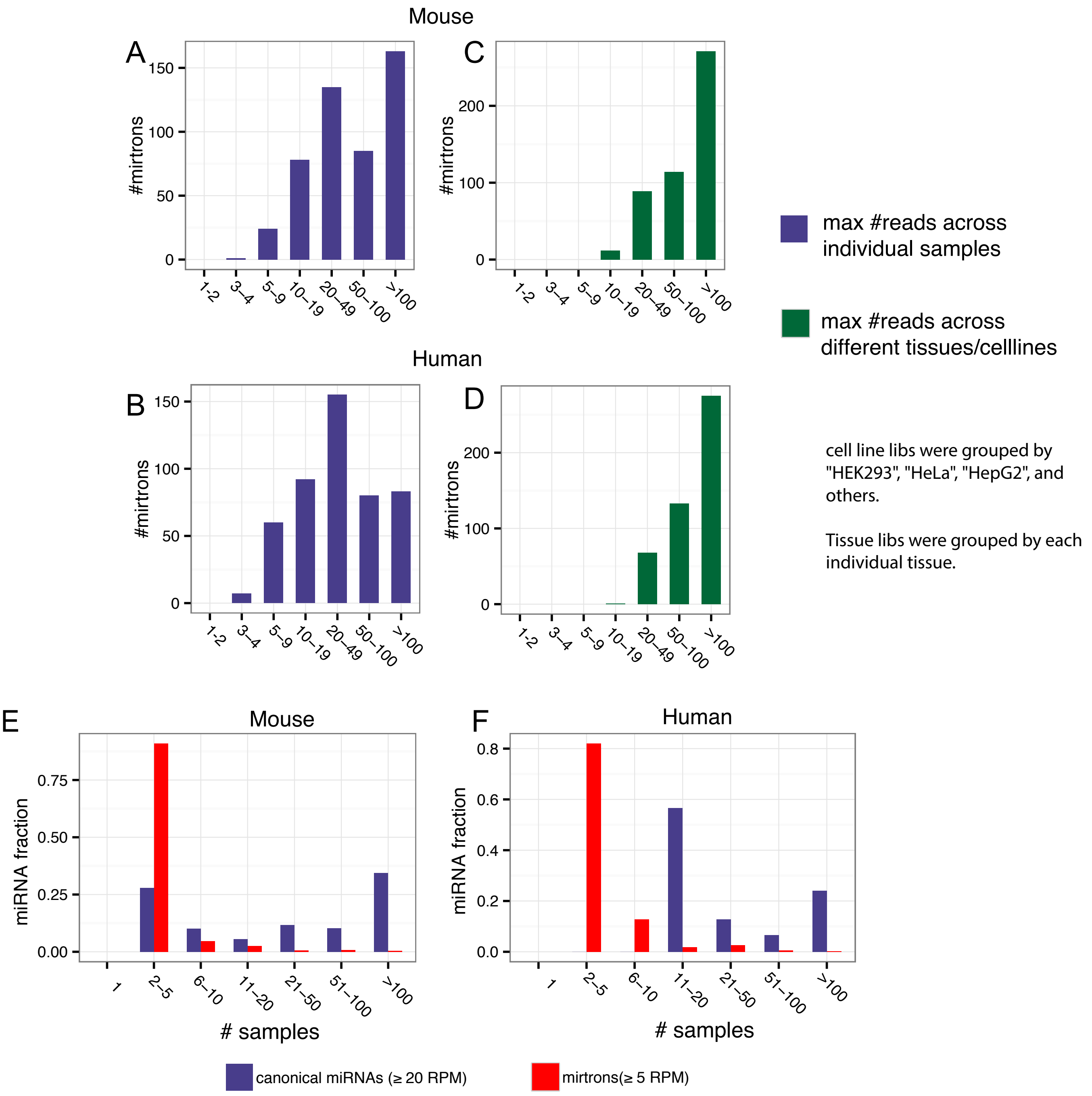


Distribution of canonical miRNAs and mirtrons across sRNA-seq samples.

(A, B) Plot of the maximum number of reads that were mapped to mouse and human mirtrons across individual libraries (x-axis: maximum #reads in individual libraries; y-axis: #mirtrons).
(C, D) Plot of the maximum number of reads that were mapped to mouse and human mirtrons across grouped sets of similar tissues or cell lines (x-axis: maximum #reads in different tissues/cell lines; y-axis: #mirtrons).
The human sRNA-seq tissue libraries were grouped into brain, testes, colon, ovary, cervix, B cell, breast, blood, liver, uterus, skin, heart, frontal cortex, cerebellum, kidney. The mouse sRNA-seq tissue libraries were grouped into brain, testes, whole newborn, oocyte, liver, skin, ovary, uterus, embryo, B cell, blood, bone marrow, spleen, thymus, lymph, heart, lung, kidney, pancreas, salivary gland, jejunum, epididymis, muscle, cerebellum, sertoli cells, spermatids, spermatocytes. The human sRNA-seq cell line libraries were grouped into HeLa, HepG2, HEK293, ESC, and others. The mouse sRNA-seq cell line libraries were grouped into MEF, ESC, MSC, and others.
(E, F) Expressed mirtron distribution across sRNA-seq libraries, in comparison to canonical miRNAs. Considering mirtrons with greater than 5 RPM and canonical miRNAs with greater than 20 RPM in a single sample as expressed miRNAs, we observed that a majority of mirtrons expressed in 2-5 samples, indicating mirtrons tend to be specifically expressed. By contrast, canonical miRNAs have a different distribution with a peak of miRNAs expressed in >= 100 samples in both human and mouse, and a peak of 2-5 samples in mouse and of 11-20 samples in human, consistent with both broadly expressed and specifically expressed canonical miRNAs. Other cutoffs show a similar trend.

Wen and Ladewig
Supplementary Figure 3
