# Supplementary material for: Analysis of Nearly One Thousand Mammalian Mirtrons Reveals Novel Features of Dicer Substrates
Source: PLoS Comput Biol. 2015 Sep 1;11(9):e1004441. doi: 10.1371/journal.pcbi.1004441 (PMC4556696; doi:10.1371/journal.pcbi.1004441)
Supplement: S4 Fig — We compared the expression of mirtrons and host mRNAs across various human and mouse tissues. For 399 mouse mirtrons small RNA and mRNA were compared from brain, heart, kidney, liver, lung, skeletal muscle, and spleen samples. For 223 human mirtrons, the tissues used were brain, breast, heart, kidney, liver, white blood cells. We utilized two strategies for assessing mRNA expression, by quantifying the union of all gene exons (A, C), as well as a more restricted analysis using only the reads that specifically span the mirtron-containing intron (B, D). The expression of each mirtron in each tissue was quantified as the number of reads per million mapped miRNA reads. We calculated the Pearson correlation coefficient for each mirtron:mRNA pair across tissues. We plotted their distribution as a cumulative distribution (upper row), and contrasted this with 100 permutations in which the analysis is repeated with shuffled tissue-labels. In the lower row the observed distribution of correlation coefficients is represented as a histogram. A two-tailed 1-sample Wilcoxon test was used to assess the significance of the observed deviation of the distribution median from zero. In all comparisons there is a statistically significant shift of the median of (mirtron:host) correlation towards positive correlation values. In the mouse data, the shift is less pronounced when using all host gene exons (A) than when using only the spliced mRNA reads that traverse the mirtron-containing intron (B), likely reflecting the latter is a more specific comparison. This trend was not observed in human, likely due to undersampling of these highly specific spliced reads (the mouse data are about an order of magnitude deeper than the human mRNA-seq data). This can be observed in the individual gene examples provided in S5 Fig. (PDF) [file pcbi.1004441.s004.pdf]

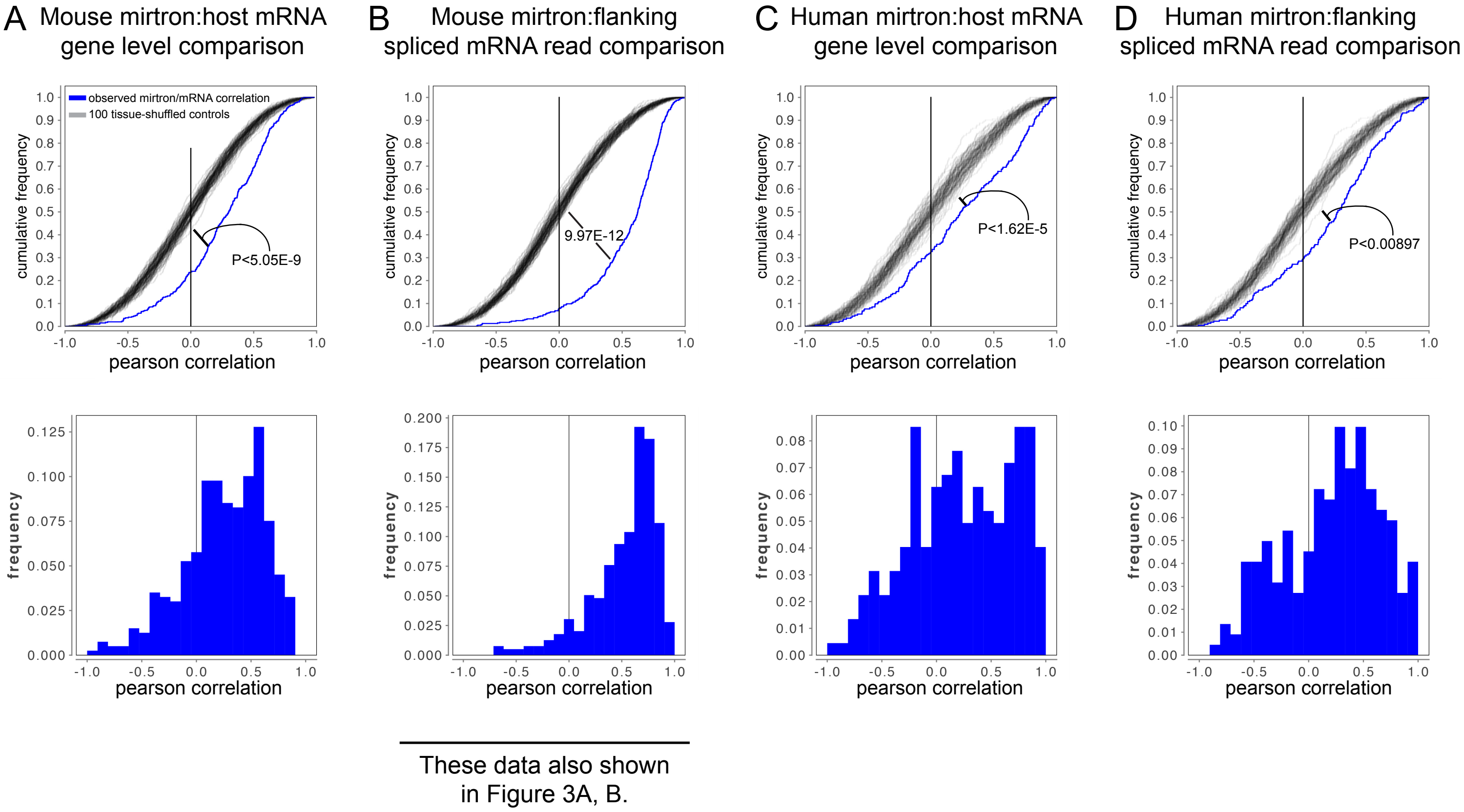

Correlation of mirtron and host gene across tissues in human and mouse.

We compared the expression of mirtrons and host mRNAs across various human and mouse tissues. For 399 mouse mirtrons small RNA and mRNA were compared from brain, heart, kidney, liver, lung, skeletal muscle, and spleen samples. For 223 human mirtrons, the tissues used were brain, breast, heart, kidney, liver, white blood cells. We utilized two strategies for assessing mRNA expression, by quantifying the union of all gene exons (A, C), as well as a more restricted analysis using only the reads that specifically span the mirtron-containing intron (B, D). The expression of each mirtron in each tissue was quantified as the number of reads per million mapped miRNA reads.

We calculated the Pearson correlation coefficient for each mirtron:mRNA pair across tissues. We plotted their distribution as a cumulative distribution (upper row), and contrasted this with 100 permutations in which the analysis is repeated with shuffled tissue-labels. In the lower row the observed distribution of correlation coefficients is represented as a histogram. A two-tailed 1-sample Wilcoxon test was used to assess the significance of the observed deviation of the distribution median from zero.

In all comparisons there is a statistically significant shift of the median of (mirtron:host) correlation towards positive correlation values. In the mouse data, the shift is less pronounced when using all host gene exons (A) than when using only the spliced mRNA reads that traverse the mirtron-containing intron (B), likely reflecting the latter is a more specific comparison. This trend was not observed in human, likely due to undersampling of these highly specific spliced reads (the mouse data are about an order of magnitude deeper than the human mRNA-seq data). This can be observed in the individual gene examples provided in S5 Fig.

Wen and Ladewig
Supplementary Figure 4
