# Supplementary material for: Analysis of Nearly One Thousand Mammalian Mirtrons Reveals Novel Features of Dicer Substrates
Source: PLoS Comput Biol. 2015 Sep 1;11(9):e1004441. doi: 10.1371/journal.pcbi.1004441 (PMC4556696; doi:10.1371/journal.pcbi.1004441)
Supplement: S5 Fig — Four rows of data are shown for each locus: (1) mirtron- miRNA reads (in reads per million mapped miRNA reads, RPMM), (2) spliced mRNA-seq reads that directly traverse the mirtron-containing intron (in reads per million mapped, RPM), (3) mRNA-seq reads in the two flanking exons upstream and downstream of the mirtron-containing intron (in reads per kilobase of transcript per million mapped reads, RPKM), and (4) mRNA-seq reads in the entire mirtron host gene model (in RPKM). (A) Mouse data. (B) Human data. For both species, examples of well-correlated as well as discordant mirtron and host mRNA expression patterns are shown. Note that the available human mRNA-seq data were nearly an order of magnitude smaller than the those in mouse; thus, the measurements of spliced reads across mirtron-containing introns are adversely affected in some cases. While the overall patterns are similar amongst most of the mRNA measurements, no spliced reads were recovered for WA5B2(hsa-mir-1224), indicating undersampling. As well, the pattern of spliced TBC1D24(uc002cqk.5) reads is discordant with other measures of mRNA expression, and could represent an undersampling artifact. (PDF) [file pcbi.1004441.s005.pdf]

A Examples of mouse mirtron: host mRNA correlation across tissues

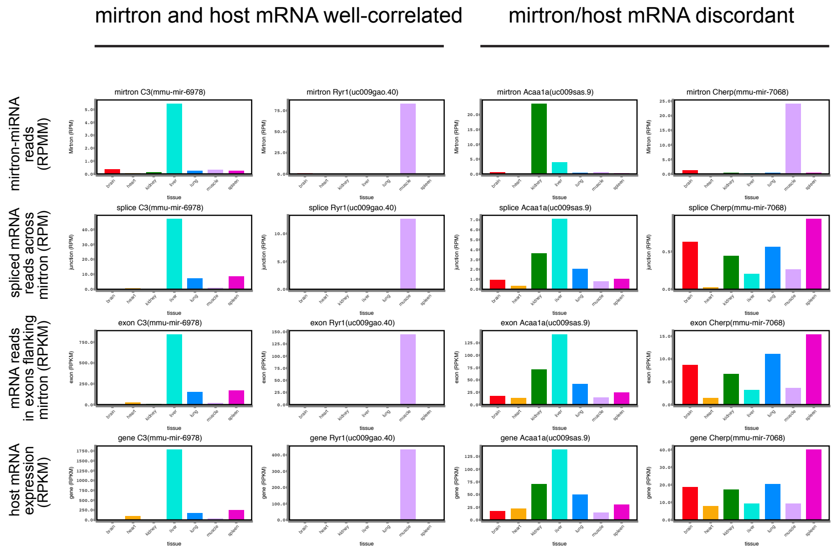

B Examples of human mirtron: host mRNA correlation across tissues

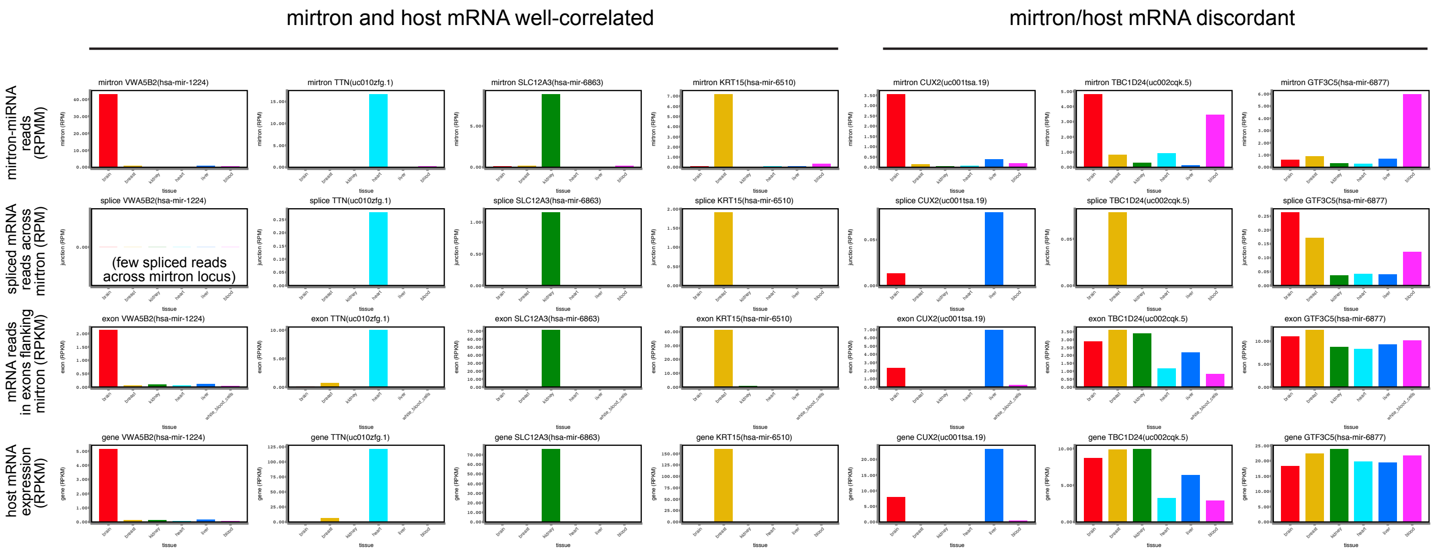

Examples of mirtron: host mRNA correlation across tissues. Four rows of data are shown for each locus: (1) mirtron- miRNA reads (in reads per million mapped miRNA reads, RPMM), (2) spliced mRNA-seq reads that directly traverse the mirtron-containing intron (in reads per million mapped, RPM), (3) mRNA-seq reads in the two flanking exons upstream and downstream of the mirtron-containing intron (in reads per kilobase of transcript per million mapped reads, RPKM), and (4) mRNA-seq reads in the entire mirtron host gene model (in RPKM). (A) Mouse data. (B) Human data. For both species, examples of well-correlated as well as discordant mirtron and host mRNA expression patterns are shown. Note that the available human mRNA-seq data were nearly an order of magnitude smaller than the those in mouse; thus, the measurements of spliced reads across mirtron-containing introns are adversely affected in some cases. While the overall patterns are similar amongst most of the mRNA measurements, no such spliced reads were recovered for *WA5B2(hsa-mir-1224)*, indicating undersampling. As well, the pattern of splice*d TBC1D24(uc002cqk.5)* reads is discordant with other measures of mRNA expression, and could represent an undersampling artifact.

Wen and Ladewig
Supplementary Figure 5
