# Supplementary material for: Analysis of Nearly One Thousand Mammalian Mirtrons Reveals Novel Features of Dicer Substrates
Source: PLoS Comput Biol. 2015 Sep 1;11(9):e1004441. doi: 10.1371/journal.pcbi.1004441 (PMC4556696; doi:10.1371/journal.pcbi.1004441)
Supplement: S6 Fig — (A) Comparison of mirtron-bearing introns with total introns in mouse. The distribution of total intron lengths is much broader than for mintrons. The dominant class of 5' tailed mirtrons derives mostly from introns that are <3kb in length, while the 3'-tailed mirtrons and conventional mirtrons derive from very short introns. (B, C) Nucleotide bias of small RNAs from 5'-tailed mirtrons. (B) Biased nucleotide identities of mirtron-5p reads from the dominant class of 5'-tailed mirtrons. Compared to an equivalent sequence range of control introns of similar length, mirtron-5p reads exhibit substantial 5'-U bias and overall enrichment of G across their lengths. The G bias is greater in the 5' than 3' regions of the mirtron-5p reads, and is not evident in bulk intron sequences downstream of their ~22 nt lengths. (C) Biased nucleotide identities of mirtron-3p reads from the dominant class of 5'-tailed mirtrons. Compared to control introns, there is substantial 5'-U bias (evident with aligning by their 5' ends) and substantial C-bias across their length. Note that the bulk introns exhibit polypyrimidine tracts upstream of the splice acceptor site (YAG), but mirtrons exhibit greater representation of C while control introns show greater bias for U. (D) Mirtronic regions exhibit much lower minimum free energy (MFE) than control intronic regions. CDF (cumulative distribution function) is plotted for MFE/base distribution. (E) All four classes of mirtrons are hosted by genes with greater numbers of introns than average genes. Various classes of other intronic non-coding RNAs (e.g. tRNAs, snoRNAs, and either conserved or non-conserved canonical miRNAs) typically reside in genes with larger numbers of introns than bulk genes, but their averages are intermediate to all classes of mirtrons. (F) Bar graphs that emphasize the individual properties of genes that host various classes of non-coding RNAs. It is evident that the all four classes of mirtrons have a broader distribution of [file pcbi.1004441.s006.pdf]

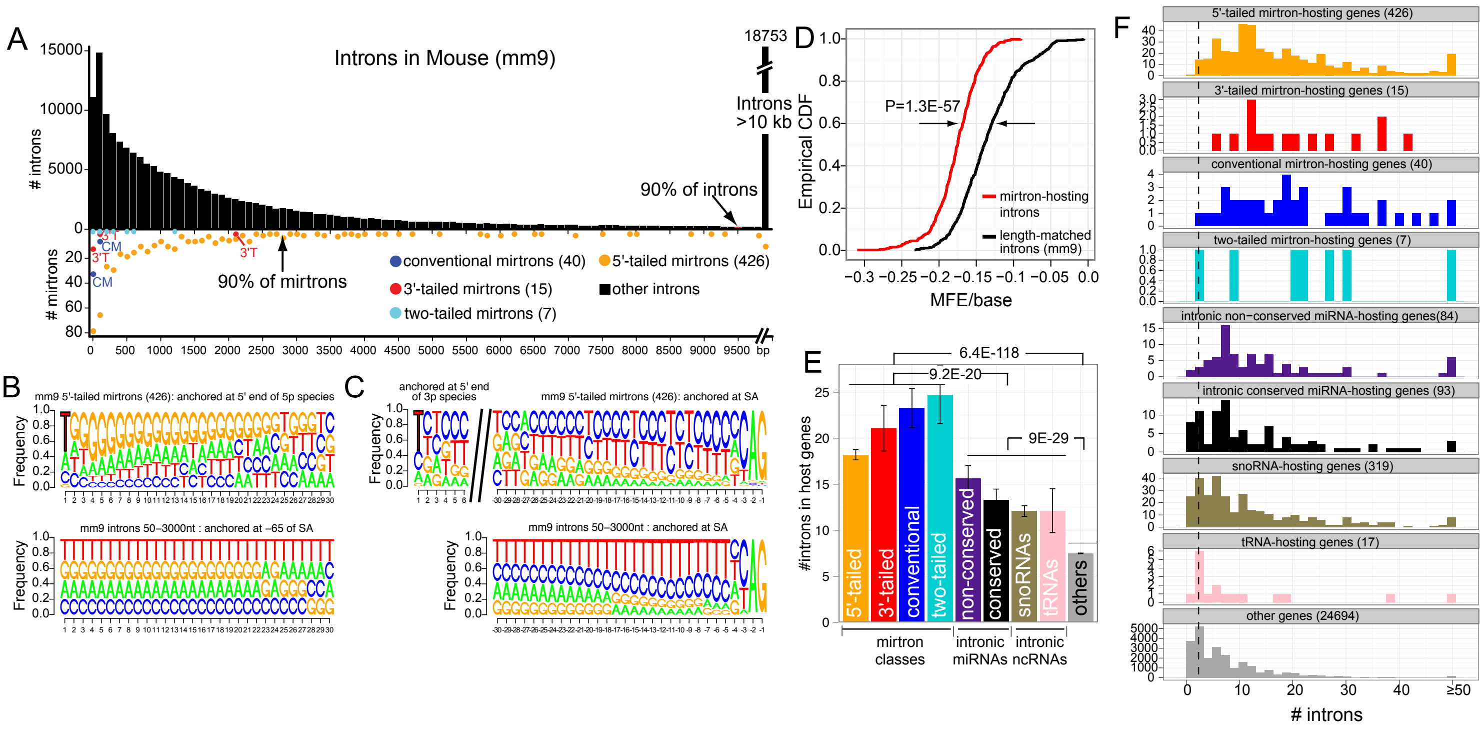

Sequence and length properties of mirtron-containing introns in mouse

(A) Comparison of mirtron-bearing introns with total introns in mouse. The distribution of total intron lengths is much broader than for mintrons. The dominant class of 5' tailed mirtrons derives mostly from introns that are <3kb in length, while the 3'-tailed mirtrons and conventional mirtrons derive from very short introns. (B, C) Nucleotide bias of small RNAs from 5'-tailed mirtrons. (B) ¬¬Biased nucleotide identities of mirtron-5p reads from the dominant class of 5'-tailed mirtrons. Compared to an equivalent sequence range of control introns of similar length, mirtron-5p reads exhibit substantial 5'-U bias and overall enrichment of G across their lengths. The G bias is greater in the 5' than 3' regions of the mirtron-5p reads, and is not evident in bulk intron sequences downstream of their ~22 nt lengths. (C) Biased nucleotide identities of mirtron-3p reads from the dominant class of 5'-tailed mirtrons. Compared to control introns, there is substantial 5'-U bias (evident with aligning by their 5' ends) and substantial C-bias across their length. Note that the bulk introns exhibit polypyrimidine tracts upstream of the splice acceptor site (YAG), but mirtrons exhibit greater representation of C while control introns show greater bias for U. (D) Mirtronic regions exhibit much lower minimum free energy (MFE) than control intronic regions. CDF (cumulative distribution function) is plotted for MFE/base distribution. (E) All four classes of mirtrons are hosted by genes with greater numbers of introns than average genes. Various classes of other intronic non-coding RNAs (e.g. tRNAs, snoRNAs, and either conserved or non-conserved canonical miRNAs) typically reside in genes with larger numbers of introns than bulk genes, but their averages are intermediate to all classes of mirtrons. (F) Bar graphs that emphasize the individual properties of genes that host various classes of non-coding RNAs. It is evident that the all four classes of mirtrons have a broader distribution of intron numbers relative to other types of non-coding RNAs.

Wen and Ladewig
Supplementary Figure 6
