# Supplementary material for: Analysis of Nearly One Thousand Mammalian Mirtrons Reveals Novel Features of Dicer Substrates
Source: PLoS Comput Biol. 2015 Sep 1;11(9):e1004441. doi: 10.1371/journal.pcbi.1004441 (PMC4556696; doi:10.1371/journal.pcbi.1004441)
Supplement: S7 Fig — Pie charts depicting the fractions of 3p-arm canonical miRNAs and mirtron-derived miRNAs that match the genome directly, or following trimming of 3' untemplated nucleotides. For both 5'-tailed and conventional mirtrons, reads ending precisely at the AG splice acceptor and reads extending beyond the AG splice acceptor were pooled from all loci. For canonical miRNAs, the reads ending precisely at and extending beyond the annotated 3p boundaries, which was defined by the most abundant matching read from all aggregated small RNA libraries analyzed in this study, were pooled from all loci. The pie charts illustrate categories of untemplated nucleotide additions with >1% of total reads; the remaining reads were classified as “others”. The vast majority of human and mouse canonical miRNA-3p reads match the genome. In contrast, the majority of mirtron-3p species bear untemplated additions, primarily uridylation and to a lesser extent adenylation. (PDF) [file pcbi.1004441.s007.pdf]

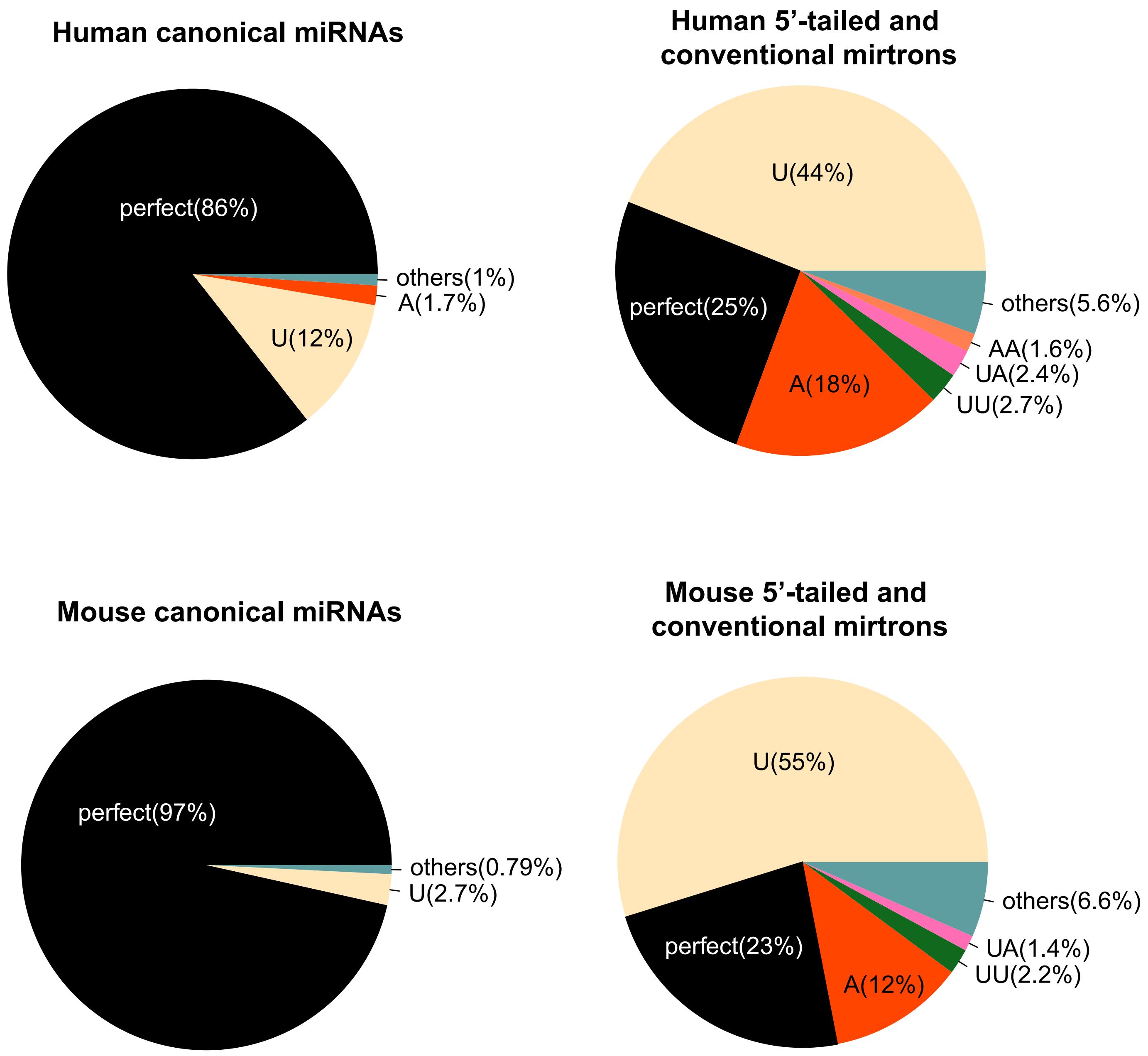

3' untemplated additions in mammalian canonical miRNA-3p and mirtron-3p species.

Pie charts depicting the fractions of 3p-arm canonical miRNAs and mirtron-derived miRNAs that match the genome directly, or following trimming of 3' untemplated nucleotides. For both 5'-tailed and conventional mirtrons, reads ending precisely at the AG splice acceptor and reads extending beyond the AG splice acceptor were pooled from all loci. For canonical miRNAs, the reads ending precisely at and extending beyond the annotated 3p boundaries, which was defined by the most abundant matching read from all aggregated small RNA libraries analyzed in this study, were pooled from all loci. The pie charts illustrate categories of untemplated nucleotide additions with >1% of total reads; the remaining reads were classified as “others”. The vast majority of human and mouse canonical miRNA-3p reads match the genome. In contrast, the majority of mirtron-3p species bear untemplated additions, primarily uridylation and to a lesser extent adenylation.

Wen and Ladewig
Supplementary Figure 7
