# Supplementary material for: Analysis of Nearly One Thousand Mammalian Mirtrons Reveals Novel Features of Dicer Substrates
Source: PLoS Comput Biol. 2015 Sep 1;11(9):e1004441. doi: 10.1371/journal.pcbi.1004441 (PMC4556696; doi:10.1371/journal.pcbi.1004441)
Supplement: S8 Fig — Since these are generated directly by splicing, such 5p reads should bear the 5'-GU splice donor sequence. We tally the frequency of unmodified GU reads, "guanine-decapitated" reads ("xU"), and other reads in this graph. Many mirtron hairpins are subject to substantial 5' decapitation. (PDF) [file pcbi.1004441.s008.pdf]

Mouse 5’ end of 5p arm of 3'-tailed/conventional mirtrons

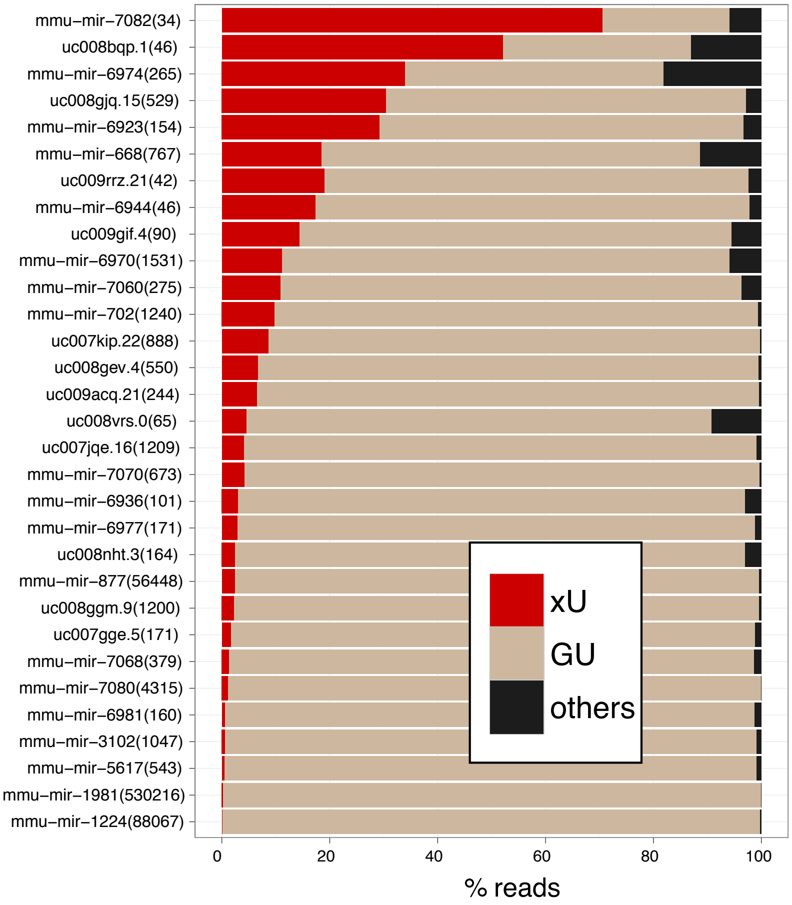


Summary of 5' heterogeneity amongst 5p reads from mouse conventional and 3'-tailed mirtrons. Since these are generated directly by splicing, such 5p reads should bear the 5'-GU splice donor sequence. We tally the frequency of unmodified GU reads, "guanine-decapitated" reads ("xU"), and other reads in this graph. Many mirtron hairpins are subject to substantial 5' decapitation.

Wen and Ladewig
Supplementary Figure 8
